# Magnetic Blocking in Fluoflavine Radical-Bridged Dilanthanide Complexes

**DOI:** 10.1021/jacs.5c14158

**Published:** 2025-12-10

**Authors:** Florian Benner, Saroshan Deshapriya, Jakub Hrubý, Stephen Hill, Selvan Demir

**Affiliations:** † Department of Chemistry, 3078 Michigan State University, East Lansing, Michigan 48824, United States; ‡ 189689 National High Magnetic Field Laboratory, Tallahassee, Florida 32310, United States; § Department of Physics and Department of Chemistry and Biochemistry, 7823 Florida State University, Tallahassee, Florida 32306, United States

## Abstract

Magnetic exchange coupling is difficult to foster in polynuclear lanthanide (Ln) complexes and poorly understood. While coupling Ln ions through closed-shell ligands is inherently weak due to the contracted 4f orbitals, placing open-shell ligands instead has proven to promote orders of magnitude stronger coupling, giving rise to single-molecule magnets (SMMs) innate to real magnetic memory effect in the case of the anisotropic Ln ions. Notably, the impact of radical bridges with differing oxidation states on magnetic blocking remains unexplored due to lack of Ln SMMs with radicals in two distinct charge states. Herein, the first dilanthanide complexes (Ln = Gd, Dy) containing fluoflavine (flv) bridges, [(Cp*_2_Ln)_2_(μ-flv^z^)]­X, (where X = [Al­(OC­{CF_3_}_3_)_4_]^−^ (*z* = 1–•), **1-Ln**; X = 0 (*z* = 2−), **2-Ln**; X =[K­(crypt-222)]^+^ (*z* = 3–•), **3-Ln**) are reported. **1-Ln** and **3-Ln**, comprising the flv^1–•^ and flv^3–•^ radical bridges, were investigated via single-crystal X-ray diffraction (SCXRD), ultraviolet–visible (UV–vis) spectroscopy, Superconducting Quantum Interference Device (SQUID) magnetometry, high-field electron paramagnetic resonance (HF-EPR) spectroscopy and broken-symmetry density functional theory (BS-DFT) calculations. **1-Dy** and **3-Dy** constitute the first SMMs innate to radicals in two differing oxidation states. **1-Dy** exhibits a spin-reversal barrier *U*_eff_ of 28.36 cm^–1^ and open magnetic hysteresis loops below 3 K. By contrast, **3-Dy** displays a much higher *U*_eff_ of 143(2) cm^–1^ and open hysteresis loops until 9.5 K, representing a record for dilanthanide SMMs containing an organic radical bridge. The boost in SMM properties in **3-Dy** is attributed to spin-phonon coupling and improved frontier orbital structure. This study paves the way for advanced design strategies of polynuclear Ln SMMs.

## Graphical abstract

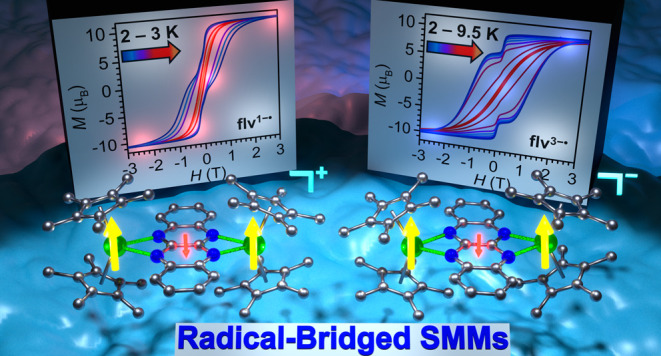

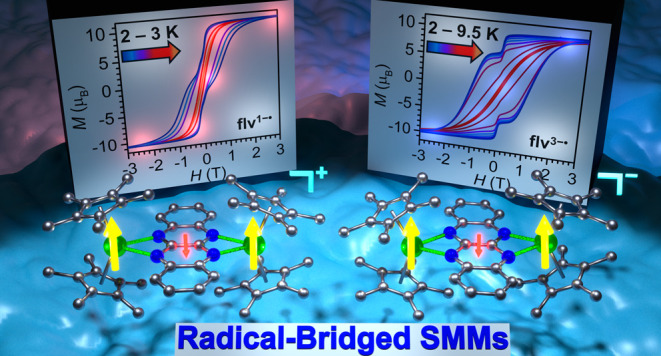

## Introduction

The development of novel information storage and processing media has received considerable attention in light of recent rapid advances in cloud-based applications and artificial intelligence.^1,2^ With the global data sphere projected to reach 175 zettabytes (1 zettabyte = 10^9^ terabyte) in 2025,^2,3^ the accompanied skyrocketing storage demands and energy consumption of data centers^4^ are the primary motivators to develop smaller and more energy efficient physical data storage blocks known as bits.

To tackle these demands, individual molecules have emerged as appealing targets to serve as bits. For instance, innovative approaches such as utilizing DNA strands and other small organic molecules as information carriers^3,5,6^ constitute promising avenues to decode information. However, these methods require considerable synthetic effort and are not yet competitive in terms of speed and reliability of the data writing and reading processes.^3^

The first molecule to show single-molecule magnet (SMM) behavior was a Mn_12_ acetate cluster complex.^7–9^ One key experimental observable for a SMM with notable magnetic memory effect is an open magnetic hysteresis loop arising from a variable-field magnetization measurement (*M* vs *H*), which in the case of Mn_12_ is open below 3 K with a considerable coercive field (*H*_C_) of ∼1 T at 2.2 K.^8^

The exploitation of highly anisotropic lanthanide ions, such as dysprosium­(III), as spin carriers boosted performance characteristics of SMMs where high magnetic blocking temperatures (*T*_B_) were recorded, even exceeding the boiling temperature of liquid nitrogen.^11–14^ In these mononuclear complexes, the Kramers Dy^III^ ion is sandwiched in between two five-membered aromatic rings, affording an axial coordination sphere that stabilizes the ground state *m*_*J*_ = ±^15^/_2_ Kramers doublet.^15,16^

To reach even higher blocking temperatures, strong magnetic coupling of multiple lanthanide ions must be fostered, which is challenging due to the well-shielded valence 4f orbitals inherent to the lanthanides.^17^ Thus, orbital overlap with diamagnetic ligands comprising lighter p-block donors is often weak, but can be substantially augmented through the introduction of either heavy p-block elements such as bismuth,^18–20^ metal–metal bonding,^21,22^ or radical bridging ligands with diffuse spin orbitals that can penetrate the deeply buried 4f shell.^23,24^ Specifically, in such radical-bridged Dy^III^ systems the magnetic exchange coupling strength is directly related to the relaxation barrier by *U*_eff_ = 15 |*J*|.^25,26^ The best dinuclear SMM containing a radical ligand as a bridge constitutes the dinitrogen radical anion, N_2_^3–•^, in the form of [K­(crypt-222)]­[(Cp^Me4H^_2_Tb)_2_(μ-N_2_^•^)] (where crypt-222 = 2.2.2-cryptand). Here, the coupling of two highly anisotropic terbium­(III) ions results in open magnetic hysteresis loops up to 30 K and a remarkable coercive field, *H*_C_, of 7.9 T, exceeding that of commercially available NdFeB.^25^

Strong magnetic coupling can also be achieved through organic radical ligands,^26–35^ with which more robust systems can be manufactured which allow chemical modifications to fine-tune magnetic properties. For instance, the functionalization of the radical ligand with electron donating- and withdrawing groups had an enormous effect on the magnetic relaxation as demonstrated in a series of 2,2′-bipyrimidine- and 1,2,4,5-tetrazine-bridged complexes.^26,36^ However, owing to the tiny number of radical-containing SMMs hitherto known, key design strategies are yet to be perfected: In depth understanding of the factors that govern magnetic relaxation and the interplay of magnetic coupling, magnetic anisotropy, employed metal ions and radical ligands will pave the way to viable functional molecular magnetic materials. To this end, the isolation of new compounds bearing unexplored radicals is required,^30,37–39^ yet synthetically challenging due to the difficulty of handling and taming highly reactive radicals.

This so-called lanthanide-radical approach exploited monoanionic radicals of bipyrimidine, pyrazine, and 3,6-substituted and unsubstituted tetrazines,^26,27,29,36^ and trianionic radicals of tetraoxolene, indigo and bisbenzimidazole,^30–32^ where appreciable coupling and slow magnetic relaxation was observed. Notably, radical-bridged SMMs with the radical existing in two differing oxidation states are completely unknown. There is only one study that reports the synthesis of radical-bridged dilanthanide complexes composed of two differing paramagnetic oxidation states for the radical where the hexadentate multielectron redox-active bridging ligand 2,3,5,6-tetra­(2-pyridyl)­pyrazine (tppz) was employed.^40^ The complexes [(Cp*_2_Dy)_2_(μ-tppz^•^)]­(BPh_4_) and K­[(crypt-222)]­[(Cp*_2_Dy)_2_(μ-tppz^•^)], containing the tppz^1–•^ and tppz^3–•^ radical bridges, exhibited similar coupling strengths, however, only the tppz^1–^^•^ bridged complex was a SMM.^40^ There is no other study that explores the impact of two charges of topologically similar radical bridges on magnetic coupling and SMM behavior. Recently, some of us discovered the first isolable radicals of 5,6,11,12-tetraazanaphthacene, TATC, which we referred to as fluoflavine in the zero oxidation state (flv^0^) for the basis frame inspired by its name for the doubly protonated version H_2_flv (Figure 1).^10,41^ Specifically, the flv^1–•^ and flv^3–•^ radical-bridged yttrium complexes [(Cp*_2_Y)_2_(μ-flv^•^)]­X, (where Cp* = pentamethylcyclopentadienyl, X = [Al­(OC­{CF_3_}_3_)_4_]^−^ and [K­(crypt-222)]^+^, respectively), were isolated.^10^ We hypothesized that the synthetic route should be transferable to the heavier lanthanides owing to similar ionic radii of Gd^III^/Dy^III^ and Y^III^, and similar RE^III^/RE^II^ redox potentials.^42–44^

**Figure 1:**
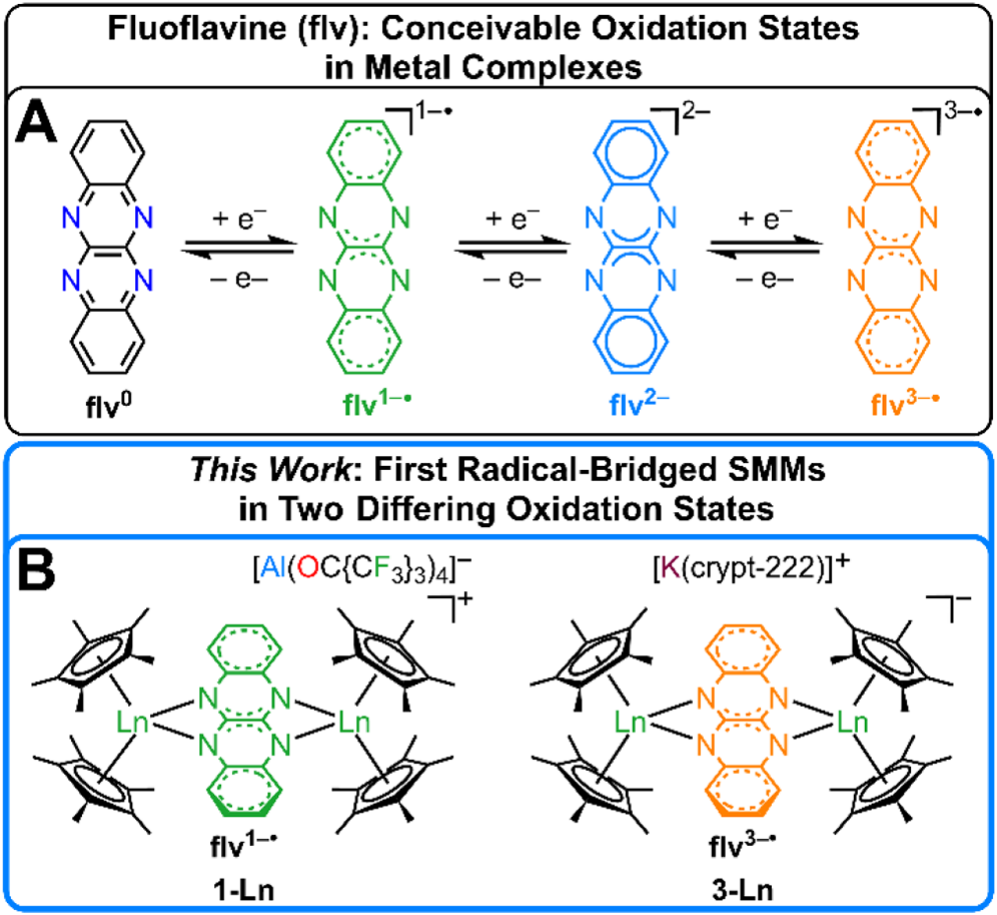
(A) Conceivable oxidation states of fluoflavine (quinoxalino-[2,3-*b*]­quinoxaline) in metal complexes.^10^ (B) The first fluoflavine radical-bridged SMMs [(Cp*_2_Ln)_2_(μ-flv)]­X (**1-Ln** and **3-Ln**) where Ln = Gd, and Dy, X = [Al­(OC­{CF_3_}_3_)_4_]^−^ or [K­(crypt-222)]^+^, bearing flv^1–•/3–•^, respectively.

Herein, we present the synthesis, and in-depth characterization of the first two sets of fluoflavine radical-bridged dilanthanide complexes: The first set [(Cp*_2_Ln)_2_(μ-flv^•^)]­[Al­(OC­{CF_3_}_3_)_4_] (**1-Ln** = Gd, Dy) containing a flv^1–•^ bridge was obtained from chemical oxidation of the neutral complexes [(Cp*_2_Ln)_2_(μ-flv)] (**2-Ln** = Gd, Dy) with [Thian^•^]­[Al­(OC­{CF_3_}_3_)_4_]. The second set [K­(crypt-222)]­[(Cp*_2_Ln)_2_(μ-flv^•^)] (**3-Ln** = Gd, Dy) comprising a flv^3–•^ bridge was generated via chemical reduction of **2-Ln** using the strong reductant KC_8_ in the presence of chelating crypt-222. Importantly, **1-Ln** and **3-Ln** represent the first fluoflavine radical-bridged compounds that contain paramagnetic metal ions of any type. Simultaneously, **1-Ln** and **3-Ln** represent the first radical-bridged SMMs for any metal ions composed of two differing paramagnetic oxidation states for the radical. The complexes **1-Dy** and **3-Dy** feature open magnetic hysteresis loops, with a doubled coercive field and more than a 3-fold increase in hysteresis temperature for the flv^3–•^ radical-bridged Dy SMM relative to **1-Dy**. In fact, the temperature of 9.5 K at which the hysteresis is still open for **3-Dy** constitutes a record for dilanthanide SMMs containing organic radicals. Furthermore, the Gd congeners allowed magnetic coupling strength determination and broken-symmetry density functional theory (BS-DFT) calculations that altogether provide a blueprint for future SMM design.

## Experimental Section

### Experimental Materials and Methods

All manipulations were performed under inert conditions using either standard Schlenk techniques employing nitrogen atmosphere or an argon-filled glovebox. House nitrogen was purified through a MBraun HP-500-MO-OX gas purifier. ^*n*^Hexane and dichloromethane (DCM) were purified by refluxing over CaH_2_, toluene and tetrahydrofuran (THF) were purified by refluxing over potassium and subsequent distillation. THF was subsequently stirred over NaK for at least a day and distilled a second time prior to use. In all cases except for DCM, the solvents were tested for the presence of water and oxygen in the glovebox by the addition of one drop of potassium benzophenone radical solution to 2 mL of the solvent of interest.

The chemicals pentamethylcyclopentadiene (Cp*H), allylmagnesium chloride (2.0 M in THF), and anhydrous LnCl_3_ (where Ln = Gd, Dy) were purchased from Sigma-Aldrich and used as received. Potassium bis­(trimethylsilyl)­amide (KN­(Si­(CH_3_)_3_)_2_) and 2.2.2-cryptand (crypt-222) were purchased from Sigma-Aldrich. KN­(Si­(CH_3_)_3_)_2_ was recrystallized from toluene, and crypt-222 from ^*n*^hexane prior to use. KCp*,^45^ [HNEt_3_]­[BPh_4_],^46^ Cp*_2_Ln­(BPh_4_),^45^ H_2_flv,^47^ K_2_flv,^10^ thianthrenium tetra­(perfluoro­(*tert*-butoxy))­aluminate [Thian^•^]­[Al­(OC­{CF_3_}_3_)_4_],^48^ and KC_8_,^49^ were synthesized according to literature procedures.

### X-ray Crystallography

Single-crystal XRD data on **1-Ln**, **2-Ln**, and **3-Ln** were collected on a XtaLAB Synergy DualflexHyPix four-circle diffractometer, equipped with a HyPix Hybrid Pixel Array Detector. The crystals were suspended in ^n^Paratone oil and mounted on a nylon loop. The temperature during data collection was controlled via an Oxford Cryosystems low-temperature device and kept at 100 K during the measurements for all compounds except for **2-Dy**, which was collected at 220 K due to rapid crystal degradation in the N_2_ stream at lower temperatures. Data were measured using ω scans using Cu Kα radiation (microfocus sealed X-ray tube, 50 kV, 1 mA). The CrysAlisPro software package^50^ was used to determine the total number of runs and images, to retrieve and refine the cell parameters, and for data reduction. A numerical absorption correction based on Gaussian integration over a multifaceted crystal model empirical absorption correction using spherical harmonics was done using the SCALE3 ABSPACK^51^ scaling algorithm (spherical harmonics and frame scaling). The structures were solved with the ShelXT^52^ structure solution program using intrinsic phasing and refined with version 2018/3 of ShelXL^53^ using least-squares minimization as implemented in Olex2.^54^ All non-hydrogen atoms were refined anisotropically. Hydrogen atoms were calculated by geometrical methods and refined as a riding model. The crystals used for the diffraction study showed no decomposition during data collection. Crystal data and structure refinement for all compounds are shown in Tables S1 and S2.

### Spectroscopy

IR spectra were taken with an Agilent Cary 630 ATR spectrometer under a nitrogen atmosphere.

Ultraviolet–visible (UV–vis) absorption spectra were collected in an argon-filled glovebox using 1 cm cuvettes with an Agilent Cary 60 spectrometer, equipped with QP600–1-SR fiber optics and a Square One cuvette holder from Ocean Insight. Solvents and concentrations were 34.9 μmol/L in DCM (**1-Gd**), 18.9 μmol/L in DCM (**1-Dy**), 25.2 μmol/L in THF (**2-Gd**), 28.4 μmol/L in THF (**2-Dy**), 28.6 μmol/L in THF (**3-Gd**), and 35.6 μmol/L in THF (**3-Dy**).

### Electron Paramagnetic Resonance

Temperature- and frequency-dependent high-field electron paramagnetic resonance (HF-EPR) studies were conducted in order to determine spin Hamiltonian parameters for **1-Gd, 2-Gd**, and **3-Gd**. The spectra were collected using a home-built transmission spectrometer that has been described in detail previously.^55^ The instrument employs magnetic field modulation, thus resulting in derivative-mode spectra (d*I*/d*B*, where *I* is the microwave intensity transmitted through the sample and *B* is the local magnetic field). All samples were measured as ground polycrystalline powders immobilized in an EPR sample holder. For the purposes of this investigation, all samples were measured at two frequencies: 52.0 GHz and 385 GHz. The temperature dependence was assessed through measurements at 7, 15, 30, and 60 K. Spin Hamiltonian parameters were determined from measurements at the lowest temperature of 7 K to ensure well-coupled effective spin ground states in the cases of **1-Gd** and **3-Gd**. All spectra were simulated using the EasySpin (version 6.0.0-dev.51) toolbox for Matlab.^56^

### Cyclic Voltammetry

Cyclic voltammetry experiments were conducted in an argon-filled glovebox using a PGSTAT204 potentiostat from Metrohm. A three-electrode setup involving a glassy carbon working electrode, a platinum spring counter electrode and a silver wire reference electrode was used. All measurements were conducted cycling the solvent range 4-fold at a 100 mV/s scan rate and *E*_1/2_ averaged, while all voltammograms displayed in the main text constitute the second scan. Due to chemical incompatibility of the organometallic complexes with ferrocene, the measurements were externally referenced to ferrocene solutions with identical supporting electrolyte concentrations and electrode setup. Compounds **1-Dy** and **2-Dy** were measured as DCM solutions, and **3-Dy** was measured as a THF solution. For all measurements, 220 mmol/L electrolyte concentration ([^*n*^Bu_4_N]­[PF_6_]) was used. Sample concentrations of 3 mmol/L were used for all compounds.

### Elemental Analysis

Elemental analysis was carried out with a PerkinElmer 2400 Series II CHNS/O analyzer with assistance of Dr. Rui Huang. The crystalline compounds (∼1–3 mg) were weighed into tin sample holders and folded multiple times to ensure proper sealing from the surrounding atmosphere. The samples were then transferred to the instrument in an airtight container.

### Magnetometry

Magnetic susceptibility data were collected on a Quantum Design MPMS3 Superconducting Quantum Interference Device (SQUID) magnetometer. The magnetic samples of [(Cp*_2_Ln)_2_(μ-flv^•^)]­[Al­(OC­{CF_3_}_3_)_4_] (**1-Ln**), [(Cp*_2_Ln)_2_(μ-flv)] (**2-Ln**) and [K­(crypt-222)]­[(Cp*_2_Ln)_2_(μ-flv^•^)] (**3-Ln**) were prepared by saturating and covering dried, crushed crystalline solids (**1-Gd**: 13.9 mg, **1-Dy**: 18.3 mg, **2-Gd**: 21.8 mg, **2-Dy**: 18.2 mg, **3-Gd**: 20.9 mg, **3-Dy**: 24.4 mg) with molten eicosane (**1-Gd**: 29.8 mg; **1-Dy**: 43.0 mg, **2-Gd**: 43.8 mg, **2-Dy**: 31.8 mg, **3-Gd**: 50.0 mg, **3-Dy**: 59.6 mg) at 60 °C (**1-Ln** and **2-L**n) and 50 °C (**3-Ln**), respectively, to prevent crystallite torquing and to provide good thermal contact between the sample and the bath. The samples were sealed airtight and transferred to the magnetometer. The core diamagnetism was estimated using Pascal’s constants.^57^

The fitting procedure of the χ_M_ *T* vs *T* data for **1-Gd** - **3-Gd** is explained in detail under the static magnetic susceptibility subsection in the Results and Discussion section. Essentially two types of fits to the data were carried out in the temperature ranges of 2 to 300 K and 25 to 300 K. The resulting fit parameters agreed better to one another between 0.1 and 1.0 T data for fits to data from 25 to 300 K for each compound (Figures S10–S12) and, hence, are those discussed in the main text. The summarized fitting results for both temperature regimes are listed in Table S6.

Ac relaxation data were fit using the CCFIT2 program.^58^ Dc relaxation data were fit to a stretched exponential according to eq 1 using the Origin 9.0.0 b45 software^59^

1 $$M\left(t\right)=M_{\mathrm{eq}}+\left(M_{0}-M_{\mathrm{eq}}\right)\exp\left(-{\left(\frac{t}{\tau }\right)}^{\beta }\right)$$

where *M*_0_ is the initial magnetization, *M*_eq_ is the last fit point (obtained via multiplying *M*_0_ by 0.01), β the stretch factor and τ the relaxation time.

### Density Functional Theory

Unrestricted density functional theory (DFT) calculations were carried out for **1-Gd**, **2-Gd**, and **3-Gd** using ORCA 5.0.4 software.^60,61^ Crystal coordinates of all three complexes were optimized with the TPSSh functional at the def2-TZVP theory level.^62,63^ Frequency calculations conducted on optimized coordinates confirmed the presence of an energetic minimum via the absence of imaginary frequencies. The predicted frequency modes were printed using the Multiwfn program applying a Lorentzian line broadening with an 8 cm^–1^ full width at half-maximum (FWHM).^64,65^ The calculated IR frequencies were shifted by 150, 140, and 145 cm^–1^, respectively, to better align with features of the experimental spectra obtained for **1-Gd**, **2-Gd,** and **3-Gd**. Applying an energy shift of this sort is common practice, and addresses the deviation between experimental and computational spectra as DFT calculations constitute approximations.^66,67^ Electronic absorption transitions in the UV and visible regions were predicted through time-dependent DFT (TD-DFT) calculations on the optimized coordinates with the B3LYP functional employing the zeroth-order regular approximation (ZORA) approach for relativistic treatment with CPCM solvent models.^68–74^ The ZORA-def2-TZVP basis set was used for all atoms and ZORA-def2-TZVPP basis set was used for the treatment of atoms constituting flv.^75^ The segmented all-electron relativistically contracted (SARC) basis set with quadruple-ζ quality SARC2-DKH-QZVP was applied for the treatment of Gd.^76^ Spectra were generated from the predicted TD-DFT transitions using Multiwfn with a Gaussian broadening and a FWHM of 0.67 eV. The predicted transitions were energetically shifted by 0.25, 0.37, and 0.75 eV for **1-Gd**, **2-Gd**, and **3-Gd**, respectively. For the determination of magnetic exchange coupling constants, broken-symmetry DFT calculations were employed on unoptimized crystal coordinates. EPR parameters (*g* factor, *D*, *E*/*D*) were calculated using the EPRNMR block of ORCA on the broken-symmetry generated orbitals. All calculations employed the SARC/J auxiliary basis as well as reformulated Grimme’s D3 dispersion correction with Becke-Johnson damping (D3BJ).^77,78^ The generation of the spin density and molecular orbital distributions was accomplished employing the orca_plot module and the VMD program was used for orbital visualizations.^79^

#### Synthesis

##### [(Cp*_2_Gd)_2_(μ-flv^•^)]­[Al­(OC­{CF_3_}_3_)_4_], **1-Gd**

To a 20 mL vial containing dissolved **2-Gd** (69.6 mg, 0.0640 mmol) in DCM (5 mL), dissolved [Thian^•^]­[Al­(OC­{CF_3_}_3_)_4_] (75.7 mg, 0.0640 mmol) in DCM (3 mL) was added in one portion. An immediate color change from orange to dark green was observed, and the reaction mixture was stirred for 15 min. Subsequently, the stirring reaction mixture was evaporated to dryness under vacuum. The obtained dark yellow and greenish solid was washed three times with toluene (7 mL in total) and the toluene-insoluble solid was dried under vacuum. This dark green solid was redissolved in a minimum amount of DCM, filtered, and stored in the freezer for crystallization. Dark green crystals of **1-Gd** suitable for single-crystal X-ray diffraction (SCXRD) analysis were obtained from a concentrated DCM solution at −35 °C over the course of 4 days. Crystalline yield of **1-Gd**: 66.2 mg (0.0309 mmol, 48%). Anal. Calcd for C_71_H_70_AlF_36_Cl_2_Gd_2_N_4_O_4_ (**1-Gd**·CH_2_Cl_2_): C, 39.86; H, 3.30; N, 2.62; found: C, 39.58; H, 3.24; N, 2.61. IR (ATR, cm^–1^): 2911 (vw), 2865 (vw), 1538 (vw), 1351 (vw), 1295 (w), 1273 (m), 1238 (m), 1213 (vs), 1161 (m), 1144 (vw), 1133 (vw), 971 (vs), 833 (vw), 753 (w), 742 (v), 725 (s).

##### [(Cp*_2_Dy)_2_(μ-flv^•^)]­[Al­(OC­{CF_3_}_3_)_4_], **1-Dy**

To a 20 mL vial containing dissolved **2-Dy** (74.8 mg, 0.0681 mmol) in DCM (7 mL), dissolved [Thian^•^]­[Al­(OC­{CF_3_}_3_)_4_] (81.0 mg, 0.0684 mmol) in DCM (2 mL) was added in one portion. An immediate color change from orange to dark green was observed, and the reaction mixture was stirred for 15 min. Subsequently, the stirring reaction mixture was evaporated to dryness under vacuum. The obtained dark yellow and greenish solid was washed three times with toluene (7 mL in total), and the toluene-insoluble solid was dried under vacuum. This dark green solid was redissolved in a minimum amount of DCM, filtered, and stored in the freezer for crystallization. Dark green crystals of **1-Dy** suitable for single-crystal X-ray diffraction analysis were obtained from a concentrated DCM solution at −35 °C over the course of 4 days. Crystalline yield of **1-Dy**: 110.3 mg (0.0513 mmol, 75%). Anal. Calcd for C_71_H_70_AlF_36_Cl_2_Dy_2_N_4_O_4_ (**1-Dy**·CH_2_Cl_2_): C, 39.66; H, 3.28; N, 2.61; found: C, 39.89; H, 3.23; N, 2.62. IR (ATR, cm^–1^): 2919 (vw), 2866 (vw), 1538 (vw), 1491 (vw), 1452 (vw), 1437 (vw), 1353 (vw), 1297 (m), 1275 (s), 1238 (m), 1213 (vs), 1161 (m), 1144 (w), 1133 (w), 971 (vs), 835 (vw), 753 (m), 742 (vw), 725 (vs).

##### [(Cp*_2_Gd)_2_(μ-flv)], **2-Gd**

To a 20 mL vial containing a stirring solution of Cp*_2_Gd­(BPh_4_) (157.8 mg, 0.2113 mmol) in THF (8 mL), solid K_2_flv (32.8 mg, 0.1057 mmol) was added, resulting in an immediate color change from colorless to orange accompanied by the formation of a colorless solid, presumably KBPh_4_. After stirring at room temperature for 3 h, the mixture was filtered through a Celite plug to give a clear, orange filtrate, which was evaporated to dryness. The resulting orange flaky solid was extracted three times with toluene (∼11 mL), filtered through a Celite plug and evaporated to dryness. The obtained orange solid was then dissolved in 60 °C hot THF (6 mL) and filtered hot through a Celite plug which removed additional colorless insoluble solids. The hot, orange filtrate was cooled to room temperature first, and then to −35 °C in the freezer for crystallization of **2-Gd**. Orange crystals of **2-Gd** were grown at −35 °C over the course of 2 days, suitable for single-crystal X-ray diffraction analysis. **2-Gd** was were obtained as large orange crystals. Crystalline yield of **2-Gd**: 69.6 mg (0.0640 mmol, 61%). Anal. Calcd for C_54_H_68_Gd_2_N_4_ (**2-Gd**): C, 59.63; H, 6.30; N, 5.15; found: C, 59.84; H, 6.39; N, 5.13. IR (ATR, cm^–1^): 2961 (vw), 2937 (w), 2900 (w), 2879 (w), 2857 (w), 1592 (w), 1566 (vw), 1551 (vw), 1530 (vw), 1470 (s), 1452 (vs), 1377 (m), 1295 (s), 1277 (m), 1245 (m), 1236 (m), 1146 (w), 1116 (w), 1033 (w), 1021 (w), 965 (w), 742 (vs).

##### [(Cp*_2_Dy)_2_(μ-flv)], **2-Dy**

To a 20 mL vial containing a stirring solution of Cp*_2_Dy­(BPh_4_) (154.6 mg, 0.2055 mmol) in THF (10 mL), solid K_2_flv (32.0 mg, 0.1031 mmol) was added, resulting in an immediate color change from colorless to orange accompanied by the formation of a colorless solid, presumably KBPh_4_. After stirring at room temperature for 3 h, the mixture was filtered through a Celite plug to give a clear, orange filtrate, which was evaporated to dryness. The resulting orange flaky solid was extracted three times with toluene (12 mL), filtered through a Celite plug and evaporated to dryness. The obtained orange solid was then dissolved in 60 °C hot THF (6 mL) and filtered hot through a Celite plug which removed additional colorless insoluble solids. The hot, orange filtrate was cooled to room temperature first, and then to −35 °C in the freezer for crystallization of **2-Dy**. Orange crystals of **2-Dy** were grown at −35 °C over the course of 2 days, suitable for single-crystal X-ray diffraction analysis. **2-Dy** was obtained as large orange crystals. Crystalline yield of **2-Dy**: 74.8 mg (0.0681 mmol, 66%). Anal. Calcd for C_54_H_68_Dy_2_N_4_ (**2-Dy**): C, 59.06; H, 6.24; N, 5.10; found: C, 58.88; H, 6.16; N, 4.98. IR (ATR, cm^–1^): 2963 (vw), 2902 (w), 2853 (w), 1597 (vw), 1566 (vw), 1470 (m), 1452 (vs), 1435 (vs), 1377 (s), 1297 (s), 1279 (m), 1238 (m), 1148 (w), 1116 (w), 1060 (vw), 1031 (m), 1021 (w), 965 (m), 917 (w), 742 (vs).

##### [K­(crypt-222)]­[(Cp*_2_Gd)_2_(μ-flv^•^)], **3-Gd**

**2-Gd** (102.9 mg, 0.0946 mmol) and crypt-222 (35.5 mg, 0.0943 mmol) were dissolved in THF (7 mL) in a 20 mL vial, and a suspension of KC_8_ (12.8 mg, 0.0947 mmol) in THF (1 mL) was added at once to the orange solution. An immediate color change to intense dark green was observed, which gradually shifted toward dark blue over the course of 5 min. After 30 min of stirring, the reaction mixture was filtered to remove insoluble graphite and the solid in the filter was washed twice with THF (1 mL in total) and the washings were combined with the filtrate. The clear, dark blue filtrate was stored in the freezer at −35 °C for crystallization. Dark-blue crystals of **3-Gd** suitable for single-crystal X-ray diffraction analysis were grown at −35 °C over the course of 4 days. Crystalline yield of **3-Gd**: 97.5 mg (0.0649 mmol, 68%). Anal. Calcd for C_72_H_104_Gd_2_KN_6_O_6_ (**3-Gd**): C, 57.53; H, 6.97; N, 5.59; found: C, 57.39; H, 7.01; N, 5.50. IR (ATR, cm^–1^): 2958 (vw), 2878 (w), 2846 (w), 2766 (vw), 2760 (vw), 2755 (vw), 2721 (vw), 1539 (vw), 1476 (vw), 1457 (m), 1444 (w), 1336 (vs), 1295 (m), 1273 (m), 1260 (m), 1239 (w), 1215 (m), 1178 (m), 1131 (m), 1101 (s), 1087 (m), 1077 (s), 1029 (m), 949 (m), 932 (m), 883 (vw), 829 (vw), 820 (vw), 753 (vw), 732 (m), 719 (m).

##### [K­(crypt-222)]­[(Cp*_2_Dy)_2_(μ-flv^•^)], **3-Dy**

**2-Dy** (100.2 mg, 0.0912 mmol) and crypt-222 (35.6 mg, 0.0946 mmol) were dissolved in THF (8 mL) in a 20 mL vial, and a suspension of KC_8_ (12.5 mg, 0.0925 mmol) in THF (1 mL) was added at once to the orange solution. An immediate color change to intense dark green was observed, which gradually shifted toward dark blue over the course of 5 min. After 30 min of stirring, the reaction mixture was filtered to remove insoluble graphite and the solid in the filter was washed twice with THF (1 mL in total) and the washings were combined with the filtrate. The clear, dark blue filtrate was concentrated and stored in the freezer at −35 °C for crystallization. Dark-blue crystals of **3-Dy** suitable for single-crystal X-ray diffraction analysis were grown at −35 °C over the course of 4 days. Crystalline yield of **3-Dy**: 70.0 mg (0.0462 mmol, 51%). Anal. Calcd for C_72_H_104_Dy_2_KN_6_O_6_ (**3-Dy**): C, 57.13; H, 6.93; N, 5.55; found: C, 57.35; H, 6.72; N, 5.44. Anal. Calcd for C_72_H_104_Dy_2_KN_6_O_6_ (**3-Dy**): C, 57.13; H, 6.93; N, 5.55; found: C, 57.35; H, 6.72; N, 5.44. IR (ATR, cm^–1^): 2956 (vw), 2879 (m), 2848 (m), 2723 (vw), 1541 (vw), 1474 (vw), 1457 (m), 1444 (m), 1336 (vs), 1295 (m), 1275 (s), 1260 (m), 1239 (w), 1217 (m), 1174 (m), 1131 (m), 1100 (vs), 1077 (s), 1031 (m), 949 (s), 939 (s), 932 (s), 885 (w), 829 (w), 822 (w), 753 (w), 721 (s).

## Results and Discussion

### Synthesis and Structural Characterization

The introduction of the fluoflavine (flv) ligand into lanthanide chemistry was pursued via a salt metathesis approach, where the reaction of Cp*_2_Ln­(BPh_4_) with K_2_flv in THF afforded the orange complexes [(Cp*_2_Ln)_2_(μ-flv)] (**2-Ln**) containing a flv^2–^ bridge, under precipitation of KBPh_4_ (Figure 2). **2-Ln** was recrystallized from THF at 60 °C and subsequently stored at −35 °C, yielding orange crystals in 61% (**2-Gd**) and 66% (**2-Dy**) yields. **2-Ln** crystallized in the triclinic space group *P*1̅, where the asymmetric unit reveals symmetry equivalence of the metallocene moieties due to an inversion center residing on the flv ligand. In addition, the asymmetric unit contains one THF molecule (Figure S2). In this series of complexes, the coordination sphere of each lanthanide ion comprises two η^5^-coordinated Cp* rings and two N atoms belonging to the flv ligand. The flv ligands are symmetrically ligated to the Ln ions as shown by the identical Ln–N distances of 2.416(2) Å in **2-Gd** and 2.399(2) Å in **2-Dy** (Tables 1 and S3). The metal ions are slightly out-of-plane displaced relative to the flv ligands, giving rise to a zigzag conformation as expressed by the Ln–N1–N2′–Ln′ angle of 17.5(4)° in **2-Gd** and 16.0(4)° in **2-Dy**. Notably, **2-Ln** is isostructural to the yttrium congener.^10^

**Figure 2:**
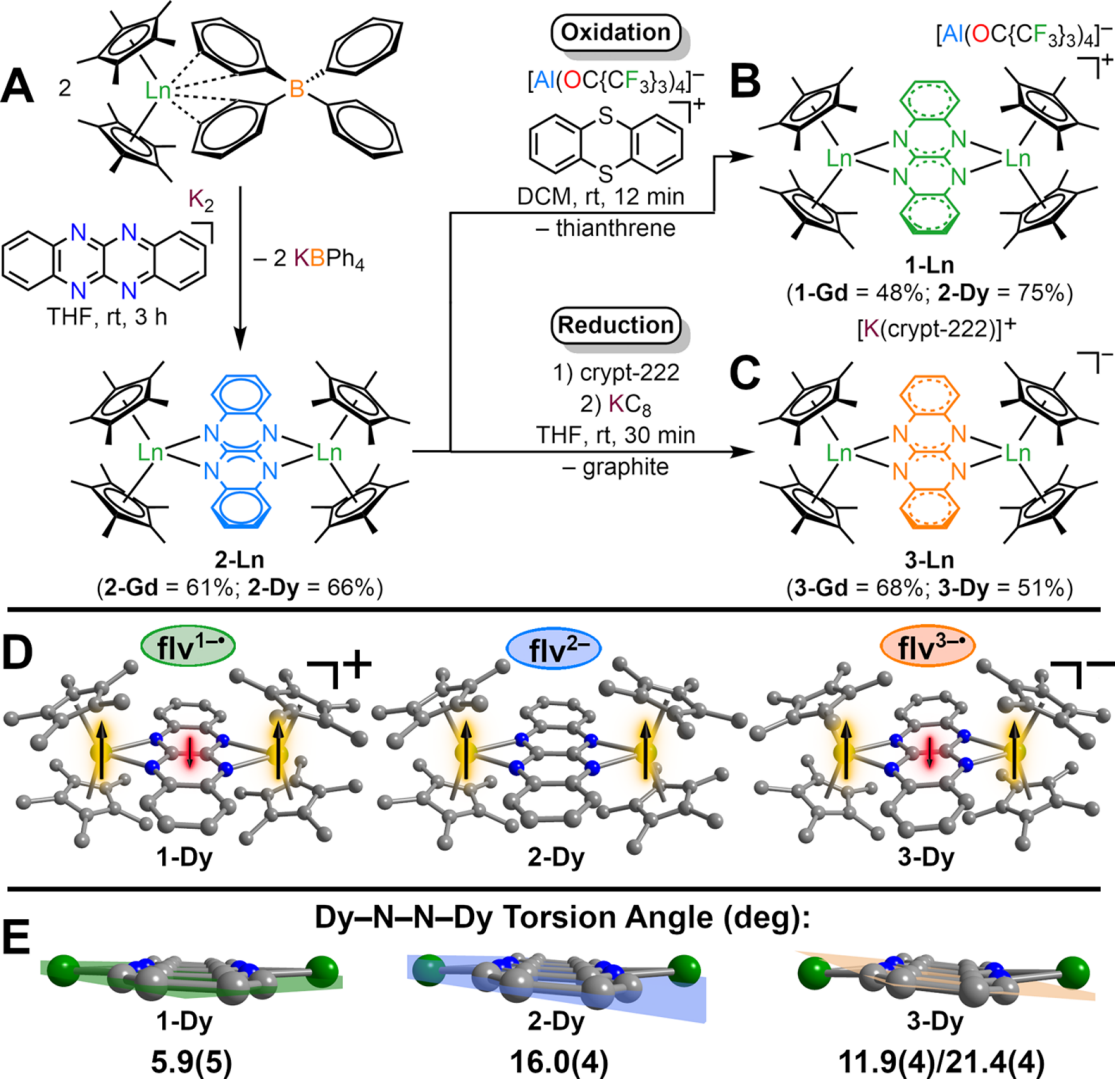
(A) Synthesis of [(Cp*_2_Ln)_2_(μ-flv)] (**2-Ln**) via salt metathesis of Cp*_2_Ln­(BPh_4_) with K_2_flv. (B) Synthesis of [(Cp*_2_Ln)_2_(μ-flv^•^)]­[Al­(OC­{CF_3_}_3_)_4_] (**1-Dy**) through oxidation of [(Cp*_2_Ln)_2_(μ-flv)] (**2-Ln**) with [Thian^•^]­[Al­(OC­{CF_3_}_3_)_4_]. (C) Synthesis of [K­(crypt-222)]­[(Cp*_2_Ln)_2_(μ-flv^•^)] (**3-Ln**) through reduction of **2-Ln** with KC_8_. (D) Structures of the flv^1–•^ bridged complexes **1-Ln** (left), the flv^2–^ bridged complexes **2-Ln** (middle), and flv^3–•^ bridged complexes **3-Ln** (right), all obtained through single-crystal X-ray diffraction analysis. Green, blue, and gray spheres represent lanthanide, nitrogen, and carbon atoms, respectively. All hydrogen atoms, solvent molecules, and the counterions [Al­(OC­{CF_3_}_3_)_4_]^−^ and [K­(crypt-222)]^+^ for **1-Ln** and **3-Ln** are omitted for clarity. (**E**) Enlargement of the Dy–flv–Dy moieties, signifying increased tilting of the flv ligand as a function of a higher negative charge traversing from **1-Dy** to **3-Dy**. Planes are plotted through the nitrogen atoms of the flv ligands.

**Table 1:** Selected Interatomic Distances (Å) and Angles (deg) in **1-Ln–3-Ln**

	**1-Gd**	**1-Dy**	**2-Gd**	**2-Dy**	**3-Gd**	**3-Dy**
Ln–N (avg.)	2.471(3)	2.446(3)	2.416(2)	2.399(2)	2.348(3)	2.321(2)
C–C (central)	1.430(7)	1.426(6)	1.454(5)	1.454(5)	1.387(7)	1.377(5)
Ln···Ln	7.226(1)	7.167(1)	7.105(1)	7.052(1)	6.950(1)	6.899(1)
Cnt–Ln–Cnt	142.1	142.3	141.7	140.8	137.9/140.4	138.0/140.2
Ln–N–N′–Ln′	7.1(5)	5.9(5)	17.5(4)	16.0(4)	15.6(6)/25.8(6)	11.9(4)/21.4(4)

Treatment of crystalline **2-Ln** with [Thian^•^]­[Al­(OC­{CF_3_}_3_)_4_] in DCM initiated an oxidation traceable by an immediate color change from orange to dark green to give complexes bearing flv^1–•^ radical monoanions (Figure 2). These were crystallized from concentrated DCM solutions at −35 °C over the course of four days affording dark green crystals of [(Cp*_2_Ln)_2_(μ-flv^•^)]­[Al­(OC­{CF_3_}_3_)_4_] (**1-Ln**) suitable for single-crystal X-ray diffraction analysis in 48% (**1-Gd**) and 75% (**1-Dy**) yields. Both complexes are isostructural and crystallize in the monoclinic space group *I*2/*a*, with the asymmetric unit containing one metallocene moiety ligated to half a flv^1–•^ ligand alongside half a [Al­(OC­{CF_3_}_3_)_4_]^−^ counteranion and half a DCM molecule (Table S3 and Figure S1). In **1-Ln**, the flv^1–•^ ligand is slightly asymmetrically coordinated as indicated by the ranges in Ln–N distances of 2.468(3)-2.474(3) Å (**1-Gd**) and 2.442(3)-2.449(3) Å (**1-Dy**) (Table 1). The change in oxidation state of the flv ligand can be traced to a slight contraction of the central C–C bond by ∼0.02 Å, from 1.454(5) Å (**2-Gd**) to 1.430(7) Å (**1-Gd**) and 1.441(4) Å (**2-Dy**) to 1.426(6) Å (**1-Dy**). This can be attributed to the depopulation of the lowest-lying ligand-based π* orbital upon oxidation of flv^2–^, which exhibits opposing orbital phases on the nitrogen atoms bound to the metal ions (Figure S60).^10^ The lower negative charge of the ligand in **1-Ln** gives rise to a considerable elongation in the Ln–Ln distance by ∼0.12 Å, from 7.105(1) Å (**2-Gd**) to 7.226(1) Å (**1-Gd**) and 7.033(1) Å (**2-Dy**) to 7.167(1) Å (**1-Dy**). This elongation is accompanied by a smaller out-of-plane displacement of the lanthanide ions as seen in the Ln–N1–N2′–Ln′ angles of 7.1(5)° in **1-Gd** and 5.9(5)° in **1-Dy**, respectively. This can be attributed to a reduction in electrostatic metal–ligand interaction.

Structural changes of this kind were also observed in a series of tetrazine-(tz)-bridged dilanthanide complexes with the tz being in the mono- and divalent oxidation states in [(Cp*_2_Ln)_2_(tz^1–•^)­(THF)_2_]­(BPh_4_) (Ln = Y, Gd, Tb, Dy) and [(Cp*_2_Ln)_2_(tz^2–^)­(THF)_2_] (Ln = Y, Gd).^80,81^ Similar to **1-Gd** and **2-Gd**, a considerable contraction in the Gd–Gd distances of 0.34 Å was observed when comparing the tz^1–•^-containing complex (7.132(5) Å) with the tz^2–^-containing complex (6.792(4) Å).

The successful synthesis of the first flv^1–•^ radical-bridged dilanthanide complexes via chemical oxidation inspired us to probe the accessibility of flv^3–•^ containing complexes through chemical reduction of **2-Ln**. Crystalline **2-Ln** was reacted with the strong reductant KC_8_ in the presence of chelating 2.2.2-cryptand (crypt-222) in THF, resulting in an immediate color change from orange to dark blue, to give flv^3–•^ radical-bridged dilanthanide complexes, **3-Ln**. Dark blue single crystals of **3-Ln** suitable for single-crystal X-ray diffraction analysis were obtained from concentrated THF solutions at −35 °C over the course of four days in yields of 68% (**3-Gd**) and 51% (**3-Dy**). **3-Gd** and **3-Dy** are isostructural and crystallized in the triclinic space group *P*1̅, with the asymmetric unit comprising two independent metallocene moieties with each coordinating half of a flv^3–•^ ligand. In addition, a [K­(crypt-222)]^+^ countercation and four THF molecules are present in the asymmetric unit (Tables S4 and S5, and Figure S3). The inversion centers residing on each flv^3–•^ ligand give rise to two crystallographically independent [(Cp*_2_Ln)_2_(μ-flv^•^)]^−^ units with slightly different structural parameters.

The change in flv oxidation state upon reduction of **2-Ln** is accompanied by considerable inner-flv changes: Most noticeably, the central C–C bond is substantially contracted by ∼0.065 Å, in accordance with the population of the lowest lying π* orbital with bonding contributions on the central C atoms (Figure S60).^10^ The Ln–N distances are shortened by ∼0.068 (Gd) and ∼0.065 Å (Dy), in line with the higher negative charge of the bridging flv unit. The increased negative charge of the bridging ligand is also reflected in significantly decreased Ln–Ln distances of ∼0.155 Å (Gd) and ∼0.134 Å (Dy) (Table 1). Remarkably, the out-of-plane displacement of the lanthanide ions is amplified in **3-Ln** compared to **2-Ln** with Ln–N1–N2′–Ln′ angles of 15.6(6)/25.8(6) in **3-Gd** and 11.9(4)/21.4(4)° in **3-Dy.** Furthermore, **3-Ln** is isostructural with the yttrium congener.^10^

Recently, some of us reported on [K­(crypt-222)]­[(Cp*_2_Ln)_2_(μ-Bbim^•^)], where the bridging Bbim^3–•^ (where H_2_Bbim = 2,2′-bisbenzimidazole) radical is a structural isomer of flv^3–•^.^30^ A structural comparison shows that first the decrease in Ln–Ln distance is ∼0.139 Å (Gd) and ∼0.143 Å (Dy) moving from Bbim^2–^ to Bbim^3–•^ complexes and thus, similar in magnitude relative to the flv congener. Second, the Ln–Ln distance is significantly shorter in [(Cp*_2_Ln)_2_(μ-Bbim^•^)]^−^ with 6.100(1) Å (for Ln = Gd) and 6.060 Å (for Ln = Dy) than the analogous distances in **3-Ln** with 6.950(1) Å for **3-Gd** and 6.899(1) Å for **3-Dy**. Third, traversing from a “2,2′-bisimidazole” to a “[4a,8a]-naphthalene” binding pocket where the central coordinating unit is considered only, affords a compressed bite angle for the N–Ln–N binding pocket with ∼59.4(1) and ∼59.9(7)° for the **3-Gd** and **3-Dy** complexes compared to ∼74.8(1) and ∼75.5(7)° for the bisbenzimidazole radical-bridged Gd and Dy complexes. For either bridging ligand, an out-of-plane displacement of the Ln ions relative to the ligand plane is observed, forming a zigzag arrangement. This may be the result of a higher steric congestion imposed by the steeper ring strain of the Ln–N–C–N four-membered ring in **3-Ln** compared to the less acute ring strain of the Ln–N–C–C–N five-membered ring in the Bbim complexes.

### IR and UV–Vis Spectroscopy

IR spectra of all six compounds exhibit strong absorption features centered around ∼3000 cm^–1^, corresponding to C–H stretches and around ∼1500 cm^–1^, aligning with C–C bond stretches. Additional strong absorptions are observed in fingerprint regions. All three Gd species exhibit superimposable vibrational spectra relative to their Dy analogues in their IR spectra (Figures S4–S6). The crystal coordinates of **1-Gd**, **2-Gd**, and **3-Gd** were geometry optimized (section 8 of Supporting Information), and the optimized structures were subjected to analytical frequency calculations which confirm energetic minima through the absence of imaginary frequencies (Figures S4–S6). The calculated IR frequencies were shifted by 150, 140, and 145 cm^–1^, respectively, to better align with features of the experimental spectra obtained for **1-Gd**, **2-Gd,** and **3-Gd**.

UV–vis absorption spectra for **1-Ln**–**3-Ln** were taken from 220 to 1000 nm in DCM solutions (for **1-Ln**) and THF solutions (for **2-Ln** and **3-Ln**) (see methodology section for concentrations). **1-Ln** and **3-Ln** feature absorptions in the whole experimental range. By contrast, **2-Ln** exhibits absorptions primarily in the UV region and several strong absorption peaks around 500 nm which is congruent with the bright orange color of the solutions (Figure 3). **1-Dy** and **1-Gd** feature a pronounced absorption peak at around 470 nm mainly arising from a π→π* transition which occurs from a flv-based π MO to a virtual orbital consisting of Cp* orbitals. The second distinctive transition predicted by TD-DFT resides at 417 nm, corresponding to an excitation from a Cp*-based π orbital to a Cp*-based σ orbital.

**Figure 3:**
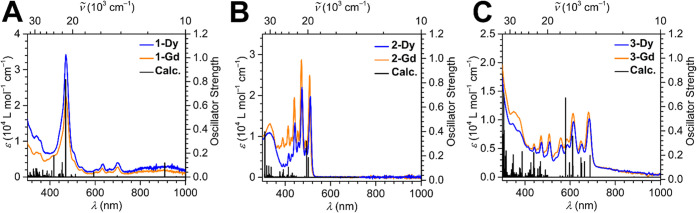
UV–vis absorption spectra of (A) **1-Ln**, (B) **2-Ln**, and (C) **3-Ln**. The experimental spectra for the Dy and Gd complexes are shown as blue and orange lines, respectively. The TD-DFT transitions were calculated for the Gd complexes and are depicted as black vertical lines.

The most intense transition for **2-Gd** has major contributions from an excitation centered at 311 nm originating from a σ MO, which is distributed on the flv and all four Cp* ligands, to a σ orbital situated primarily on flv with small contributions from the Gd ions. The transition positioned at 494 nm is the next largest with contributions from three excitations: HOMO (highest occupied molecular orbital) to Gd-centered LUMO+1, ligand-to-ligand charge transfer from flv-based π orbitals to flv-based π* orbitals, and HOMO to LUMO (lowest unoccupied molecular orbital). For **3-Gd**, multiple prominent transitions are monitored in the visible region, albeit the most intense transition emerges in the UV region at 304 nm. The second strongest transition is in the visible region at around 580 nm due to excitations from a σ-type MO on flv to a π-type MO on flv. The transition at 611 nm is the second most prominent in the visible region. This stems from flv-to-flv excitations and, HOMO to Gd and ligand-orbital excitations. Tables S11–S13 depict the MOs participating in these transitions.

A scrutiny of the frontier molecular orbitals uncovers the electronic structure of each compound, Figure S60. The SOMO and the LUMO of **1-Gd** topologically closely resemble the HOMO and the LUMO of **2-Gd**. The assigned oxidation states support this interpretation since the highest occupied orbital of **1-Gd** is a singly occupied molecular orbital (SOMO) which emerges from the removal of one electron from the HOMO of **2-Gd**. Both the HOMO and the LUMO are flv-based and show π-bonding and π-antibonding character, respectively. The highest occupied orbital of **3-Gd** is a SOMO that is topologically similar to the distribution of the LUMOs of **1-Gd** and **2-Gd**, since it arises from the addition of an electron to the LUMO of **2-Gd**. The LUMO of **3-Gd** primarily exhibits a Gd-centered *d*_*z*_^2^ distribution. This assignment is in line with the observation that no further ligand-based reductions can be chemically achieved for this system. Overall, the increase monitored in the energies of the orbitals from **1-Gd** to **3-Gd** is explained by the rising charge magnitude of flv owing to the addition of extra electrons.

### Cyclic Voltammetry

Recently, a nuanced approach to describe magnetic exchange coupling in radical-bridged SMMs via density functional theory (DFT) was employed,^82,83^ where the complex interplay of competing magnetic interaction pathways is analyzed via the Hubbard Model (HM). The latter was introduced to discuss direct coupling in specific cases of radical-bridged SMMs before,^83,84^ and exploits two parameters to tune the magnetic exchange interaction: First, the transfer integral, *t*, as a measure of electron mobility between two reference sites, e.g., the metal ion and the organic radical ligand, and reflects the stabilization of the bonding orbital due to overlap between SOMOs. Second, the electron repulsion, *U*, as a measure for the energy required to pair two electrons on a singular site. Thus, as per the Hubbard model, the total exchange coupling corresponds to the sum of multiple contributions, eq 2,

2 $$J_{A-B}=J_{0}^{A-B}+{\Delta J}_{\mathrm{KE}}^{A-B}+{\Delta J}_{\mathrm{SP}}^{A-B}$$

with contributions of *J*_0_^A–B^ representing the direct exchange, Δ*J*_KE_^A–B^ the kinetic exchange, and Δ*J*_SP_^A–B^ the spin polarization. The above-mentioned *t* and *U* parameters enter Δ*J*_KE_^A–B^, which is estimated to be the predominant contribution to *J*_A–B_, whereas all other contributions are expected to be significantly smaller.^85^ The most dominant kinetic exchange interaction may be calculated via eq 3,

$$\Delta J_{\mathrm{KE}}^{A-B}=-t^{2}/U-t^{\prime 2}/U^{\prime }$$ 3

where ^*t*^/_*U*_ denote Hubbard parameters of one site, and *t*′/_*U*′_ denote Hubbard parameters of a second site.

In theory, the magnetic exchange coupling can be amplified by either raising *t* through enhanced metal–ligand orbital overlap, or decreasing *U* by lowering the radical’s reduction potential to match the potential of the Ln^III^/Ln^II^ redox couple.^84^ The reduction potentials for the lanthanide redox couples utilized in this study were approximated to be −2.8 V for Gd^III^/Gd^II^ and −2.5 V for Dy^III^/Dy^II^, respectively.^42,43^ A smaller difference was quantified for organometallic Cp′_3_Ln complexes (where Cp′ = C_5_H_4_SiMe_3_) in THF solutions with Gd^III^/Gd^II^ and Dy^III^/Dy^II^ potentials measured at −2.98 V and −2.96 V, respectively.^86^

Consequently, **1-Dy**–**3-Dy** were studied via cyclic voltammetry, where **1-Dy** and **2-Dy** were measured in DCM, and **3-Dy** in THF solutions (Figures S7–S9). The differing solvent use was required as **1-Dy** and **3-Dy** are not stable in THF and DCM, respectively.

For **2-Dy**, a single quasi-reversible redox event is observed at −0.122(4) V vs Fc^+^/Fc (Figure S8), which is assigned to the flv^1–•^/flv^2–^ redox couple. Moreover, this potential coincides with the respective potential of −0.095(7) V for the yttrium congener.^10^

The cyclic voltammogram of **1-Dy** suggests richer redox behavior: Upon scanning from −2.5 V toward positive potentials, a quasi-reversible feature is found at −1.422(3) V, which is ascribed to the flv^1–•^/flv^2–^ redox process (Figure S7). This corresponds to a considerable anodic shift by ∼1.3 V relative to **2-Dy**. An irreversible oxidation (at ∼ −0.93 V) upon anodic scanning appeared in the probed potential range. In addition, reduction features (at ∼−1.07 V, −2.14 V, and −3.06 V) upon cathodic scanning indicate electrochemical instability of **1-Dy**. Similar processes were observed for the yttrium congener.^10^

For **3-Dy**, the cyclic voltammogram shows a quasi-reversible redox process at −1.898(4) V, which is ascribed to the flv^2–^/flv^3–•^ process (Figure S9). No additional features were apparent in the probed potential range. This is in excellent agreement with the voltammogram of the yttrium congener.^10^

For the radical-bridged complexes **1-Dy** and **3-Dy**, the higher negative charge of the bridging flv ligand correlates with the increasingly negative potentials for the respective flv^1–•^/flv^2–^ and flv^2–^/flv^3–•^ processes. This hints at a reduction in *U* and thus, entails strengthened magnetic coupling between the paramagnetic lanthanide ions and the organic radical ligands. This complements the electronic structure calculations on the yttrium congeners suggesting that the successive electron uptake marginally impacts covalency and aromaticity, whereas both the spin density and the spin polarization between α- and β-spin manifolds are majorly affected.^10^ In sum, these findings point at a strengthening of the magnetic exchange coupling as a function of a negative charge increase on the bridging flv ligand.

### Static Magnetic Susceptibility

The magnetic exchange coupling in **1-Ln** and **3-Ln** was first probed through static magnetic susceptibility measurements on polycrystalline samples between 2 and 300 K under a 0.1 and 1.0 T direct current (dc) field (Figures 4 and S10–S18). The following discussion is focused on the interpretation of the data obtained under a 0.1 T field. The room temperature χ_M_ *T* values of 16.28 cm^3^K/mol for **1-Gd**, 29.09 cm^3^K/mol for **1-Dy**, 16.43 cm^3^K/mol for **3-Gd** and 28.94 cm^3^K/mol **3-Dy** are in excellent agreement with the expected values for two noninteracting Ln^III^ ions and one *S* = ^1^/_2_ organic radical spin center (16.13 cm^3^K/mol (Gd), 28.71 cm^3^K/mol (Dy), *S* = ^7^/_2_ and *g* = 2.00 for Gd^III^, *S* = ^5^/_2_, *J* = ^15^/_2_ and *g*_*J*_ = ^4^/_3_ for Dy^III^).

**Figure 4:**
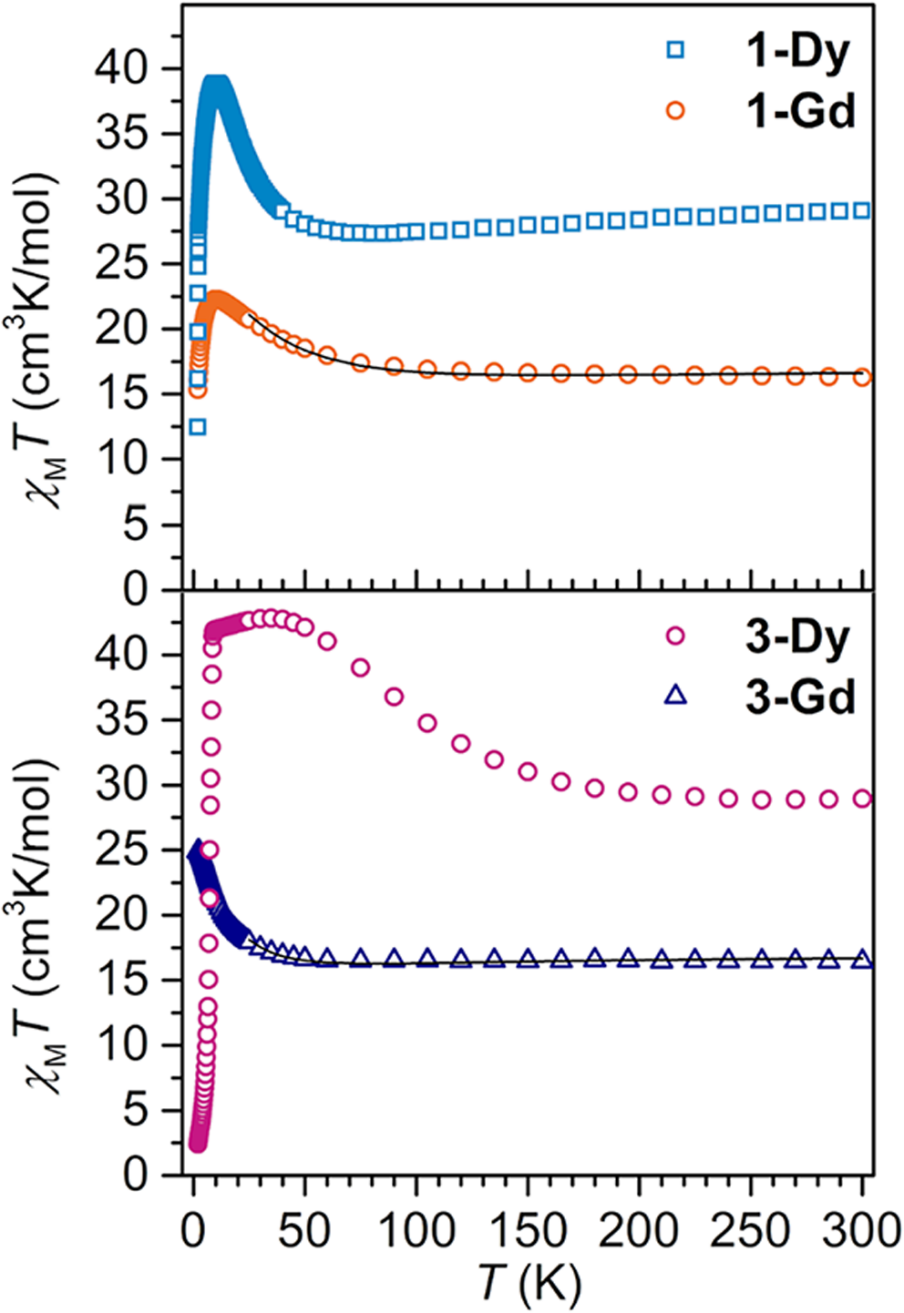
Variable-temperature dc magnetic susceptibility data for restrained polycrystalline samples of **1-Dy** (blue squares), **1-Gd** (orange circles), **3-Dy** (purple circles), and **3-Gd** (dark blue triangles) collected under a 0.1 T applied dc field. Black lines represent fits of the Gd data to a Heisenberg Hamiltonian, yielding *J*_Gd–Rad_ = – 10.5(2) cm^–1^ (**1-Gd**) and *J*_Gd–Rad_ = – 5.3(1) cm^–1^ (**3-Gd**) and *g*_Gd_ = 2.068(2) (**1-Gd**) and *g*_Gd_ = 2.056(2) (**3-Gd**), respectively. 1.0 T data are displayed in Figures S10–S14 and S16.

There are both distinct differences and common features in the dc magnetic susceptibility data for **1-Dy** and **3-Dy** which will be highlighted below. A common feature is that as the temperature is lowered, both **1-Dy** and **3-Dy** exhibit maxima in χ_M_ *T* indicative of a high-angular momentum ground state originating from strong antiferromagnetic coupling between the Dy^III^ ions and the organic radical ligand (Figures 4, S13, and S16).

Specifically, reducing the temperature from 300 K for **1-Dy** bearing the flv^1–•^ radical, causes initially a gradual decline in χ_M_ *T*, reaching a minimum value of 27.32 cm^3^K/mol at 80 K followed by a rise in χ_M_ *T* to a maximum value of 38.87 cm^3^K/mol at 10 K. In general, this progression, in particular the substantial rise in χ_M_ *T* value hints at the presence of a high-angular momentum ground state arising from antiferromagnetic coupling between the lanthanide ions and the radical spin center. Below 10 K, χ_M_ *T* plummets to a value of 12.46 cm^3^K/mol and serves as an indication of magnetic blocking. This type of steep drop in χ_M_ *T* was observed for other radical-bridged SMMs.^25,19^ In accord with this interpretation, field- and zero-field-cooled (fc/zfc) magnetic susceptibility data reveal a distinct divergence at 2.4 K (Figure 5).

**Figure 5:**
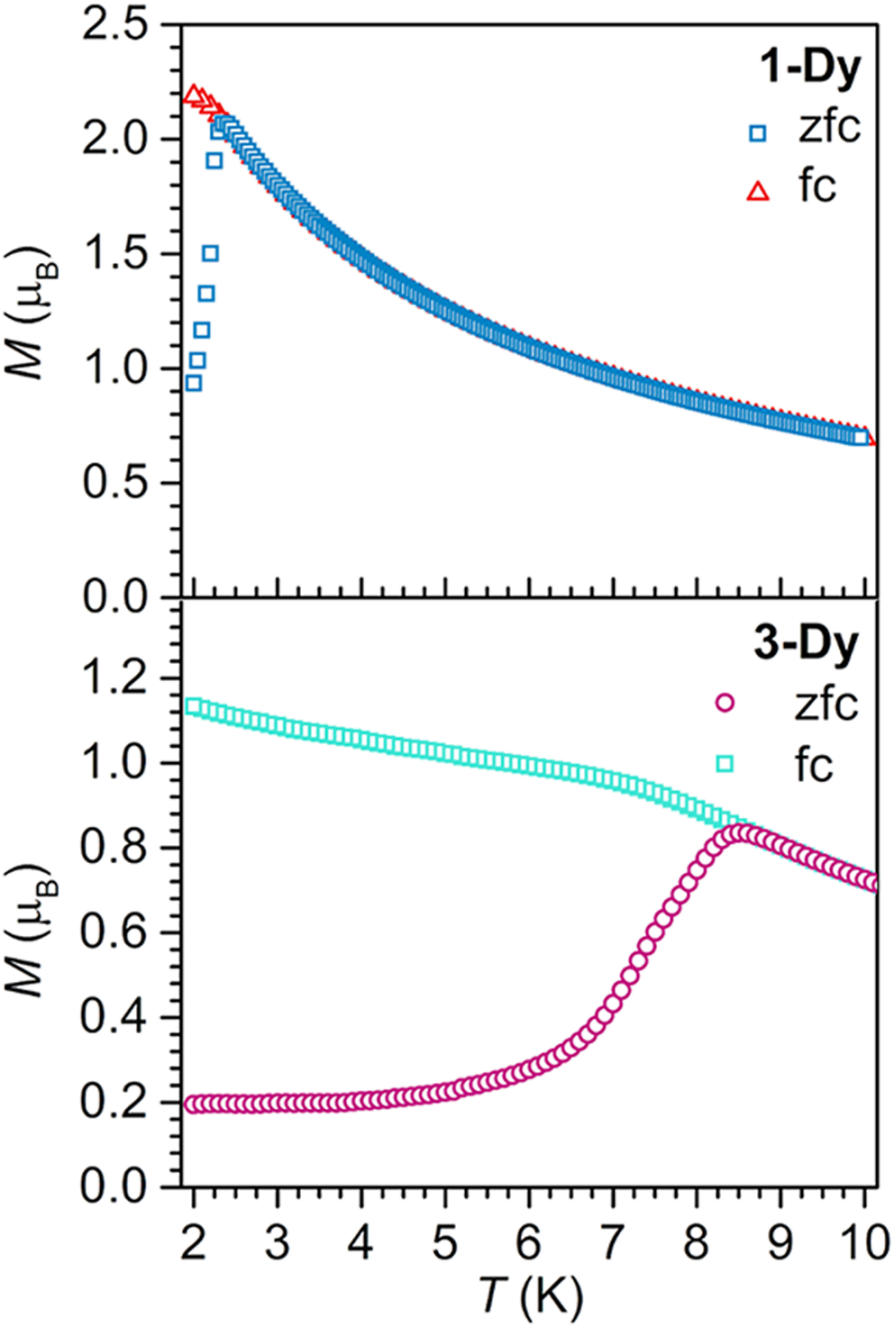
Plot of magnetization vs temperature for **1-Dy** (top) and **3-Dy** (bottom) during field-cooled (**1-Dy**: red triangles, **3-Dy**: turquoise squares) and zero-field-cooled (**1-Dy**: blue squares, **3-Dy**: purple circles) measurements under a 0.1 T applied dc field displaying the thermoremanent magnetization.

By contrast, with declining temperatures from 300 K for **3-Dy** comprising the flv^3–•^ radical, χ_M_ *T* gradually increases to a maximum value of 42.83 cm^3^K/mol at 35 K without passing through a shallow minimum (Figure S16). Subsequently, χ_M_ *T* slowly declines to 41.77 cm^3^K/mol at 9 K, before precipitously dropping to 2.41 cm^3^K/mol at 2 K. The field- and zero-field-cooled (fc/zfc) magnetic susceptibility data features a divergence at 8.5 K (Figure 5). This is near the temperature at which χ_M_ *T* drops, and represents a 3.3-fold increase at which divergence occurs in the collected fc/zfc magnetic susceptibility data compared to **1**-**Dy**.

For **1-Gd**, as the temperature is lowered, the increase of χ_M_ *T* is more gradual (Figures 4 and S10), and reaches a maximum value of 22.27 cm^3^K/mol at 10 K, before dropping to 15.35 cm^3^K/mol at 2 K. The orbital singlet of Gd^III^ is not complicated by the effects of spin–orbit coupling facilitating the extraction of magnetic exchange coupling strength by fitting the experimental χ_M_ *T* vs *T* curves to a Heisenberg-Dirac-van-Vleck (HDvV) Hamiltonian according to eq 4, as implemented in the PHI program^83,87^

$$\begin{array}{l} {Ĥ}_{\mathrm{HDvV}}=-2J_{\mathrm{Gd}-\mathrm{Rad}}{Ŝ}_{\mathrm{Rad}}\cdot \left({Ŝ}_{\mathrm{Gd}1}+{Ŝ}_{\mathrm{Gd}2}\right)\\ +\mu_{B}g\left({Ŝ}_{\mathrm{Gd}1}+{Ŝ}_{\mathrm{Gd}2}+{Ŝ}_{\mathrm{Rad}}\right)\cdot \mathrm{B⃗} \end{array}$$ 4

where *Ŝ* are the local spin operators for magnetic centers Rad (radical), Gd1 and Gd2. *J*_Gd–Rad_ describes the magnetic exchange coupling constant between the respective Gd^III^ and Rad sites (identical for Gd1–Rad and Gd2–Rad).

The χ_M_ *T* vs *T* curves for **1-Gd** were fitted to eq 4 and yielded strong antiferromagnetic coupling *J*_Gd–Rad_ of −10.5(2) cm^–1^ placing it among the highest known for organic radical-bridged dinuclear Gd^III^ complexes. For comparison, *J*_Gd–Rad_ of **1-Gd** exceeds the −7.2 cm^–1^ observed for [(Cp*_2_Gd)_2_(μ-tz^•^)­(THF)_2_]­(BPh_4_) (tz: 1,2,4,5-tetrazine) and the −10 cm^–1^ determined for [(Cp*_2_Gd)_2_(μ-bpym^•^)]­(BPh_4_) (bpym: 2,2′-bipyrimidine).^29,80^

A similar yet less pronounced trend is found for **3-Gd**: With lowering temperatures, χ_M_ *T* first increases slightly to a value of 16.55 cm^3^K/mol at 180 K, then declines to a minimum of 16.50 cm^3^K/mol at 75 K, before rising to a maximum of 24.89 cm^3^K/mol at 2 K (Figures 4 and S12). A drop at low temperatures akin to the χ_M_ *T* behavior for **1-Gd** is not observed.

Fitting the χ_M_ *T* data at 0.1 T for **3-Gd** using eq 4 gave a magnetic exchange coupling constant *J*_Gd–Rad_ of −5.3(1) cm^–1^, which is approximately half as high in value relative to **1-Gd**. This is somewhat unexpected as a higher charge of the radical bridge elicits more diffuse spin orbitals, which should increase orbital overlap with the 4f orbitals of the Ln^III^ ions, thereby maximizing magnetic exchange between the two units. In addition, we previously predicted a substantially augmented spin density transfer from the flv^3–•^ radical to the Y^III^ metal centers compared to flv^1–•^.^10^

The majority of known radical-bridged dilanthanide complexes employed monoanionic radical ligands, and thus engendered cationic complexes such as the mentioned examples above and compounds bearing substituted bipyrimidine radicals of the type [(Cp*_2_Ln)_2_(μ-5,5′-R_2_bpym^•^)]­(BPh_4_) (R = NMe_2_, OEt, Me, F), respectively.^26^ So far only one study contrasts the magnetic properties of lanthanide complexes containing an organic radical bridge in two differing oxidation states, namely [(Cp*_2_Ln)_2_(μ-tppz^•^)]­(BPh_4_) and [K­(crypt-222)]­[(Cp*_2_Ln)_2_(μ-tppz^•^)] (Ln: Gd, Tb, Dy) comprising the 2,3,5,6-tetra­(2-pyridyl)­pyrazine ligand, abbreviated as tppz.^40^ Compared to **1-Ln** and **3-Ln**, the tppz-bridged complexes exhibit a similar, yet less pronounced trend: The *J*_Gd–Rad_ for the cationic Gd^III^ complex with a μ-tppz^1–•^ radical-bridge was determined to be −6.91(4) cm^–1^, whereas the coupling strength for the corresponding anionic complex bearing a tppz^3–•^radical was found to be slightly weaker with a *J*_Gd–Rad_ of –6.29(3) cm^–1^. Notably, only [(Cp*_2_Dy)_2_(μ-tppz^•^)]­(BPh_4_) featured slow magnetic relaxation and open magnetic hysteresis loops below 3.25 K.^40^

The magnetic coupling of **3-Gd** can be further compared to [K­(crypt-222)]­[(Cp*_2_Gd)_2_(μ-Bbim^•^)] bearing the trianionic 2,2′-bisbenzimidazole radical, Bbim^3–•^.^30^ The structural similarity of the trianionic bridging ligands (flv^3–•^ vs Bbim^3–•^) in akin organolanthanide complexes allows a study of the impact of the coordinating pocket onto the magnetic coupling: the N–Gd–N angle, defined as the bite angle, is for Bbim with 74.3(1)/75.3(1)° much larger relative to 59.7(1)/60.1(1)° found in **3-Gd**. This results in a 0.85 Å shorter Gd–Gd distance in the Bbim^3–•^-containing complex versus the flv^3–•^-containing complex, **3-Gd**, giving a weaker exchange coupling constant *J*_Gd–Rad_ of −1.96(2) cm^–1^, which is more than 50% smaller in value compared to **3-Gd**.

For completeness, dc magnetic susceptibility measurements were also taken on polycrystalline samples of **2-Gd** and **2-Dy**, each containing the diamagnetic flv^2–^ bridge, under 0.1 and 1.0 T fields (Figures S11, S14 and S15). In the following, the data collected under 0.1 T is discussed. The room temperature χ_M_ *T* values of 16.00 cm^3^K/mol and 28.67 cm^3^K/mol are in agreement with the expected values of 15.76 cm^3^K/mol (Gd) and 28.34 cm^3^K/mol (Dy) for two magnetically uncoupled Ln^III^ ions. For **2-Dy**, lowering the temperature causes a gradual decrease in the χ_M_ *T* values to 24.72 cm^3^K/mol at ∼20 K, followed by a faster decline to a final value of 17.74 cm^3^K/mol at 2 K. For **2-Gd**, the χ_M_ *T* curve remains essentially unchanged with decreasing temperatures up until ∼10 K, below which a slight drop to a value of 14.16 cm^3^K/mol at 2 K occurs. For each **2-Ln**, this temperature dependence of χ_M_ *T* may be attributed to thermal depopulation of low-lying excited states and/or antiferromagnetic coupling. Fitting the χ_M_ *T* vs *T* data for **2-Gd** afforded a *J*_Gd–Gd_ value of –0.005(2) cm^–1^, essentially confirming the uncoupled nature of the lanthanide ions in the nonradical-bridged complexes. Relative to **1-Dy** and **3-Dy**, on the timescale of the dc magnetic susceptibility measurements, **2-Dy** lacks magnetic blocking features (Figure S18).

### Dynamic Magnetic Susceptibility

The static magnetic susceptibility data of **1-Dy** and **3-Dy** exhibit features reminiscent of magnetic blocking at low temperatures. To gain insight into the magnetization dynamics, variable-temperature, variable-frequency alternating current (ac) magnetic susceptibility measurements were carried out. Both complexes exhibit fully temperature-dependent peaks in the out-of-phase (χ_M_″) ac magnetic susceptibility under zero applied dc field, indicative of long magnetic relaxation times (Table S7). The results of the magnetic relaxation analysis are summarized in Table S8.

Subjecting **1-Dy** to an oscillating ac magnetic field of 3 Oe at frequencies between 0.1 and 1000 Hz, a single χ_M_″ peak was monitored from 2.9 to 7.5 K (Figure 6). Similarly for **3-Dy**, broad, fully temperature-dependent peaks were observed between 0.1 and 1000 Hz from 10 to 16 K (Figure 7).

**Figure 6:**
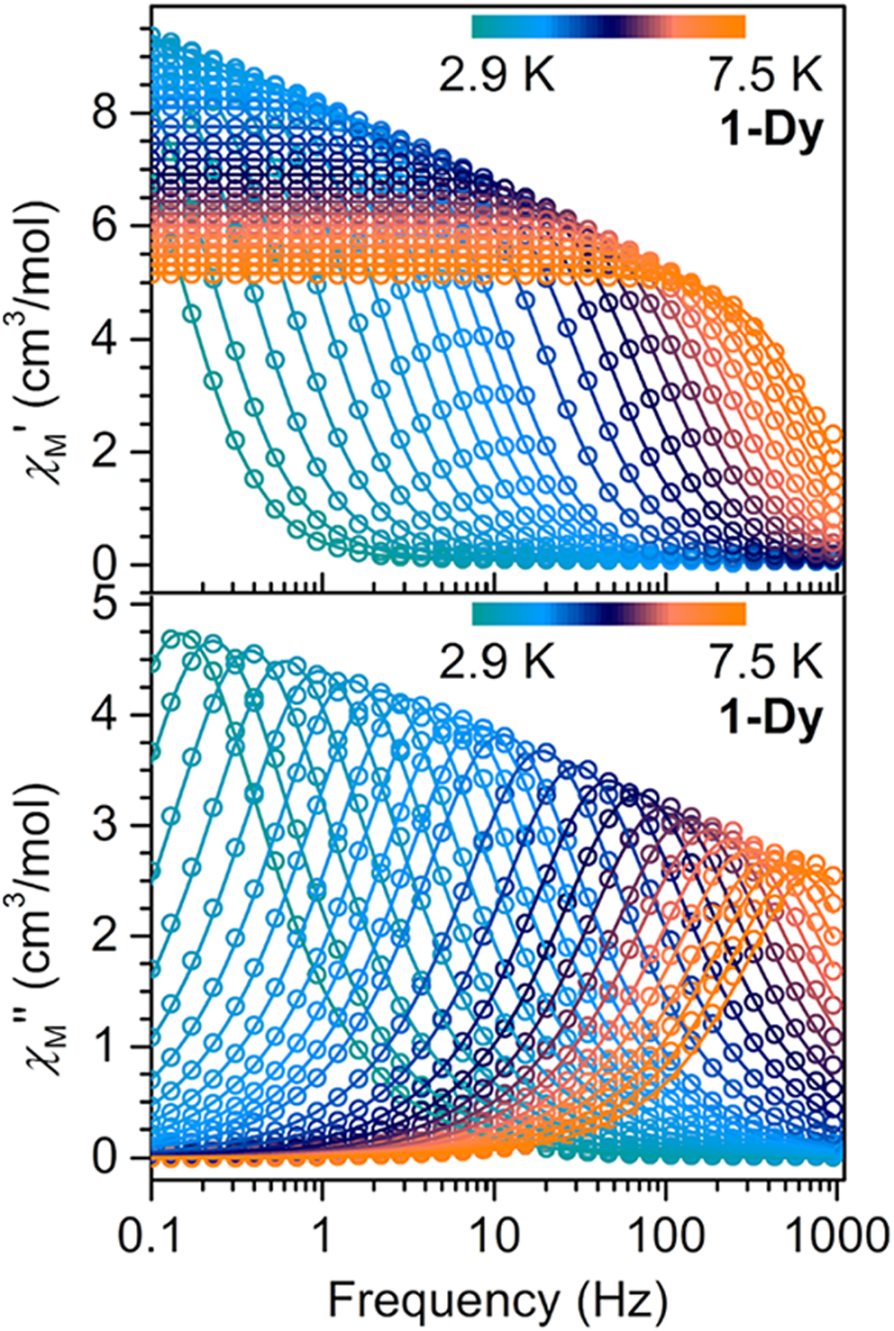
Variable-temperature, variable-frequency in-phase (χ_M_′, top) and out-of-phase (χ_M_″, bottom) ac magnetic susceptibility data collected under zero applied dc field for **1-Dy** from 2.9 to 7.5 K. Solid lines indicate the fits to the generalized Debye model.

**Figure 7:**
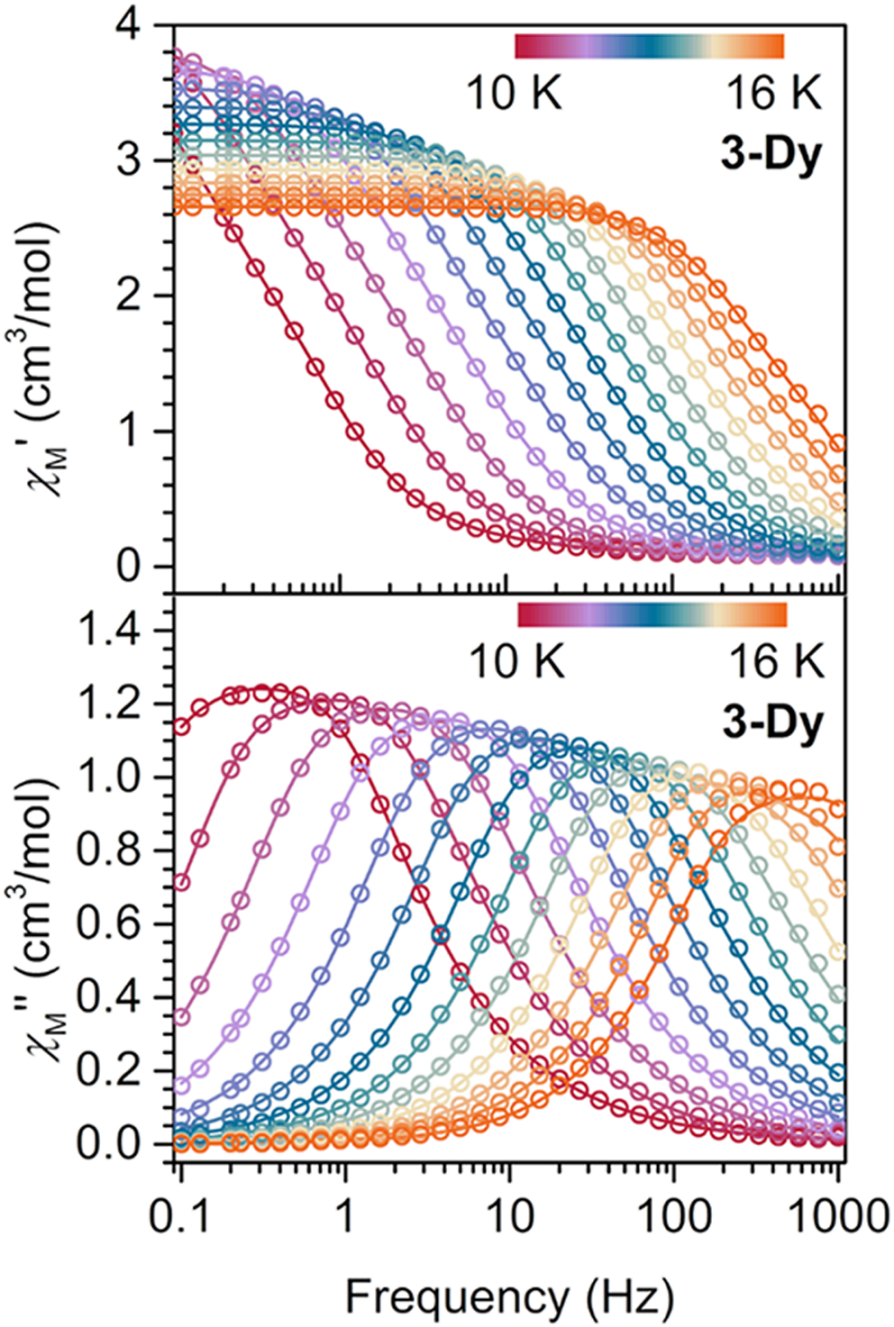
Variable-temperature, variable-frequency in-phase (χ_M_′, top) and out-of-phase (χ_M_″, bottom) ac magnetic susceptibility data collected under zero applied dc field for **3-Dy**, from 10 to 16 K. Solid lines indicate the fits to the double generalized Debye model.

The in-phase (χ_M_′) and out-of-phase (χ_M_″) components of the ac magnetic susceptibility for **1-Dy** were employed to construct Cole–Cole (Argand) plots (χ_M_″ vs χ_M_′), which were in the following fit to a generalized Debye model (Figure S19). All relaxation fitting was performed using the CCFit2 software.^58^ The magnetic relaxation rates were fit according to

5 $$\tau^{-1}=\tau_{\mathrm{QTM}}^{-1}+CT^{n}+\tau_{0}^{-1}\exp\left(-U_{\mathrm{eff}}{\left(k_{B}T\right)}^{-1}\right)$$

Here, the first term reflects the quantum tunneling of the magnetization (QTM) process and the second term is for the Raman relaxation process (∝*T* ^*n*^, *n* usually varies in value but does not exceed 9). The third term models the Orbach process (∝ exp­(*U*_eff_/*k*_B_ *T*)), where *U*_eff_ is the energy barrier to spin-reversal, τ_0_ is the pre-exponential factor and *k*_B_ is the Boltzmann constant. An exponential dependence of the latter typically gives a linear dependence in Arrhenius plots (ln­(τ) vs 1/*T*).^16,88^ As showcased in the following, the successful modeling of the Arrhenius plots did not require each term.

For **1-Dy**, the relaxation times, τ, extracted from ac magnetic susceptibility measurements, are fully temperature-dependent, affording a linear slope in the Arrhenius plot (Figure S37). Thus, fitting the ln­(τ) vs 1/*T* plot to a single Orbach process, yielded *U*_eff_ of 28.36(7) cm^–1^ and τ_0_ of 9.2(2) × 10^–7^ s (Figure S37).

Dc relaxation experiments were conducted to analyze the temperature ranges inaccessible through ac magnetic susceptibility methods due to long relaxation times. In this technique, first, a high dc magnetic field is applied to the sample to reach magnetic saturation. Second, the field is quickly nullified, and third, after reaching zero field, the magnetic relaxation is measured which will follow an exponential dependence.

For **1-Dy**, the dc relaxation experiments took place between 1.8 and 2.5 K. The obtained magnetization decay curves were fit to a stretched exponential function, eq 1 (Figures S23–S27).^89^

Considering the full temperature range for **1-Dy** with relaxation times obtained from ac magnetic susceptibility measurements and dc relaxation experiments, a fit to a single Orbach process gave *U*_eff_ = 26.9(2) cm^–1^ and τ_0_ = 1.5(2) × 10^–6^ s but exhibited a deviation from linearity in the low-temperature regime (Figures 8 and S38). The inclusion of a Raman process in addition to the thermally activated regime, resulted in a near-unchanged Orbach process (*U*_eff_ = 28.4(1) cm^–1^, τ_0_ = 9.6(4) × 10^–7^ s) with an improved fit of the low temperature data with Raman parameters of 2.3(12) × 10^–6^ s^–1^K^–*n*^ for *C* and 9.0(8) for *n* (Figure S39). Fixing the Orbach process to the values obtained via ac magnetometry yields superior reproduction of the experimental data with Raman parameters of 5.5(34) × 10^–6^ s^–1^K^–*n*^ for *C* and 7.6(9) for *n* (Figure S40).

**Figure 8:**
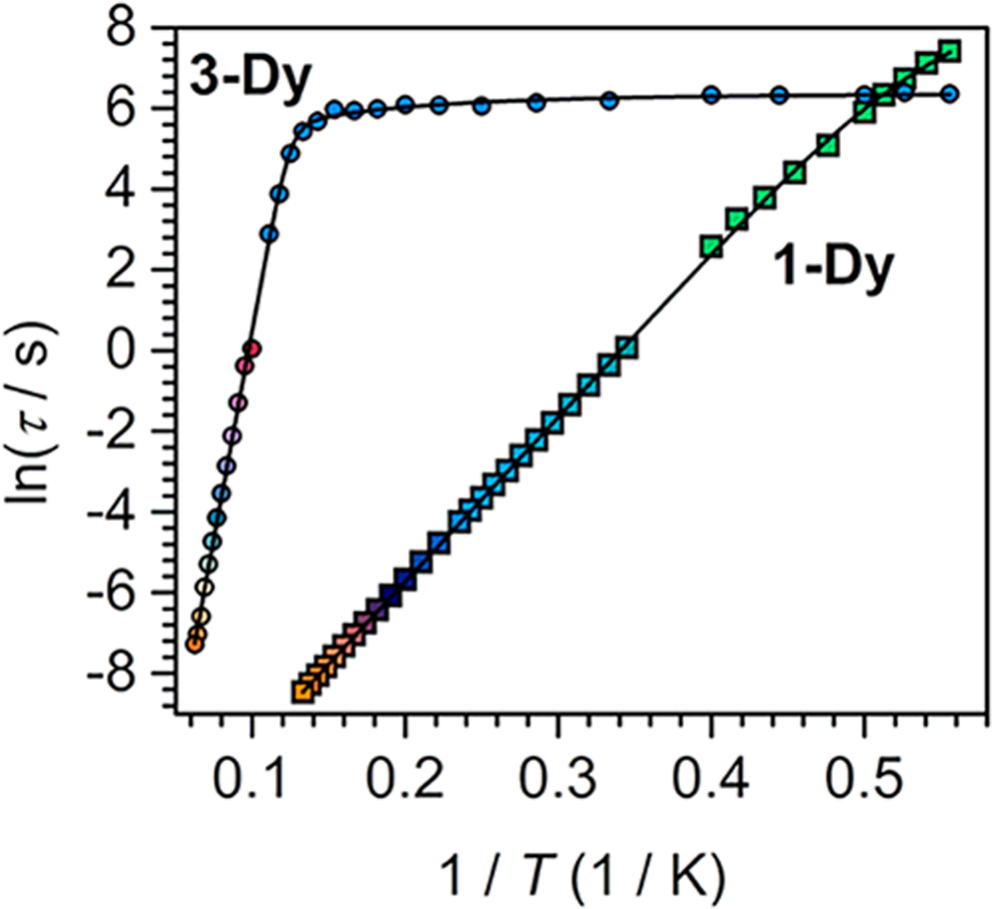
Plot of natural log of the relaxation time versus the inverse temperature for **1-Dy** (temperature range 1.8 to 7.5 K) and for **3-Dy** (temperature range 1.8 to 16 K). For **1-Dy**: Turquoise to orange squares represent data extracted from ac magnetic susceptibility measurements, and green squares represent data extracted from dc relaxation experiments (1.8 to 2.5 K). For **3-Dy**: Red to orange circles represent data extracted from ac magnetic susceptibility measurements (process 2), and blue circles represent data extracted from dc relaxation experiments (temperature range 1.8 to 9 K). For **1-Dy**, the black line describes a fit to a Raman and an Orbach relaxation process. For **3-Dy**, the black line represents a fit to a QTM, a Raman, and an Orbach relaxation mechanism.

The experimentally determined *U*_eff_ for **1-Dy** is smaller than the value of *U*_eff_ = 87.8(3) cm^–1^ obtained for [(Cp*_2_Dy)_2_(μ-bpym^•^)]­(BPh_4_), indicating that the transfer integral *t* in the HM (eq 3) is reduced when the “1,1′-biphenyl” is switched to a “[4a,8a]-naphthalene” binding pocket.

For **3-Dy**, considering the generalized Debye model alone was insufficient to yield a satisfactory fit of the χ_M_″ peaks (Figures 7 and S22). Hence, a double generalized Debye model was employed to describe the ac data of **3-Dy**, affording two distinct Orbach relaxation pathways with *U*_eff(1)_ of 137(4) cm^–1^ and *U*_eff(2)_ of 143(3) cm^–1^ (Figure S42). The simultaneous occurrence of two Orbach relaxation processes has been observed for a few other radical-bridged Ln SMMs such as [(Cp*_2_Dy)_2_(μ-5,5′-(OEt)_2_bpym^•^)]­(BPh_4_) or [K­(crypt-222)]­[(Cp^Me4H^_2_Tb)_2_(μ-N_2_^•^)].^25,26^

While the origin of the multiple Orbach relaxation pathways remains unexplored, we hypothesize that the presence of two crystallographically distinct Dy^III^ centers could be at play for **3-Dy**. Notably, both relaxation barriers, *U*_eff_, determined for **3-Dy** are a record for dinuclear radical-bridged SMMs bearing an organic radical. The values for *U*_eff_ exceed considerably the current highest for an organic radical-bridged dinuclear system of 93 cm^–1^ for [(Cp*_2_Dy)_2_(μ-5,5′-F_2_bpym^•^)]­(BPh_4_), and even the highest *U*_eff_ value of 108.1(2) cm^–1^ for a N_2_^3–•^ radical-bridged dysprosium complex [K­(crypt-222)]­[(Cp^Me4H^_2_Dy)_2_(μ-N_2_^•^)].^25,26^

For **3-Dy**, dc relaxation experiments were conducted between 1.8 and 9 K. The resulting magnetization decay curves were fit to a stretched exponential function, eq 1 (Figures S28–S36). The relaxation times derived from both ac magnetic data (for process 2) and dc relaxation data gave a curved Arrhenius plot innate to temperature-independent τ below 7 K, which for successful modeling required the inclusion of Raman and QTM processes in addition to the Orbach relaxation mechanism (Figures 8 and S43). The obtained spin-reversal barrier *U*_eff_ = 143(2) cm^–1^ and attempt time τ_0_ = 1.8(3) × 10^–9^ s are effectively invariant to the values obtained from fitting of the ac magnetic susceptibility data alone. All fit results are summarized in Table S8. An inspection of the relaxation times for **1-Dy** and **3-Dy** reveals that at 1.8 K the τ value is larger for **1-Dy**, but with increasing temperatures, it is quickly surpassed by **3-Dy**.

Strikingly, the *U*_eff_ value of **3-Dy** is approximately five times larger than that of **1-Dy**, primarily induced through oxidation state change of the organic radical bridge—an unparalleled circumstance for radical-bridged SMMs. It is also remarkable in the context of select theories which identified magnetic coupling strength as the main parameter to impact the barrier height in radical-bridged systems.^16,24^ However, in our case a weaker coupling together with a topologically similar ligand field results in much slower magnetic relaxation for **3-Dy**.

For completeness, the dynamic magnetic properties of **2-Dy** were canvassed (Figures S20 and S21). The presence of two highly anisotropic Dy^III^ ions, albeit uncoupled owing to the diamagnetic flv^2–^ bridge, may engender slow magnetic relaxation, attributable to the single-ion effect. Indeed, under an ac magnetic field of 3 Oe and between frequencies of 0.1 and 1000 Hz, slow magnetic relaxation was observed where the maximum of the single χ_M_″ peak moved over the entire probed temperature range from 4.0 to 23.0 K, suggestive of a thermally activated relaxation mechanism. The extracted relaxation times were satisfactorily fit to a single Raman process, yielding a *C* of 1.08(6) × 10^–3^ s^–1^K^–*n*^ and *n* of 4.87(2) (Figure S41). Considering additionally an Orbach process did not afford a satisfactory fit. The faster magnetic relaxation in **2-Dy**, relative to **1-Dy** and **3-Dy**, arises from uncoupled magnetic moments and thus, renders the first coordination sphere of each Dy ion more relevant. However, the local environment for each Dy in **2-Dy** is not axial, ultimately causing a faster magnetic relaxation (Figure S44).

### Magnetic Hysteresis

To explore the utility of a single-molecule magnet, variable-field magnetization data were collected on polycrystalline samples of **1-Dy**, **2-Dy**, and **3-Dy** between ±7 T using an average sweep rate of 0.01 T/s (Figures 9, 10, S45–S50).

**Figure 9:**
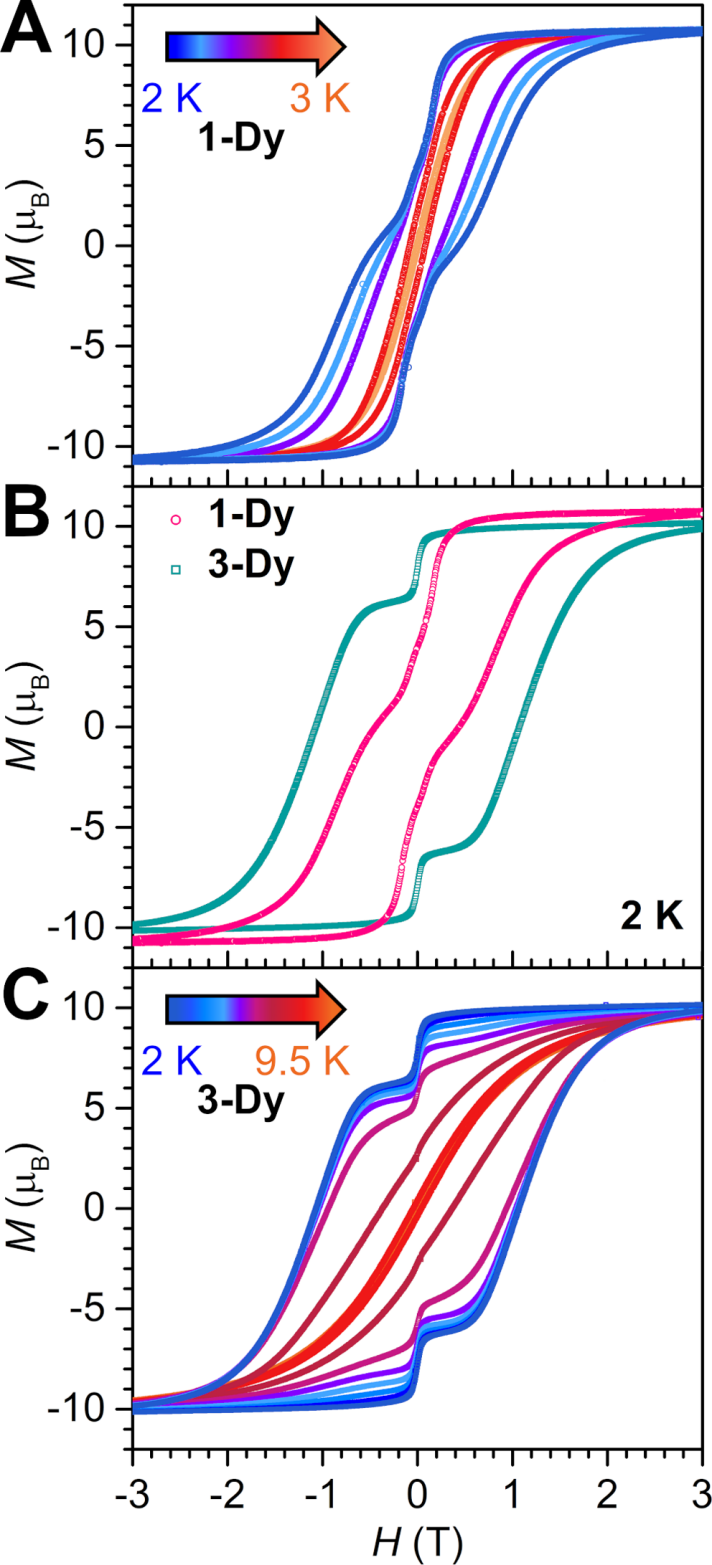
Plot of magnetization (*M*) vs dc magnetic field (*H*) at an average sweep rate of 0.01 T/s for **1-Dy** from 2 to 3 K (**A**), **1-Dy** and **3-Dy** at 2 K (**B**), **3-Dy** from 2 to 9.5 K (**C**).

**Figure 10:**
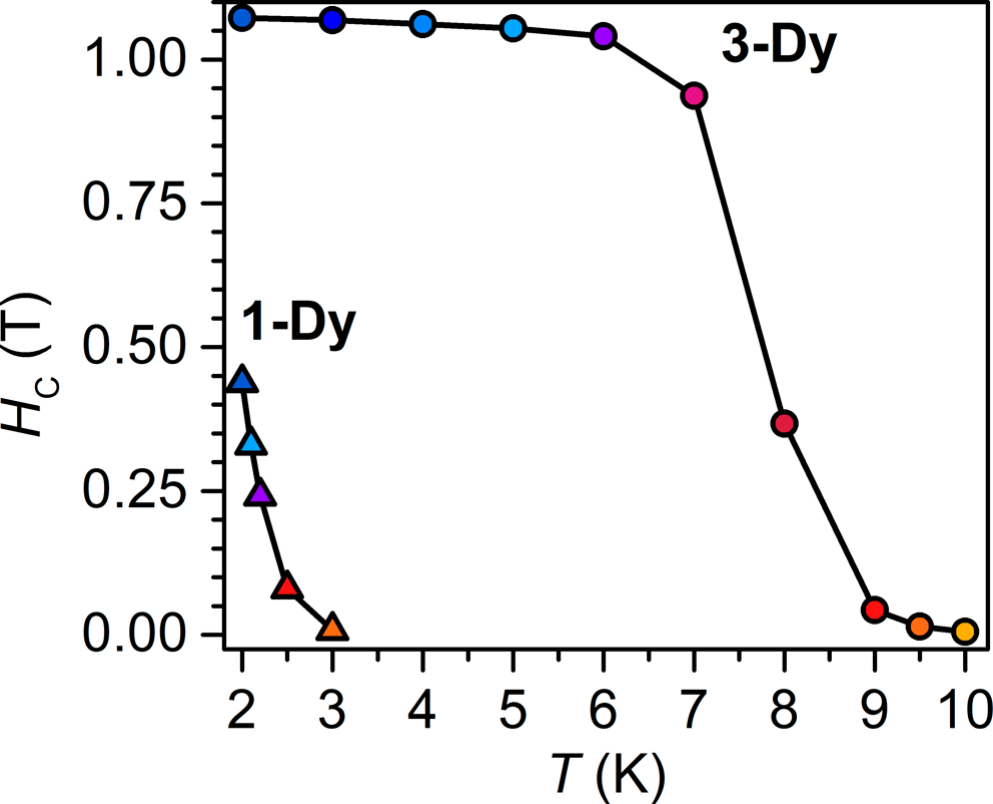
Plot of coercive field vs temperature for **1-Dy** (triangles) and **3-Dy** (circles), obtained from variable-field magnetization (*M*) data collected at a sweep rate of 0.01 T/s. Solid lines are guides for the eye.

As supported by the results of the field- and zero-field-cooled magnetic susceptibility measurements and dc relaxation experiments, **1-Dy** exhibits an open magnetic hysteresis loop at 2.0 K, with a coercive field, *H*_C_, of 0.439 T. With increasing temperatures, the coercive field decreases, until the hysteresis eventually closes at 3.0 K (Figures 9 and S45). *H*_C_ steadily declines over the probed temperature range, in accordance with the presence of a thermally activated relaxation process. The hysteresis loops adopt a smooth shape with a subtle step at 0 T field, indicative of effective suppression of ground state or resonant QTM initiated by strong magnetic coupling of the flv^1–•^ and Dy^III^ magnetic moments.

The *H*_C_ of 0.439 T for **1-Dy** ranges among the largest recorded for radical-bridged dinuclear SMMs and approaches that of the tetrazine radical-bridged didysprosium complex [(Cp*_2_Dy)_2_(μ-dmtz^•^)­(THF)_2_]­(BPh_4_)·THF (dmtz = 3,6- dimethoxy-1,2,4,5- tetrazine) with *H*_C_ = 0.50 T (1.8 K),^36^ and the bipyrimidine radical-bridged didysprosium complex [(Cp*_2_Dy)_2_(μ-5,5′-F_2_bpym^•^)]­(BPh_4_) (bpym = 2,2′-bipyrimidine) with *H*_C_ = 0.58 T (2 K),^26^ respectively.

The variable-field magnetization data collected for **3-Dy** (Figures 9, S47–S49) exhibit wide open hysteresis loops, with a coercive field of 1.072 T at 2 K. The coercive field, *H*_C_, remains essentially unchanged up to 6 K (*H*_C_ = 1.040 T) and diminishes by ∼65% to 0.367 Oe at 8 K (Figures 9 and 10). The loops remain open until 9.5 K (*H*_C_ = 142 Oe at 9.5 K), where this temperature represents a record high for organic radical-bridged dinuclear Dy^III^ SMMs. At 10 K, the hysteresis loop is effectively closed as the coercive field is smaller than the sweep rate (Figures S48 and S49). The maximum *H*_C_ found for **3-Dy** of 1.072 T constitutes the second highest *H*_C_ value reported for any organic radical-bridged didysprosium complex and even exceeds that of *H*_C_ = 1 T at 5.5 K for [K­(crypt-222)]­[(Cp^Me4H^_2_Dy)_2_(μ-N_2_^•^)] bearing the inorganic dinitrogen radical N_2_^3–•^.^25,90^ Hence, the magnetic hysteresis of **3-Dy** highlights that precise orbital engineering is equally important as maximizing magnetic exchange coupling for realizing magnetic memory effects in multinuclear SMMs.

The dinuclear SMM [K­(crypt-222)]­[(Cp*_2_Dy)_2_(μ-Bbim^•^)] containing a bisbenzimidazole radical anion bridge (Bbim^3–•^), constitutes a structural isomer of flv^3–•^, rendering a comparison to **3-Dy** suitable.^30^ Specifically, the impact of the distinct binding pockets onto magnetic hysteresis behavior are compared in a set of bimetallic complexes bearing the same charge, but a slightly different local coordination geometry.

For the Bbim^3–•^-bridged complex, the *H*_C_ of 0.526 T at 2 K is approximately halved in value compared to **3-Dy**, and the hysteresis loops are open below 5.5 K, which temperature is lower than 9.5 K observed for **3-Dy**. Hence, speaking in terms of the Hubbard Model, the overlap integral *t* was improved by the mere variation of the central C–C bond of the bridging ligand. Likewise, *U* was augmented as the *E*_1/2_ of the Bbim complex is considerably less negative with −1.02(8) V in comparison to the −1.898(4) V determined for **3-Dy** and the −2.5 V for the Dy^III^/Dy^II^ couple, respectively.^42,43^

Furthermore, variable-field magnetization measurements were also carried out for **2-Dy**, Figure S46. **2-Dy** exhibits slightly open magnetic hysteresis loops up to 4 K which are vastly different in appearance relative to **1-Dy** and **3-Dy**. This is best reflected in the substantially diminished opening of the loops with a maximum coercive field of 682 Oe at 2.0 K in **2-Dy**. Rapid quantum tunnelling is operative in **2-Dy** on the timescale of the magnetic hysteresis measurements, which gives rise to pronounced steps in the hysteresis loops close to 0 Oe fields. The origin of fast QTM in **2-Dy** is attributed to the presence of two noninteracting Dy^III^ ions. The magnetic hysteresis comparison of **1-Dy**, **2-Dy**, and **3-Dy** demonstrates powerfully the impact of implementing a radical to promote strong magnetic exchange coupling between two lanthanide ions, leading to a more effective suppression of undesirable quantum tunneling of the magnetization and, as a result, to a genuine magnetic memory effect (Figure S50).

Field-dependent magnetization measurements (*M* vs *H*) were performed on **1-Ln**, **2-Ln**, and **3-Ln** between 0 and 7 T from 2 to 10 K (Figures S51–S56). The *M* vs *H* curves for **1-Dy** and **3-Dy** are S-shaped in the isothermal lines at 2 K, which corroborates with the observed magnetic blocking. For **1-Dy**, this S-shape transitions into a steadily increasing curve at 4 K, while the S-shape of the curve remains up to 8 K for **3-Dy**, alluding to the much higher magnetic anisotropy present in **3-Dy** relative to **1-Dy** (Figures S52 and S56). By contrast, the reduced magnetization curves of the Gd complexes **1-Gd** and **3-Gd** are superimposable at all temperatures, as expected for the approximately isotropic Gd^III^ ions (Figures S51 and S55).

Although **3-Dy** exhibits considerably slower magnetic relaxation relative to **1-Dy**, the hysteresis loops of **3-Dy** feature pronounced steps at 0 Oe, indicative of the presence of ground state QTM. This is not *a priori* intuitive, given higher *H*_C_ and hysteresis temperatures suggest a more efficient suppression of competing relaxation pathways. However, this behavior is also reflected in the Arrhenius plot, where τ remains unchanged until ∼6 K. Hence, the origin of the massively enhanced magnetic hysteresis of **3-Dy** in terms of *H*_C_ and hysteresis temperature is hypothesized to relate to spin-phonon coupling, meaning the energy overlap between magnetic states and phonons (or molecular vibrations). In exchange-coupled systems, the relaxation barrier is often defined by the first excited exchange-coupled state, typically equaling the experimentally determined value for *U*_eff_. In theory, the separation is directly related to the exchange coupling strength *J* - for Dy^III^ by 15 |*J*|.^26^ This prediction is true as long as the single-ion anisotropy of the Dy^III^ ions is high, in which case the low-lying exchange-coupled states arise from the single-ion *m*_*J*_ = ^15^/_2_ states alone. Admixture of higher-lying *m*_*J*_ states will lower the energy of the excited exchange states.^26^

For **1-Dy**, *U*_eff_ is with 28.36 cm^–1^ relatively small, and molecule-inherent low-energy vibrations such as Cp* rotations are accessible and could potentially match the energy of the magnetic states, providing pathways for demagnetization. By contrast, for **3-Dy**, *U*_eff_ is with 143(2) cm^–1^ approximately five times higher than for **1-Dy**, and therefore the first excited state is considerably elevated, inherently reducing the number of low energy vibrations that overlap with the energy of the exchange-coupled excited state. Unfortunately, the energy scales discussed here are far too low to be experimentally accessible via most conventional IR spectrometers (the minimum accessible frequency is 600 cm^–1^ in our case). Instead, we evaluate in the following the low-energy vibrational spectra via computational density functional theory (DFT) methods to identify suitable vibrations that match the *U*_eff_ values of **1-Dy** and **3-Dy**.

Considering the applied energy shift, the performed frequency calculations for all three Gd complexes are in good agreement with the experimental spectra, which are nearly superimposable with the experimental data for the Dy complexes, allowing a comparison of the calculated vibrational modes with the Dy compounds (Figures S4–S6). For **1-Gd**, one vibrational mode is found at 31.7 cm^–1^ with two rocking motions of the Cp*_2_ framework, and one torsional oscillation motion involving the peripheral phenyl rings of the flv^1–•^ that results in an out-of-plane displacement of the metal centers relative to the bridging ligand. The excellent overlap between these vibrations, and the presence of multiple potential vibrations, emphasizes the amplified magnetic relaxation in **1-Dy**. For **3-Gd**, one vibrational mode is found in excellent agreement with **3-Dy** at 143.3 cm^–1^, which exhibits an umbrella mode motion of the Cp*_2_ frameworks resulting in the metal centers being pushed out-of-plane with respect to flv. The slight discrepancies in the calculated vibrations and the experimentally determined *U*_eff_ values are ascribed to the difference in atomic coordinates in the calculation due to starting with optimized geometries.

These findings show that vibronic coupling is indeed detrimental to the magnetic relaxation in both Dy complexes, and the increased charge of the bridging ligand in **3-Dy** shifts the magnetic levels and low-energy vibrational modes out of resonances below 100 cm^–1^, enabling magnetic hysteresis up to almost 10 K.

To deepen our understanding of the differing mechanisms involved in the magnetism of **1-Dy** and **3-Dy**, the hysteresis loops were further analyzed. A magnetic hysteresis loop can be modeled following a summation of arctan functions and a constant offset to account for baseline shifts, eq 6.^91^ The number of arctan functions employed will vary depending on the number of different demagnetization processes exhibited in the hysteresis loop. Here *M*, *M*_Si_, *H*, and *H*_Ci_ correspond to magnetization, saturation magnetization of the *i* ^th^ process, magnetic field, and the coercive field for the *i* ^th^ process, respectively.

6 $$M\left(H\right)=\frac{2}{\pi }\sum_{i=1}^{4}M_{\mathrm{Si}}\mathrm{arc}\tan\left(a_{i}\left(H-H_{\mathrm{Ci}}\right)\right)+\mathrm{baseline}$$

The derivative of the hysteresis data emphasizes the inflection points on the loop corresponding to different demagnetization processes. Thus, the magnetic hysteresis loops obtained from 2 to 3 K for **1-Dy**, and from 2 to 9.5 K for **3-Dy**, were fit employing arctan functions, followed by calculating the first derivatives of the fits of the backward sweep data (Figures S57, S58, and S59). The first derivative of an arctan function follows a Lorentzian distribution and is analyzed with a Cauchy probability distribution function (CPDF), eq 7.^92^

7 $$\frac{dM}{dH}=2M_{\mathrm{Si}}\cdot \frac{1}{\pi \gamma_{i}{(1+\left(\frac{H-H_{\mathrm{Ci}}}{\gamma_{i}}\right)}^{2}}$$

The γ represents the half-width at half-maximum of a given peak and *M*_Si_ corresponds to the peak amplitude of the peak centered at *H*_Ci_. Thus, this model enables locating the field positions and the broadness for each demagnetization process observed in the hysteresis loop. Furthermore, the peak amplitudes allow a quantification of percent contribution from each process. This method has been successfully implemented in the study of hysteresis loops of Er^III^-based SMMs.^93^

The CPDF analyses of the hysteresis loops of **1-Dy** and **3-Dy** gave four and three distinct demagnetization processes for each compound, respectively (Tables S9 and S10). Specifically, data from the reverse sweeps (from 7 T to −7 T) of the hysteresis loops were used for this analysis. For **1-Dy**, the four processes are centered around P1 = 0.16 T, P2 = −0.07 T, P3 = −0.50 T, and P4 = −0.97 T at 2 K. From these, P1 and P2 exhibit narrow peaks while P3 and P4 are broader, Figure 11. Considering the percent contributions for demagnetization, P4 is prevalent with 52.4%, while P1, P2, and P3 display 24.8, 14.0, and 8.8% contributions, respectively. Processes P2, P3 and P4 exhibit an upward trend with rising temperature, while P1 remains largely uniform. The field positions of P1 and P2 are the same until 2.5 K, where the hysteresis loops start becoming ill-defined and narrower. The magnitude of the field positions of P3 and P4 gradually decline from 2 to 3 K, showcasing that these demagnetization processes are affected by temperature changes.

**Figure 11:**
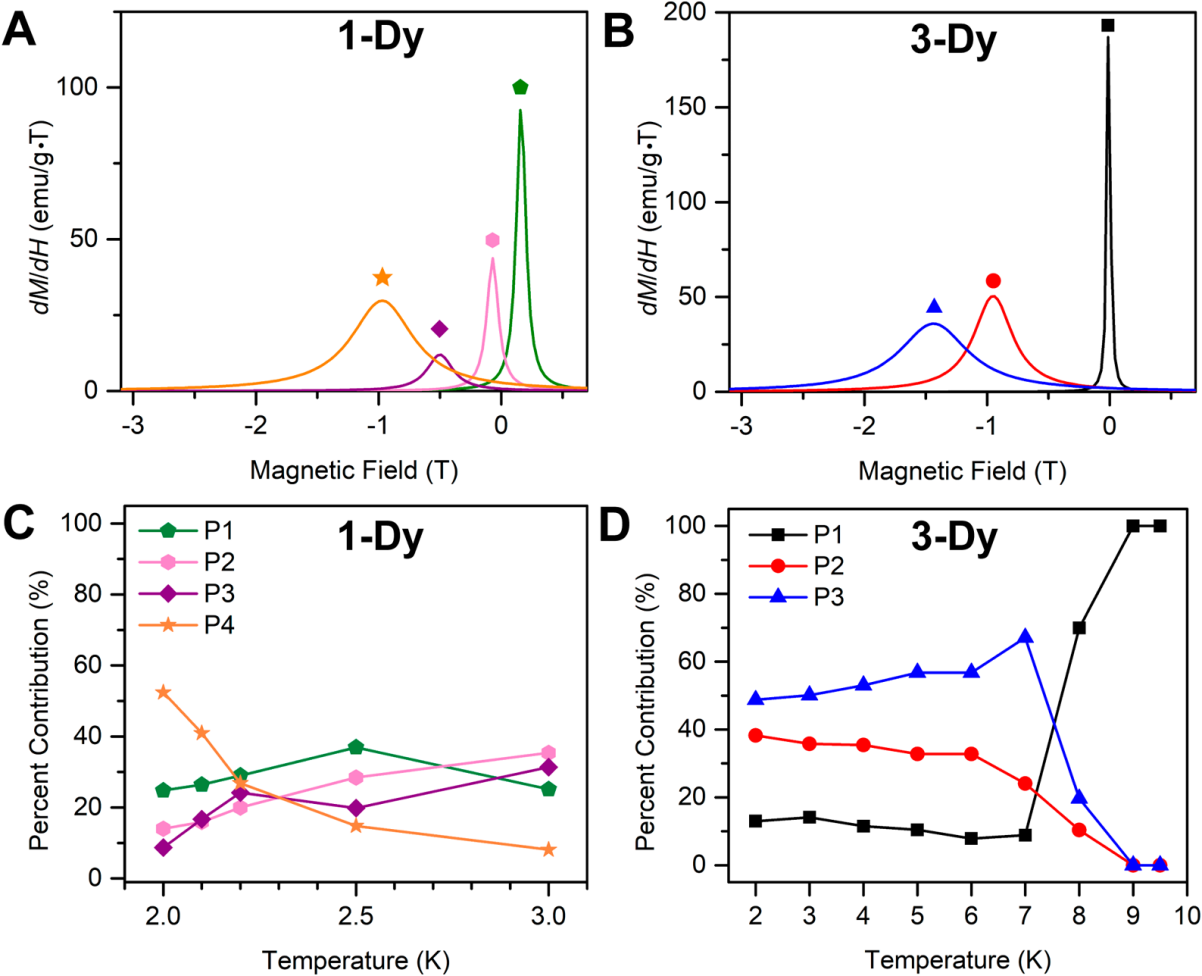
Cauchy probability distribution function analysis performed on the hysteresis loops of (A) **1-Dy** and (B) **3-Dy**. Peaks marked with a green pentagon, pink hexagon, purple diamond, and orange star represent P1, P2, P3, and P4 demagnetization events for **1-Dy**. Peaks marked with a black square, red circle, and a blue triangle correspond to P1, P2, and P3 demagnetization processes observed for **3-Dy**. Plots (C) and (D) showcase the variation of percent contributions of each demagnetization process with rising temperature.

The CPDF analyses of the hysteresis loops of **3-Dy** yielded three distinct demagnetization processes. At 2 K, the field positions for the three processes are P1 = −0.01 T, P2 = −0.95 T, and P3 = −1.44 T with percent contributions of 13.0, 38.3, and 48.7%, respectively. With rising temperature, the P1% contribution changes with no clear temperature dependence, whereas P2 and P3 exhibit a downward trend in both the percent contribution and the absolute value of the magnetic field corresponding to the peak positions. Above 8 K, as the hysteresis loops get much narrower, the P2 and P3 processes are undetectable on the first derivative, leading to 100% contribution from P1. The peak around 0 T in **3-Dy** (P1) is attributed to a QTM relaxation owing to the very narrow nature of the peak, and the temperature invariance of its percent contribution.

By contrast, the two peaks closer to 0 T for **1-Dy** cannot be unambiguously ascribed to the QTM relaxation mechanism, as they are broader and exhibit temperature dependence. The P4 of **1-Dy** and the P2 of **3-Dy** are of interest due to these processes emerging around the same field position (−0.97 T and −0.95 T) and both exhibiting a temperature dependence, where the percent contribution is decreased with rising temperature. In fact, these demagnetization processes are tentatively assigned to stem from Orbach relaxation mechanisms owing to an inversely proportional relationship with temperature. In sum, the CPDF analysis uncovers that the various demagnetization events respond differently as a function of temperature, where some events may originate from multiple competing relaxation mechanisms.

### Broken Symmetry DFT Calculations

Broken symmetry DFT calculations were carried out on unoptimized crystal coordinates of all three flv-bridged Gd complexes, **1-Gd**, **2-Gd**, and **3-Gd**, excluding counterions. For **1-Gd** and **3-Gd**, the spin delocalized on the flv ligand was flipped with respect to the Gd centers by employing the spinflip function of ORCA, whereas for **2-Gd**, the spin on one of the Gd centers was flipped in relation to the other. The computations were conducted using seven different functionals to determine the functional that would best align with the experimentally derived magnetic exchange coupling between the metal center and the radical in the systems (Tables S14–S16). The exchange coupling constant (*J*_calc_) was determined through the *J*_calc_ = – (*E*_HS_ – *E*_BS_)/(⟨*S* ^2^⟩_HS_ – ⟨*S* ^2^⟩_BS_) formalism. Here, *E*_HS_ and *E*_BS_ are the energies of the high spin and the broken symmetry states, respectively, and ⟨*S* ^2^⟩_HS_ and ⟨*S* ^2^⟩_BS_ represent the spin expectation values of the high spin and broken symmetry states.^94^ Based on the predicted *J*_calc_ values from all functionals, the TPSS0 functional produced parameters that are in excellent agreement with the experimentally derived values. The experimental and calculated *J* values and EPR parameters, respectively, are listed in Table 2. The trend for the magnitude of the exchange coupling constant (*J*_Gd–Rad_) derived from the experiment is also accurately reflected in the broken-symmetry calculations, where **1-Gd** shows a *J*_calc_ that is approximately twice as large as that for **3-Gd**.

**Table 2:** Calculated and Experimental Values for Exchange Coupling Constant (*J*), *g*-Value, Zero-Field Splitting Parameters (*D* and *E*/*D*)

compound	*J*_calc_ (cm^–1^)	*J*_exp_ (cm^–1^)	*g*_calc_	*S*_exp_	*g*_exp_	*D*_exp_ ^a^ (cm^–1^)	*E*/*D*_exp_ ^a^
**1-Gd**	–14.01	–10.5(2)	2.004	^13^/_2_	1.990	–0.0444	0.079
**2-Gd**	–0.02	–0.005(2)	-	^7^/_2_	1.982	–0.0517	0.055
**3-Gd**	–8.82	–5.3(1)	2.005	^13^/_2_	1.987	–0.0370	0.117

a Values obtained from simulation of high-field EPR spectra. The line width peak-to-peak of 30 mT was used for all simulations (Voigt line shape as convolution of 50% Gaussian and 50% Lorentzian contribution).

The DFT predicted *J*_calc_ values are slightly overestimated compared to the experimental values. Figure 12 depicts the spin density distribution generated from the broken-symmetry calculations. The spin density distributions of **1-Gd** and **3-Gd** are spread across the flv ligand and the two metal centers. By contrast, the spin density distribution is primarily localized on the metal centers in **2-Gd**. This interpretation reflects the essentially uncoupled Gd ions in **2-Gd** due to the presence of a diamagnetic bridge, whereas the radical bridge in each **1-Gd** and **3-Gd** engenders strong magnetic exchange coupling.

**Figure 12:**
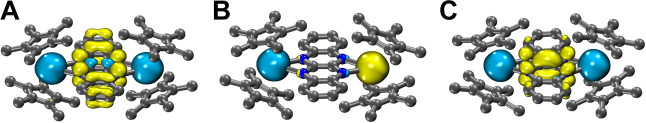
Spin density distributions of broken-symmetry states of (A) **1-Gd**, (B) **2-Gd**, and (C) **3-Gd**. Yellow and light blue surfaces indicate different phases of spin density. Orange, blue, and gray spheres represent Gd, N, and C atoms, respectively. Gd atoms are fully covered by spin density surface and thus, appear invisible. All surfaces were generated at isovalue of 0.003.

Since the Gd centers mainly interact with the N atoms of the flv radicals, the magnitudes of spin density generated for those atoms were investigated via broken-symmetry calculations. The obtained values imply a higher average spin density on the N atoms of **1-Gd** relative to that of **3-Gd**. This trend is also reflected in the Mulliken spin populations averaged for N atoms in the two molecules (–0.188 for **1-Gd** and –0.136 for **3-Gd**, Table S17). The computational results arrive at the conclusion of the N atoms in flv^3–•^ displaying a lower spin density compared to flv^1–•^ which also coincides well with the hyperfine coupling constants experimentally derived for the yttrium congeners.^10^ Such spin density values for the N atoms may be correlated to the *J* values determined for the complexes, where a higher spin density is expected to engender stronger magnetic interaction with the metal centers. In substituted bpym radical-bridged Gd complexes, higher spin densities on the nitrogen atoms bound to the metal centers were shown to augment the magnitude of *J*.^26^

Furthermore, the *J*_Gd–Rad_ values attained for the Gd complexes can be employed to estimate the *J*_Dy–Rad_ value for the isostructural Dy complexes.^35^ Here, taking into account the ^5^/_2_ spin of the Dy^III^ ions, the corresponding *J*(Gd) is multiplied by 1.4,^35^ which results in *J*(Dy) values of –14.6 cm^–1^ and −7.4 cm^–1^ for **1-Dy** and **3-Dy**, respectively. These values provide adequate estimates. However, they are approximations as only the spin operator of the lanthanide ions was considered. A comprehensive picture of the exchange coupling in this type of radical-bridged Dy complexes is very intricate and requires taking into account anisotropy, single ion effects, state mixing and other factors, which altogether is very expensive. As such, the calculated estimates provide a cost-effective assessment of the coupling strength in strongly coupled Dy SMMs. We also would like to note that this type of assessment of *J* values has been successfully demonstrated for Dy complexes that contain either Dy–radical or Dy–Dy interactions.^35,95^

### High-Field Electron Paramagnetic Resonance (HF-EPR)

The Gd^III^ ion possesses a 4f^7^ electronic configuration with total spin *S* = ^7^/_2_ and orbital angular momentum *L* = 0. Because of this, there are no first-order orbital contributions to its magnetic properties. Therefore, Gd^III^ compounds typically display weak zero-field splitting (ZFS) interactions, making them easy targets for EPR investigations.

Figure 13 displays temperature-dependent powder EPR data at both low- and high-fields/frequencies for **3-Gd**, which gave by far the highest quality spectra; see Figures S61 and S62 for the corresponding data recorded for **1-Gd** and **2-Gd**. The first thing to note from the lowest temperature 52 GHz data in Figure 13 is that there are six evenly spaced resonances on the low-field side of the strong central feature (corresponding to the *m*_*S*_ = –^1^/_2_ to ^1^/_2_ transition). In the case of an eas*y*-axis system (*D* < 0, *vide infra*) with total spin, *S*, one expects 2*S* resonances for each component of the spectrum, i.e., 7 parallel (*B*||*z*) resonances for Gd^III^, with three either side of the central line. Focusing on the low-field side, which is not contaminated by perpendicular spectral components at the lowest temperatures, the observation of six resonances (black dots) rules out the possibility that the spectra can be due to uncoupled Gd^III^ ions. In fact, the six resonances suggest a total spin ground state of *S* = ^13^/_2_, i.e., 13 total parallel resonances, with six either side of the central resonance. As such, the 52 GHz spectra for **3-Gd** provide spectroscopic proof that its ground state is coupled, corresponding to two Gd^III^ ions coupled antiferromagnetically to a radical, i.e., *S*_Tot_ = ^7^/_2_ – ^1^/_2_ + ^7^/_2_ = ^13^/_2_.

**Figure 13:**
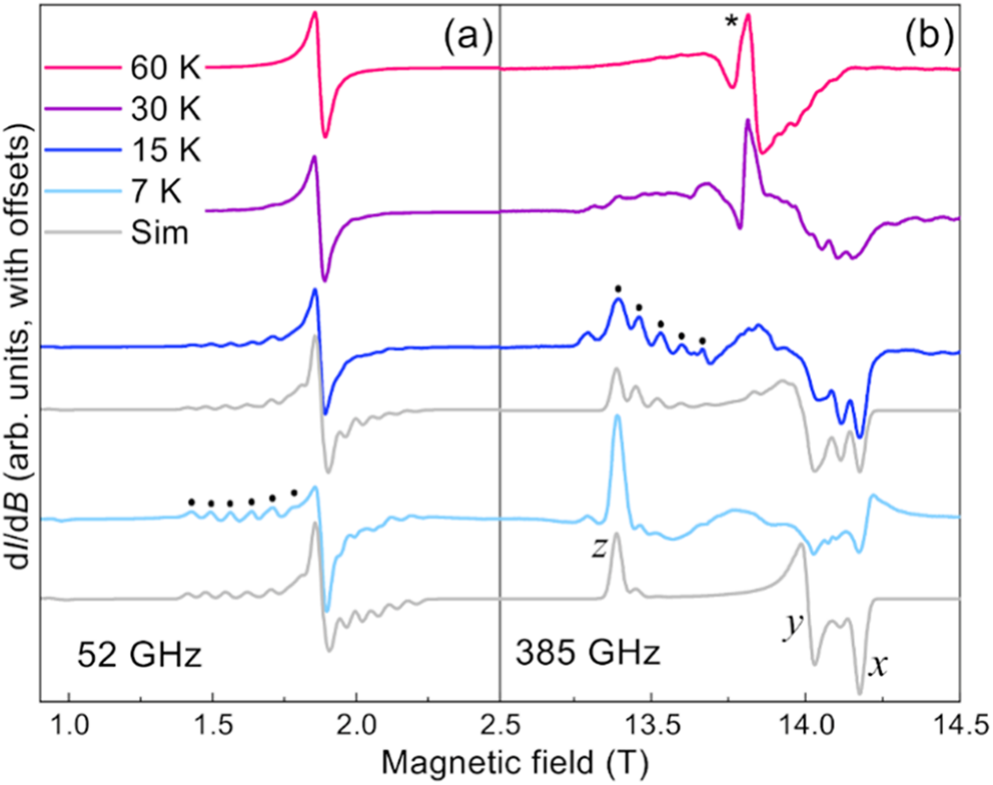
Temperature-dependent powder EPR spectra collected for **3-Gd** at frequencies of 52 GHz (a) and 385 GHz (b); the spectra are recorded in derivative mode, d*I*/d*B*, where *I* is the microwave intensity transmitted through the sample and *B* is the local magnetic field; see legend for corresponding temperatures. Simulations (“Sim” – gray lines) according to eq 8 are shown below the corresponding experimental spectra for the two lowest temperatures at both frequencies. These simulations were generated using a single set of spin Hamiltonian parameters given in Table S18. The black dots mark parallel (***B*** ||*z*) resonances on the low-field side of the *m*_*S*_ = –^1^/_2_ to ^1^/_2_ transition (seen as the strong central feature at 52 GHz but not seen at 385 GHz due to depopulation of the *m*_*S*_ = –^1^/_2_ level). One can infer a coupled effective spin *S* = ^13^/_2_ ground state based on the number of parallel resonances on the low-field side of the central transition, as discussed in the main text. The asterisk denotes a broad radical signal at high temperatures, suggesting decoupling of the spins above 15 K.

The reason for the strong central resonance at 52 GHz is because the *m*_*S*_ = –^1^/_2_ to ^1^/_2_ transition has the strongest matrix element, minimal broadening, and it is thermally populated at relatively low temperatures due to the weak Zeeman splitting at *B* < 2 T. At 385 GHz, the spectra have a very different appearance.

At the lowest temperature (7 K), only the lowest spin sublevel (*m*_*S*_ = –^7^/_2_) is significantly populated. Hence, one only observes three strong resonance features corresponding to the *z* and *x*, *y* components of the spectrum (with much weaker features due to minor populations in excited *m*_*S*_ levels). Simulation of these features allows us to constrain the parameters in the following effective spin Hamiltonian, eq 8,

$${Ĥ}^{\mathrm{eff}}=\mu_{B}gB\cdot Ŝ+D{Ŝ}_{z}^{2}+E\left({Ŝ}_{x}^{2}-{Ŝ}_{y}^{2}\right)+B_{4}^{0}{Ô}_{4}^{0}$$ 8

where the first term represents the Zeeman interaction assuming an isotropic *g*-factor; ***B*** is the applied field vector, ***Ŝ*** the total spin operator (with components *Ŝ*_*i*_, *i* = *x*,*y*,*z*), and μ_B_ the Bohr magneton. The remaining terms characterize the ZFS: *D* (*E*) parametrizes the axial (rhombic) interaction, while the last term represents a small fourth-order axial contribution, expressed in terms of the extended Stevens operator *Ô*_4_^0^ with corresponding coefficient *B*_4_^0^.^56^

At 385 GHz and the lowest temperature (Figure 13), the relative spacing between the *x* and *y* resonances constrains *E*, while the separation between the *z* resonance and the midpoint between *x* and *y* constrains *D*. The *g*-factor then centers the spectrum so that the three resonances occur at the correct magnetic fields. In order to then capture the relative spacings of the six resonances seen in the 52 GHz spectra, we found it necessary to include a small fourth-order axial interaction.^96^ As can be seen, simulations of the lowest two temperature spectra at both frequencies are quite satisfactory with respect to the resonance positions, with the exception of a small peak at fields just below the strongest (ground state) *z*-component resonance. The source of this peak is not clear but could be due to a minority species with slightly larger *D*-value. Discrepancies in the intensities may be due to partial alignment of crystallites in the powder,^97^ which favors the parallel (***B*** ||*z*) components, thus accounting for their stronger intensity. The optimal effective spin Hamiltonian parameters for **3-Gd** are *S*_Tot_ = ^13^/_2_, *g* = 1.987, *D* = −0.0370 cm^–1^, *E*/*D* = 0.117, and *B*_4_^0^ = 1.67 × 10^–6^ cm^–1^.

As can be seen at 385 GHz, a new central resonance appears (marked by asterisk in Figure 13) for **3-Gd** at the two highest temperatures. This feature cannot be reproduced with the effective *S* = ^13^/_2_ Hamiltonian of eq 8, suggesting that the Gd^III^ ions begin to uncouple from the radical. We believe that this is due to exchange averaging at elevated temperatures so that the radical EPR signal appears at its uncoupled position close to *g* = 2.^98^ Because the radical is immersed in a paramagnetic Gd^III^ background, this resonance lacks hyperfine structure and has a relatively broad line width (for a radical). This observation suggests that the system becomes uncoupled between 15 and 30 K, consistent with the exchange coupling parameter determined from the χ_M_ *T* data, i.e., |*E* ^exch^| ≈ 2*J*_exp_ *S*_Gd_ *S*_rad_ = 18.5 cm^–1^ (≅ 27 K), where *S*_Gd_ = ^7^/_2_ and *S*_rad_ = ^1^/_2_, respectively, denote the spin values of the Gd^III^ ions and the radical.

Similar analyses for **1-Gd** and **2-Gd** are presented in the Supporting Information (Figures S61 and S62, respectively), and the obtained effective spin Hamiltonian parameters are given in Tables 2 and S18; **1-Gd** can again be modeled as a coupled effective *S*_Tot_ = ^7^/_2_ – ^1^/_2_ + ^7^/_2_ = ^13^/_2_ spin, while **2-Gd** is modeled as uncoupled *S* = ^7^/_2_ Gd^III^ spins due to the absence of a radical on the organic bridge. The uncoupled radical signal only appears at the highest temperature in the case of **1-Gd**, suggesting that the system becomes uncoupled at a higher temperature in comparison to **3-Gd**, i.e., between 30 and 60 K. This is consistent with the factor of 2 increase in *J*_exp_ for **1-Gd** relative to **3-Gd**, giving |*E* ^exch^ | ≈ 37 cm^–1^ (≅ 54 K).

## Conclusions

The synthesis of a series of fluoflavine (flv) bridged dilanthanide complexes, featuring the bridging flv ligand in three different oxidation states 1–, 2– and 3–, is presented. Specifically, two series of fluoflavine radical-bridged complexes were isolated: (a) the cationic complexes [(Cp*_2_Ln)_2_(μ-flv^•^)]­[Al­(OC­{CF_3_}_3_)_4_] (**1-Ln**, where Ln = Gd, Dy) containing a flv^1–•^ radical bridge were synthesized from chemical oxidation of the neutral compounds [(Cp*_2_Ln)_2_(μ-flv)] (**2-Ln**) bearing a diamagnetic flv^2–^ bridging ligand, and (b) the anionic complexes [K­(crypt-222)]­[(Cp*_2_Ln)_2_(μ-flv^•^)] (**3-Ln**) comprising a flv^3–•^ radical were obtained from chemical reduction of **2-Ln**.

**1-Dy** and **3-Dy** are remarkable radical-bridged single-molecule magnets with hysteresis loops open below 3.0 K (**1-Dy**) and until 9.5 K (**3-Dy**), respectively. The temperature of 9.5 K sets a record of highest open hysteresis temperature for multinuclear SMMs containing an organic radical bridge. Notably, the energy barrier to spin-reversal is boosted 5-fold in **3-Dy** relative to **1-Dy** to a maximum *U*_eff_ of 143(2) cm^–1^, which constitutes a record for dinuclear SMMs composed of an organic radical bridge. Moreover, the determined magnetic exchange coupling strengths exhibit a striking trend, where *J*_Gd–Rad_ is −10.5(2) cm^–1^ for **1-Gd** and *J*_Gd–Rad_ is −5.3(1) cm^–1^ for **3-Gd** representing a halved value relative to **1-Gd**. This trend in coupling strength was confirmed via broken-symmetry density functional theory (BS-DFT) calculations.

The foregoing findings provide important insights for the future design of polynuclear lanthanide SMMs: (1) The magnetic coupling strength determined by fits to dc data of Gd complexes may not unveil SMM properties with hysteresis temperature and coercivity for the isostructural Dy congeners. (2) Higher charge on the radical bridging ligand results in a high-energy shift of the magnetically relevant vibrations, as was shown by the shift of the ground state to excited state vibration from **1-Dy** to **3-Dy**. (3) A low-lying first excited exchange state may readily match with low-energy vibrations of the peripheral ligand system such as Cp* rotations or wagging, and thereby enables easily accessible relaxation pathways. (4) Maximizing both the orbital overlap integral between 4f and ligand orbitals, *t*, by chemical design may enhance both *U*_eff_ and maximum hysteresis temperature, as was demonstrated by traversing from Bbim^3–•^ to the isomeric flv^3–•^ radical bridging ligand. (5) Minimizing the electronic repulsion, *U*, by matching a reduction potential of a complex with the Dy^III^/Dy^II^ redox couple, as realized by traversing from flv^1–•^ to flv^3–•^ amplifies magnetic hysteresis considerably.

In the future, we will tune both *t* and *U* further by exploring new radical bridges and overall complex architectures to amplify the magnetic blocking temperatures of multinuclear SMMs past the current limits.

## Supplementary Material



## Notes

Crystallographic refinement parameters, UV–vis-/NMR-/EPR- and IR spectra, cyclic voltammetry data, magnetic data, computational data, coordinates of optimized structures, and Python scripts for CPDF analysis (PDF)
