# Supplementary material for: Magnetic Blocking in Fluoflavine Radical-Bridged Dilanthanide Complexes
Source: J Am Chem Soc. 2025 Dec 10;147(51):47159–78. doi: 10.1021/jacs.5c14158 (PMC12752457; doi:10.1021/jacs.5c14158)
Supplement: Supplementary file 1 [file ja5c14158_si_001.pdf]

Supporting Information

for

Magnetic Blocking in Fluoflavine Radical-Bridged Dilanthanide Complexes

Florian Benner,[a] Saroshan Deshapriya,[a] Jakub Hrubý,[b] Stephen Hill,[b,c] and Selvan Demir*,[a]

[a]Department of Chemistry, Michigan State University, East Lansing, Michigan 48824, United States

[b]National High Magnetic Field Laboratory, Tallahassee, Florida 32310, United States

[c]Department of Physics and Department of Chemistry and Biochemistry, Florida State University, Tallahassee, Florida 32306, United States

*Email: sdemir@chemistry.msu.edu (S.D.)

Table of Contents

1 Single Crystal X-ray Diffraction S5

Table S1. Crystallographic data and structural refinement of $[(Cp^*{}_2Ln)_2(\mu\text{-flv}^\bullet)][Al(OC\{CF_3\}_3)_4]$ (**1-Ln**) and $[(Cp^*{}_2Ln)_2(\mu\text{-flv})]$ (**2-Ln**). S5
Table S2. Crystallographic data and structural refinement of $[K(crypt\text{-}222)][(Cp^*{}_2Ln)_2(\mu\text{-flv}^\bullet)]$ (**3-Ln**). S6
Table S3. Selected structural parameters of $[(Cp^*{}_2Ln)_2(\mu\text{-flv}^\bullet)][Al(OC\{CF_3\}_3)_4]$ (**1-Ln**) and $[(Cp^*{}_2Ln)_2(\mu\text{-flv})]$ (**2-Ln**). S7
Table S4. Selected structural parameters of $[K(crypt\text{-}222)][(Cp^*{}_2Gd)_2(\mu\text{-flv}^\bullet)]$ (**3-Gd**). S8
Table S5. Selected structural parameters of $[K(crypt\text{-}222)][(Cp^*{}_2Dy)_2(\mu\text{-flv}^\bullet)]$ (**3-Dy**). S9
Figure S1. Unit cell of $[(Cp^*{}_2Dy)_2(\mu\text{-flv}^\bullet)][Al(OC\{CF_3\}_3)_4]\cdot CH_2Cl_2$ (**1-Dy**$\cdot CH_2Cl_2$). S10
Figure S2. Unit cell of $[(Cp^*{}_2Dy)_2(\mu\text{-flv})]\cdot 2THF$ (**2-Dy**·2THF). S11
Figure S3. Unit cell of $[K(crypt\text{-}222)][(Cp^*{}_2Dy)_2(\mu\text{-flv}^\bullet)]\cdot 4THF$ (**3-Dy**·4THF). S12

2 IR Spectroscopy S13

Figure S4. FTIR spectra of $[(Cp^*{}_2Ln)_2(\mu\text{-flv}^\bullet)][Al(OC\{CF_3\}_3)_4]$ (**1-Ln**). S13
Figure S5. FTIR spectra of $[(Cp^*{}_2Ln)_2(\mu\text{-flv})]$ (**2-Ln**). S14
Figure S6. FTIR spectra of $[K(crypt\text{-}222)][(Cp^*{}_2Ln)_2(\mu\text{-flv}^\bullet)]$ (**3-Ln**). S15

3 Cyclic Voltammetry S16

Figure S7. Full cyclic voltammogram and magnification of $[(Cp^*{}_2Dy)_2(\mu\text{-flv}^\bullet)][Al(OC\{CF_3\}_3)_4]$ (**1-Dy**). S16
Figure S8. Full cyclic voltammogram and magnification of $(Cp^*{}_2Dy)_2(\mu\text{-flv})$ (**2-Dy**). S16
Figure S9. Full cyclic voltammogram and magnification of $[K(crypt\text{-}222)][(Cp^*{}_2Dy)_2(\mu\text{-flv}^\bullet)]$ (**3-Dy**). S17

4 Magnetic Measurements S18

Figure S10. Variable-temperature dc magnetic susceptibility data for **1-Gd.** S18
Figure S11. Variable-temperature dc magnetic susceptibility data for **2-Gd.** S19
Figure S12. Variable-temperature dc magnetic susceptibility data for **3-Gd.** S20
Figure S13. Variable-temperature dc magnetic susceptibility data for **1-Dy.** S21
Figure S14. Variable-temperature dc magnetic susceptibility data for **2-Dy.** S21
Figure S15. Variable-temperature dc magnetic susceptibility data for **2-Gd** and **2-Dy** with fit. S22
Figure S16. Variable-temperature dc magnetic susceptibility data for **3-Dy.** S22

Table S6. Summary of fitting parameters *J* (cm^{-1}) and *g* for fits of the $\chi_M T$ vs. *T* data of **1-Gd** – **3-Gd** at 0.1 T and 1.0 T. S23
Figure S17. Variable-temperature dc magnetic susceptibility data for restrained polycrystalline samples of **1-Dy**, and **3-Dy**, collected under a 0.1 T applied dc field. S23
Figure S18. Variable-temperature dc magnetic susceptibility data for restrained polycrystalline samples of **1-Dy**, **2-Dy**, and **3-Dy**, collected under a 0.1 T applied dc field. S24
Figure S19. Cole-Cole (Argand) plots for ac susceptibility collected under zero applied dc field for **1-Dy**. S24
Figure S20. Variable-temperature, variable-frequency in-phase (χ_M') and out-of-phase (χ_M") ac magnetic susceptibility data for **2-Dy.** S25
Figure S21. Cole-Cole (Argand) plots for ac susceptibility collected under zero applied dc field for **2-Dy**. S26
Figure S22. Cole-Cole (Argand) plots for ac susceptibility collected under zero applied dc field for **3-Dy**. S26
Figure S23. Plot of magnetization vs. time used to derive relaxation times for **1-Dy** at 1.8 K and 1.85 K. S27
Figure S24. Plot of magnetization vs. time used to derive relaxation times for **1-Dy** at 1.90 K and 1.95 K. S27
Figure S25. Plot of magnetization vs. time used to derive relaxation times for **1-Dy** at 2.00 K and 2.10 K. S28
Figure S26. Plot of magnetization vs. time used to derive relaxation times for **1-Dy** at 2.20 K and 2.30 K. S28
Figure S27. Plot of magnetization vs. time used to derive relaxation times for **1-Dy** at 2.40 K and 2.50 K. S29
Figure S28. Plot of magnetization vs. time used to derive relaxation times for **3-Dy** at 1.8 K and 1.9 K. S29
Figure S29. Plot of magnetization vs. time used to derive relaxation times for **3-Dy** at 2.0 K and 2.25 K. S30
Figure S30. Plot of magnetization vs. time used to derive relaxation times for **3-Dy** at 2.5 K and 3.0 K. S30
Figure S31. Plot of magnetization vs. time used to derive relaxation times for **3-Dy** at 3.5 K and 4.0 K. S31
Figure S32. Plot of magnetization vs. time used to derive relaxation times for **3-Dy** at 4.5 K and 5.0 K. S31
Figure S33. Plot of magnetization vs. time used to derive relaxation times for **3-Dy** at 5.5 K and 6.0 K. S32
Figure S34. Plot of magnetization vs. time used to derive relaxation times for **3-Dy** at 6.5 K and 7.0 K. S32
Figure S35. Plot of magnetization vs. time used to derive relaxation times for **3-Dy** at 7.5 K and 8.0 K. S33
Figure S36. Plot of magnetization vs. time used to derive relaxation times for **3-Dy** at 8.5 K and 9.0 K. S33
Figure S37. Arrhenius plot of relaxation time data for **1-Dy** obtained between 2.9 and 7.5 K with fit to an Orbach process. S34

Figure S38. Arrhenius plot of relaxation time data for **1-Dy** obtained between 1.8 and 7.5 K with fit to an Orbach process. S34
Figure S39. Individual contributions of the multiple magnetic relaxation pathways to the Arrhenius plot of **1-Dy**, with fit to an Orbach and a Raman process (freely refined Orbach parameters). S35
Figure S40. Individual contributions of the multiple magnetic relaxation pathways to the Arrhenius plot of **1-Dy**, with fit to an Orbach and a Raman process (fixed Orbach parameters). S36
Figure S41. Plot of natural log of the relaxation time versus the inverse temperature for **2-Dy**. S36
Figure S42. Arrhenius plot of relaxation time data for **3-Dy** from 10 to 16 K with fits to two Orbach relaxation mechanisms. S37
Figure S43. Individual contributions of the multiple magnetic relaxation pathways to the Arrhenius plot of **3-Dy**, with fit to an Orbach, a Raman and a Quantum tunneling process. S38
Figure S44. Plot of natural log of the relaxation time versus the inverse temperature for **1-Dy**, for **2-Dy**, and for **3-Dy**. S39
Table S7. Relaxation times of **1-Dy**, **2-Dy**, and **3-Dy** at various temperatures. S39
Table S8. Best-Fit Parameters for the Arrhenius plots of **1-Dy**, **2-Dy**, and **3-Dy**. S41
Figure S45. Plot of magnetization (*M*) vs dc magnetic field (*H*) for **1-Dy**. S41
Figure S46. Plot of magnetization (*M*) vs. dc magnetic field (*H*) for **2-Dy.** S42
Figure S47. Plot of magnetization (*M*) vs dc magnetic field (*H*) for **3-Dy**. S42
Figure S48. Plot of magnetization (*M*) vs. dc magnetic field (*H*) at an average sweep rate of 0.01 T/s for **3-Dy** at 10 K. S43
Figure S49. Magnified plot of magnetization (*M*) vs. dc magnetic field (*H*) at an average sweep rate of 0.01 T/s for **3-Dy** from 2 to 10 K. S43
Figure S50. Plot of magnetization (*M*) vs. dc magnetic field (*H*) at an average sweep rate of 0.01 T/s for **1-Dy**, **2-Dy**, and **3-Dy** at 2 K. S44
Figure S51. Field-dependent magnetization and reduced magnetization data for **1-Gd**, collected from 0 to 7 T between 2 K and 10 K. S44
Figure S52. Field-dependent magnetization and reduced magnetization data for **1-Dy**, collected from 0 to 7 T between 2 K and 10 K. S45
Figure S53. Field-dependent magnetization and reduced magnetization data for **2-Gd**, collected from 0 to 7 T between 2 K and 10 K. S45
Figure S54. Field-dependent magnetization and reduced magnetization data for **2-Dy**, collected from 0 to 7 T between 2 K and 10 K. S46
Figure S55. Field-dependent magnetization and reduced magnetization data for **3-Gd**, collected from 0 to 7 T between 2 K and 10 K. S46
Figure S56. Field-dependent magnetization and reduced magnetization data for **3-Dy**, collected from 0 to 7 T between 2 K and 10 K. S47
Figure S57. First derivative plots of the fits for the reverse sweep of the magnetic hysteresis loops of **1-Dy** from 2 K to 3 K and **3-Dy** from 2 K to 9.5 K. S47
Figure S58. Temperature dependence of the demagnetization processes of **1-Dy** through Cauchy probability distribution function analysis. S48

Table S9. Cauchy probability distribution function analyses data of demagnetization processes of **1-Dy** from 2 K to 3 K. S49
Figure S59. Temperature dependence of the demagnetization processes of **3-Dy** through Cauchy probability distribution function analysis. S50
Table S10. Cauchy probability distribution function analyses data of demagnetization processes of **3-Dy** from 2 K to 9.5 K. S51

5 TD-DFT Calculations S52

Table S11. Majority contributions of the TD-DFT-calculated transition states for **1-Gd.** S52
Table S12. Majority contributions of the TD-DFT-calculated transition states for **2-Gd.** S54
Table S13. Majority contributions of the TD-DFT-calculated transition states for **3-Gd.** S56
Figure S60. Frontier molecular orbital plots of **1-Gd**, **2-Gd**, and **3-Gd**. S57

6 Broken-Symmetry DFT S58

Table S14. Results of the broken-symmetry DFT calculations performed on the crystal coordinates of $[(Cp^*{}_2Gd)_2(\mu\text{-flv}^{\bullet})]^+$ in **1-Gd**, using multiple functionals. S58
Table S15. Results of the broken-symmetry DFT calculations performed on $[(Cp^*{}_2Gd)_2(\mu\text{-flv})]$, **2-Gd**, using multiple functionals. S59
Table S16. Results of the broken-symmetry DFT calculations performed on the crystal coordinates of $[(Cp^*{}_2Gd)_2(\mu\text{-flv}^{\bullet})]^-$ in **3-Gd**, using multiple functionals. S59
Table S17. Mulliken spin populations on the N atoms in flv ligands for $[(Cp^*{}_2Gd)_2(\mu\text{-flv}^{\bullet})]^+$ in **1-Gd**, and $[(Cp^*{}_2Gd)_2(\mu\text{-flv}^{\bullet})]^-$ in **3-Gd**. S60

7 High-field Electron Paramagnetic Resonance S60

Figure S61. Temperature-dependent powder EPR spectra collected for **1-Gd** at frequencies of 52 GHz and 385 GHz. S60
Figure S62. Temperature-dependent powder EPR spectra collected for **2-Gd** at frequencies of 52 GHz and 385 GHz. S61
Table S18. Comparison of spin-Hamiltonian parameters deduced from EPR for all samples. S62

8 Coordinates of Optimized Structures S63

9 Python Scripts for Magnetic Hysteresis Analyses S72

10 References S83

1 Single Crystal X-ray Diffraction

Table S1. Crystallographic data and structural refinement of $[(Cp^*_2Ln)_2(\mu\text{-flv}^\bullet)][Al(OC\{CF_3\}_3)_4]$ (**1-Ln**) and $[(Cp^*_2Ln)_2(\mu\text{-flv})]$ (**2-Ln**), where Ln = Gd and Dy. **1-Ln** crystallized with one dichloromethane (DCM) molecule in the lattice. **2-Ln** crystallized with two tetrahydrofuran (THF) molecules in the lattice.

Compound	**1-Gd**·DCM	**1-Dy**·DCM	**2-Gd**·2THF	**2-Dy**·2THF
CCDC no.	2455654	2455658	2455655	2455653
Empirical formula	$C_{71}H_{70}AlCl_2F_{36}Gd_2N_4O_4$	$C_{71}H_{70}AlCl_2Dy_2F_{36}N_4O_4$	$C_{62}H_{84}Gd_2N_4O_2$	$C_{62}H_{84}Dy_2N_4O_2$
Formula weight	2139.69	2150.19	1231.83	1242.33
Temperature/K	100.15	100.15	99.99(10)	220.00(10)
Crystal system	monoclinic	monoclinic	triclinic	triclinic
Space group	*I*2/a	*I*2/a	*P*-1	*P*-1
a (Å)	17.0204(2)	16.97710(10)	9.8365(2)	9.99090(10)
b (Å)	17.8704(3)	17.86390(10)	10.0153(2)	10.1412(2)
c (Å)	26.1664(4)	26.1643(2)	14.2526(2)	14.2032(2)
α (°)	90	90	96.7940(10)	96.7480(10)
β (°)	95.5920(10)	95.7730(10)	97.9930(10)	97.1630(10)
γ (°)	90	90	93.1730(10)	94.0660(10)
Volume (Å^3)	7920.9(2)	7894.79(9)	1377.01(4)	1412.59(4)
Z	4	4	1	1
ρ_{calc} (g/cm^3)	1.794	1.809	1.485	1.460
μ (mm^{-1})	12.673	11.976	15.754	14.323
F(000)	4220.0	4236.0	628.0	632.0
Crystal size (mm^3)	0.13 × 0.1 × 0.07	0.18 × 0.13 × 0.11	0.171 × 0.169 × 0.126	0.312 × 0.234 × 0.048
Radiation	CuK_α (λ = 1.54184)	CuK_α (λ = 1.54184)	CuK_α (λ = 1.54184)	CuK_α (λ = 1.54184)
2Θ range for data collection (°)	5.998 to 155.348	6 to 160.14	6.314 to 160.12	6.324 to 160.394
Index ranges	$-18 \le h \le 21$, $-21 \le k \le 22$, $-33 \le l \le 33$	$-21 \le h \le 21$, $-15 \le k \le 22$, $-33 \le l \le 33$	$-12 \le h \le 12$, $-12 \le k \le 12$, $-15 \le l \le 18$	$-12 \le h \le 12$, $-12 \le k \le 12$, $-18 \le l \le 17$
Reflections collected	33675	75451	25094	24304
Independent reflections	8243 [R_{int} = 0.0408, R_σ = 0.0332]	8586 [R_{int} = 0.0450, R_σ = 0.0262]	5964 [R_{int} = 0.0459, R_σ = 0.0382]	6102 [R_{int} = 0.0517, R_σ = 0.0522]
Data/restraints/ parameters	8243/557/801	8586/1410/801	5964/0/326	6102/0/344
Goodness-of-fit on F^2	1.051	1.074	1.124	1.057
Final *R* indexes (*I* > = 2 σ (*I*))	R_1 = 0.0400, wR_2 = 0.0988	R_1 = 0.0386, wR_2 = 0.1018	R_1 = 0.0320, wR_2 = 0.0846	R_1 = 0.0400, wR_2 = 0.0988
Final *R* indexes (all data)	R_1 = 0.0453, wR_2 = 0.1019	R_1 = 0.0404, wR_2 = 0.1031	R_1 = 0.0328, wR_2 = 0.0852	R_1 = 0.0411, wR_2 = 0.1000
Largest diff. peak/hole (e Å^{-3})	0.86/-0.78	0.84/-0.55	1.03/-1.11	2.52/-1.08

Table S2. Crystallographic data and structural refinement of [K(crypt-222)][(Cp*$_2$Ln)$_2$(μ-flv$^•$)] (**3-Ln**), where Ln = Gd and Dy. **3-Ln** crystallized with four THF molecules in the lattice.

Compound	**3-Gd**·4THF	**3-Dy**·4THF
CCDC no.	2455656	2455657
Empirical formula	$C_{88}H_{136}Gd_2KN_6O_{10}$	$C_{88}H_{136}Dy_2KN_6O_{10}$
Formula weight	1791.62	1802.12
Temperature/K	100.00(13)	99.99(10)
Crystal system	triclinic	triclinic
Space group	*P*-1	*P*-1
a (Å)	12.2242(2)	12.23530(10)
b (Å)	13.4971(2)	13.50360(10)
c (Å)	26.6315(4)	26.5510(2)
α (°)	87.1860(10)	86.9860(10)
β (°)	89.8080(10)	89.7190(10)
γ (°)	79.1930(10)	78.6710(10)
Volume (Å^3)	4310.75(12)	4295.28(6)
Z	2	2
ρ_{calc} (g/cm^3)	1.380	1.393
μ (mm^{-1})	10.740	10.098
F(000)	1866.0	1874.0
Crystal size (mm^3)	0.309 × 0.196 × 0.118	0.207 × 0.142 × 0.14
Radiation	CuK$_\alpha$ (λ = 1.54184)	CuK$_\alpha$ (λ = 1.54184)
2Θ range for data collection (°)	6.646 to 156.054	6.668 to 160.34
Index ranges	$-15 \le h \le 15$, $-16 \le k \le 17$, $-33 \le l \le 32$	$-15 \le h \le 15$, $-17 \le k \le 17$, $-33 \le l \le 31$
Reflections collected	74076	102022
Independent reflections	17912 [R_{int} = 0.0404, R_σ = 0.0342]	18473 [R_{int} = 0.0388, R_σ = 0.0286]
Data/restraints/ parameters	17912/0/984	18473/0/984
Goodness-of-fit on F^2	1.063	1.109
Final *R* indexes (I > = 2 σ (I))	R_1 = 0.0457, wR_2 = 0.1189	R_1 = 0.0351, wR_2 = 0.0910
Final *R* indexes (all data)	R_1 = 0.0502, wR_2 = 0.1216	R_1 = 0.0371, wR_2 = 0.0921
Largest diff. peak/hole (e Å^{-3})	2.18/-1.06	0.93/-1.01

Table S3. Selected structural parameters of [(Cp*$_2$Ln)$_2$(μ-flv$^•$)][Al(OC{CF$_3$}$_3$)$_4$] (**1-Ln**) and [(Cp*$_2$Ln)$_2$(μ-flv)] (**2-Ln**), where Ln = Gd and Dy.

**1-Gd**		**1-Dy**		**2-Gd**		**2-Dy**	
**Atoms**	**Distance (Å)**	**Atoms**	**Distance (Å)**	**Atoms**	**Distance (Å)**	**Atoms**	**Distance (Å)**
**Central C–C**							
C1–C1'	1.430(7)	C1–C1'	1.426(6)	C1–C1'	1.454(5)	C1–C1'	1.454(5)
**N–C$_{central}$**							
N1–C1	1.344(5)	N1–C1	1.343(4)	N1–C1	1.344(4)	N1–C1	1.345(4)
N2–C1	1.348(5)	N2–C1	1.348(4)	N2–C1	1.343(3)	N2–C1	1.337(4)
N1'–C1'	1.344(5)	N1'–C1'	1.343(4)	N1'–C1'	1.344(4)	N1'–C1'	1.345(4)
N2'–C1'	1.348(5)	N2'–C1'	1.348(4)	N2'–C1'	1.343(3)	N2'–C1'	1.337(4)
**N–C$_{peripheral}$**							
N1–C5	1.381(5)	N1–C3	1.377(4)	N1–C2	1.392(3)	N1–C5	1.393(4)
N2–C2	1.367(5)	N2–C2	1.369(4)	N2–C7	1.401(3)	N2–C2	1.389(4)
N1'–C5'	1.381(5)	N2'–C5'	1.377(4)	N1'–C2'	1.392(3)	N1'–C5'	1.393(4)
N2'–C2'	1.367(5)	N2'–C2'	1.369(4)	N2'–C7'	1.401(3)	N2'–C2'	1.389(4)
**Phenyl C–C**							
C2–C3	1.421(5)	C2–C7	1.418(4)	C2–C3	1.403(4)	C2–C3	1.400(4)
C3–C4	1.359(6)	C7–C6	1.364(5)	C3–C4	1.391(4)	C3–C4	1.390(4)
C4–C7	1.416(6)	C6–C5	1.409(5)	C4–C5	1.393(4)	C4–C7	1.385(6)
C7–C6	1.368(6)	C5–C4	1.373(5)	C5–C6	1.388(4)	C7–C6	1.385(5)
C6–C5	1.401(5)	C4–C3	1.406(5)	C6–C7	1.400(4)	C6–C5	1.402(4)
C5–C2	1.425(5)	C3–C2	1.429(5)	C7–C2	1.413(4)	C5–C2	1.413(4)
C2'–C3'	1.421(5)	C2'–C7'	1.418(4)	C2'–C3'	1.403(4)	C2'–C3'	1.400(4)
C3'–C4'	1.359(6)	C7'–C6'	1.364(5)	C3'–C4'	1.391(4)	C3'–C4'	1.390(4)
C4'–C7'	1.416(6)	C6'–C5'	1.409(5)	C4'–C5'	1.393(4)	C4'–C7'	1.385(6)
C7'–C6'	1.368(6)	C5'–C4'	1.373(5)	C5'–C6'	1.388(4)	C7'–C6'	1.385(5)
C6'–C5'	1.401(5)	C4'–C3'	1.406(5)	C6'–C7'	1.400(4)	C6'–C5'	1.402(4)
C5'–C2'	1.425(5)	C3'–C2'	1.429(5)	C7'–C2'	1.413(4)	C5'–C2'	1.413(4)
**Ln–N**							
Gd1–N1	2.468(3)	Dy1–N1	2.442(3)	Gd1–N1	2.416(2)	Dy1–N1	2.398(2)
Gd1–N2	2.474(3)	Dy1–N2	2.449(3)	Gd1–N2	2.416(2)	Dy1–N2	2.399(2)
Gd1'–N1'	2.468(3)	Dy1'–N1'	2.442(3)	Gd1'–N1'	2.416(2)	Dy1'–N1'	2.398(2)
Gd1'–N2'	2.474(3)	Dy1'–N2'	2.449(3)	Gd1'–N2'	2.416(2)	Dy1'–N2'	2.399(2)
**Ln–Ln**							
Gd1–Gd1'	7.226(1)	Dy1–Dy1'	7.167(1)	Gd1–Gd1'	7.105(1)	Dy1–Dy1'	7.052(1)
**Objects**	**Angles (°)**	**Objects**	**Angles (°)**	**Objects**	**Angles (°)**	**Objects**	**Angles (°)**
(Ph–Ph)1	0.1(1)	(Ph–Ph)1	0.1(1)	(Ph–Ph)1	0.1(1)	(Ph–Ph)1	0.1(1)
Gd1–N1–N2'–Gd1'	7.1(5)	Dy1–N1–N2'–Dy1'	-5.9(5)	Gd1–N1–N2'–Gd1'	17.5(4)	Dy1–N1–N2'–Dy1'	16.0(4)
Gd1–N2–N1'–Gd1'	-7.1(5)	Dy1–N2–N1'–Dy1'	5.9(5)	Gd1–N2–N1'–Gd1'	-17.5(4)	Dy1-N2-N4-Dy2	-16.0(4)

Cnt–Gd1-Cnt	142.1	Cnt–Dy1-Cnt	142.3	Cnt–Gd1-Cnt	141.7	Cnt–Dy1-Cnt	140.8
Cnt–Gd1'-Cnt	142.1	Cnt–Dy1'-Cnt	142.3	Cnt–Gd1'-Cnt	141.7	Cnt–Dy2-Cnt	140.8

Table S4. Selected structural parameters of [K(crypt-222)][$(Cp^*_2Gd)_2$(μ-flv$^•$)] (**3-Gd**).

3-Gd			
**Atoms**	**Distance (Å)**	**Atoms**	**Distance (Å)**
**Central C–C**		**Phenyl C–C continued**	
C1–C1'	1.385(7)	C29–C34	1.400(5)
C28–C28'	1.388(7)	C34–C33	1.396(5)
**N–C$_{central}$**		C33–C32	1.381(6)
N1–C1	1.377(4)	C32–C31	1.397(6)
N2–C1	1.386(4)	C31–C30	1.401(5)
N1'–C1'	1.377(4)	C30–C29	1.441(5)
N2'–C1'	1.386(4)	C29'–C34'	1.400(5)
N3–C28	1.376(5)	C34'–C33'	1.396(5)
N4–C28	1.387(5)	C33'–C32'	1.381(6)
N3'–C28'	1.376(5)	C32'–C31'	1.397(6)
N4'–C28'	1.387(5)	C31'–C30'	1.401(5)
**N–C$_{peripheral}$**		C30'–C29'	1.441(5)
N1–C4	1.394(5)	**Gd–N**	
N2–C2	1.391(4)	Gd1–N1	2.350(3)
N1'–C'	1.394(5)	Gd1–N2	2.331(3)
N2'–C'	1.391(4)	Gd1'–N1'	2.350(3)
N3–C29	1.390(5)	Gd1'–N2'	2.331(3)
N4–C30	1.383(5)	Gd2–N3	2.355(3)
N3'–C29'	1.390(5)	Gd2–N4	2.356(3)
N4'–C30'	1.383(5)	Gd2'–N3'	2.355(3)
**Phenyl C–C**		Gd2'–N4'	2.356(3)
C2–C3	1.406(5)	**Gd–Gd**	
C3–C7	1.395(5)	Gd1–Gd1'	6.938(1)
C7–C6	1.387(6)	Gd2–Gd2'	6.961(1)
C6–C5	1.391(5)	**Objects**	**Angles (°)**
C5–C4	1.402(5)	(Ph–Ph)1	0.1(1)
C4–C2	1.419(5)	(Ph–Ph)2	0.1(1)
C2'–C3'	1.406(5)	Gd1–N1–N2'–Gd1'	15.6(5)
C3'–C7'	1.395(5)	Gd1–N2–N1'–Gd1'	-15.6(5)
C7'–C6'	1.387(6)	Gd2–N3–N4'–Gd2'	-25.8(5)
C6'–C5'	1.391(5)	Gd2–N4–N3'–Gd2'	25.8(5)
C5'–C4'	1.402(5)	Cnt–Gd1-Cnt	140.4
C4'–C2'	1.419(5)	Cnt–Gd1'-Cnt	140.4

		Cnt–Gd2-Cnt	137.9
		Cnt–Gd2'-Cnt	137.9

Table S5. Selected structural parameters of [K(crypt-222)][($Cp^*_2Dy)_2$(μ-flv$^•$)] (**3-Dy**).

**3-Dy**			
**Atoms**	**Distance (Å)**	**Atoms**	**Distance (Å)**
**Central C–C**		**Phenyl C–C continued**	
C1–C1'	1.375(5)	C29–C30	1.401(4)
C28–C28'	1.378(5)	C30–C34	1.397(4)
**N–C$_{central}$**		C34–C33	1.391(4)
N1–C1	1.388(3)	C33–C32	1.393(4)
N2–C1	1.378(3)	C32–C31	1.400(4)
N1'–C1'	1.388(3)	C31–C29	1.423(4)
N2'–C1'	1.378(3)	C29'–C30'	1.401(4)
N3–C28	1.382(3)	C30'–C34'	1.397(4)
N4–C28	1.379(3)	C34'–C33'	1.391(4)
N3'–C28'	1.382(3)	C33'–C32'	1.393(4)
N4'–C28'	1.379(3)	C32'–C31'	1.400(4)
**N–C$_{peripheral}$**		C31'–C29'	1.423(4)
N1–C2	1.394(3)	**Dy–N**	
N2–C3	1.396(3)	Dy1–N1	2.330(2)
N1'–C2'	1.394(3)	Dy1–N2	2.323(2)
N2'–C3'	1.396(3)	Dy1'–N1'	2.330(2)
N3–C31	1.391(3)	Dy1'–N2'	2.323(2)
N4–C29	1.399(3)	Dy2–N3	2.309(2)
N3'–C31'	1.391(3)	Dy2–N4	2.322(2)
N4'–C29'	1.399(3)	Dy2'–N3'	2.309(2)
**Phenyl C–C**		Dy2'–N4'	2.322(2)
C2–C7	1.405(4)	**Dy–Dy**	
C7–C6	1.395(4)	Dy1–Dy1'	6.911(1)
C6–C5	1.386(4)	Dy2–Dy2'	6.886(1)
C5–C4	1.393(4)	**Objects**	**Angles (°)**
C4–C3	1.401(4)	(Ph–Ph)1	0.1(1)
C3–C2	1.428(4)	(Ph–Ph)2	0.1(1)
C2'–C7'	1.405(4)	Dy1–N1–N2'–Dy1'	21.4(4)
C7'–C6'	1.395(4)	Dy1–N2–N1'–Dy1'	-21.4(4)
C5'–C5'	1.386(4)	Dy2–N3–N4'–Dy2'	11.9(4)
C5'–C4'	1.393(4)	Dy2–N4–N3'–Dy2'	-11.9(4)
C4'–C3'	1.401(4)	Cnt–Dy1-Cnt	138.0
C3'–C2'	1.428(4)	Cnt–Dy1'-Cnt	138.0
		Cnt–Dy2-Cnt	140.2
		Cnt–Dy2'-Cnt	140.2

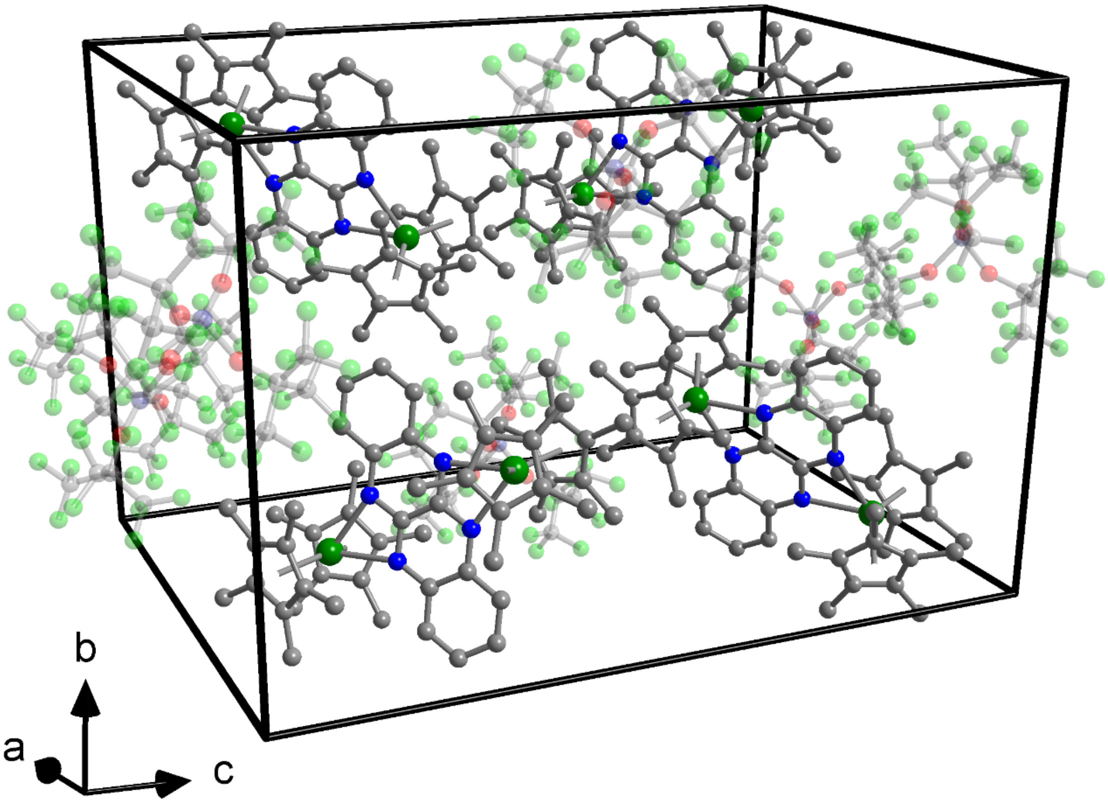

Figure S1. Unit cell of [(Cp*$_2$Dy)$_2$(μ-flv$^•$)][Al(OC{CF$_3$}$_3$)$_4$]·CH$_2$Cl$_2$ (**1-Dy**·CH$_2$Cl$_2$). Dark green, blue, red, dark blue, green, and gray spheres represent dysprosium, nitrogen, oxygen, aluminum, fluorine, and carbon atoms, respectively. All hydrogen atoms and solvent molecules are omitted, and the counter ions [Al(OC{CF$_3$}$_3$)$_4$]$^-$ are faded for clarity. **1-Gd** and **1-Dy** are isostructural.

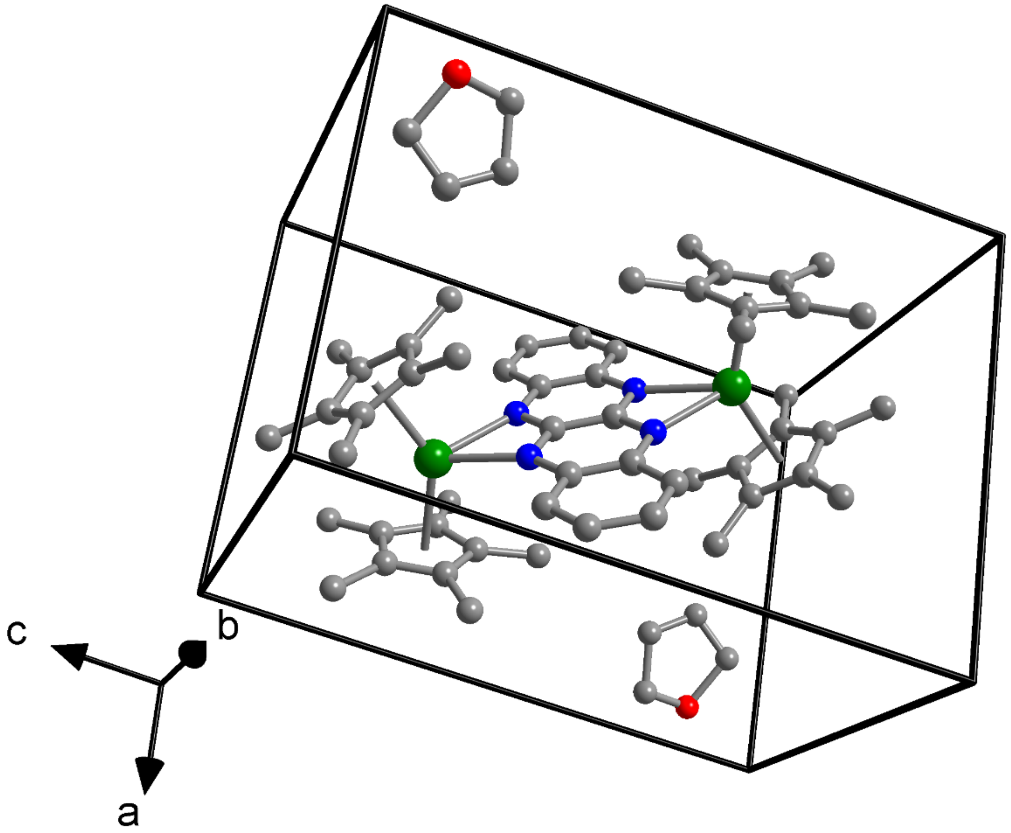

Figure S2. Unit cell of [(Cp*$_2$Dy)$_2$(μ-flv)]·2THF (**2-Dy**·2THF). Dark green, blue, red, and gray spheres represent dysprosium, nitrogen, oxygen, and carbon atoms, respectively. All hydrogen atoms are omitted for clarity. **2-Gd** and **2-Dy** are isostructural.

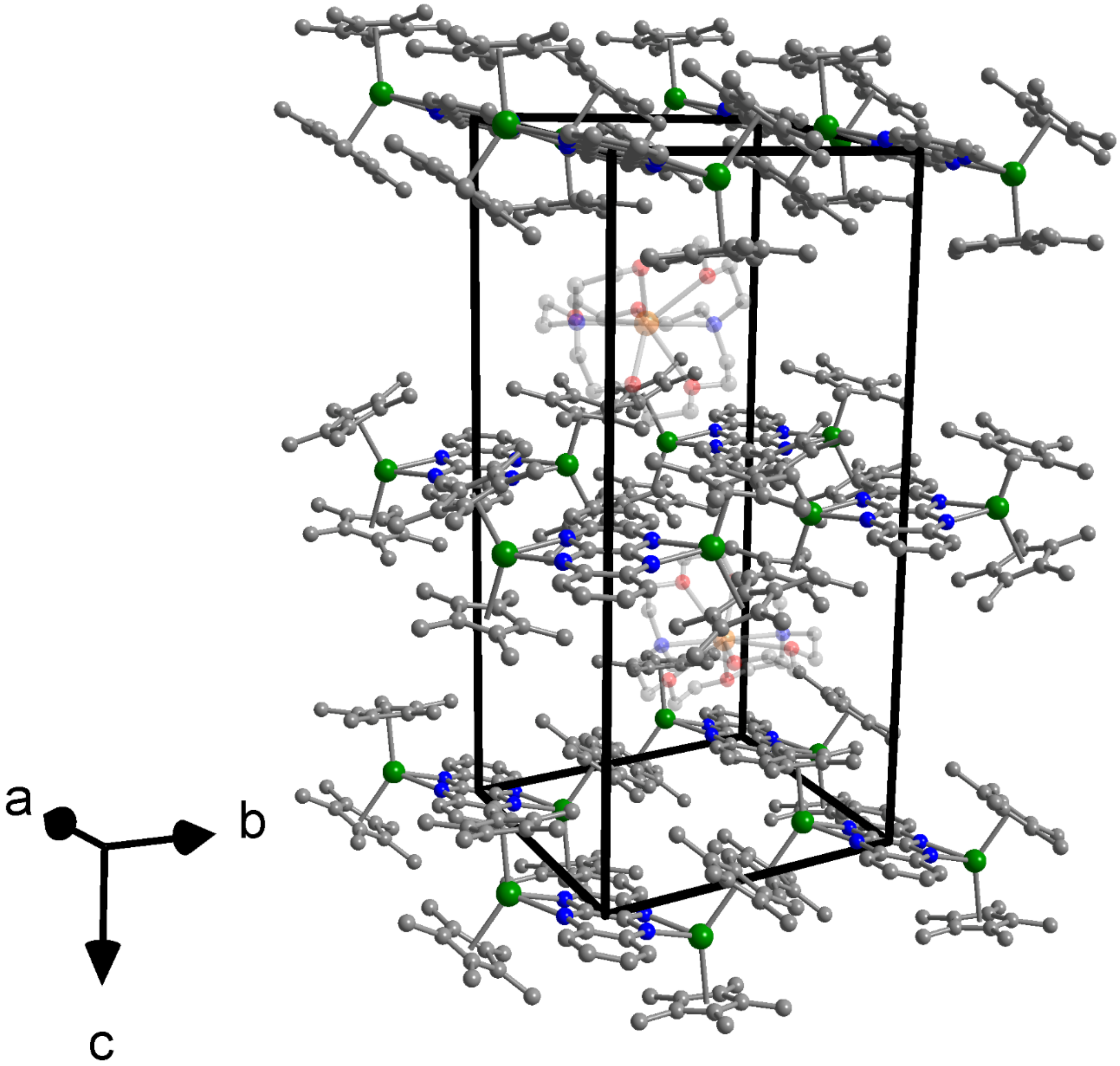

Figure S3. Unit cell of [K(crypt-222)][(Cp*$_2$Dy)$_2$(μ-flv$^\bullet$)]·4THF (**3-Dy**·4THF). Dark green, orange, red, blue, and gray spheres represent dysprosium, potassium, oxygen, nitrogen, and carbon atoms, respectively. All hydrogen atoms and solvent molecules are omitted and the counter ions [K(crypt-222)]$^+$ are faded for clarity. **3-Gd** and **3-Dy** are isostructural.

2 IR Spectroscopy

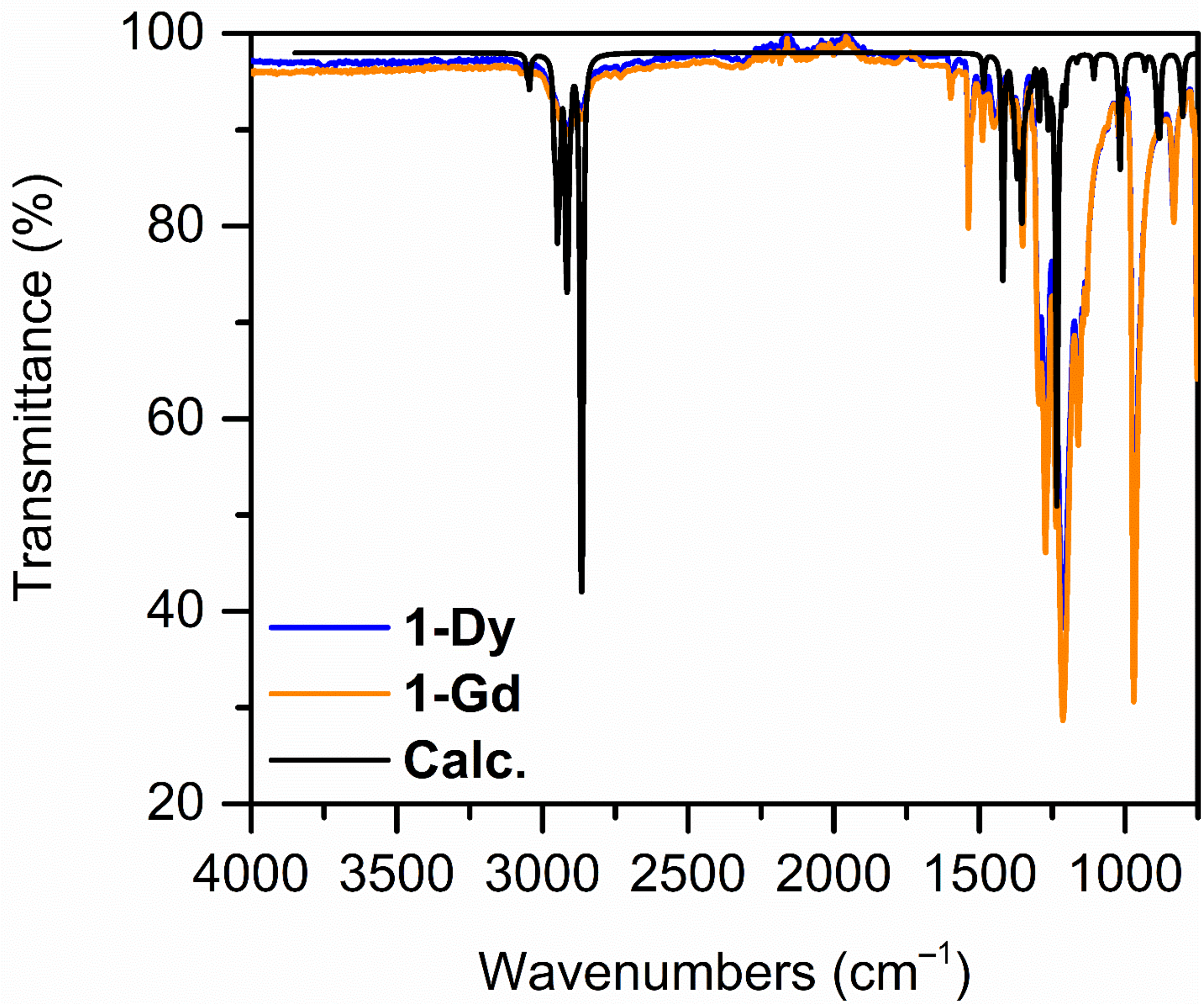

Figure S4. FTIR spectra of [(Cp*$_2$Dy)$_2$(μ-flv$^•$)][Al(OC{CF$_3$}$_3$)$_4$], (**1-Dy**, blue) and [(Cp*$_2$Gd)$_2$(μ-flv$^•$)][Al(OC{CF$_3$}$_3$)$_4$], (**1-Gd**, orange), collected on crushed crystalline solids under a nitrogen atmosphere, superimposed with the calculated spectrum for **1-Gd**, **Calc.** (black). **Calc.** represents a frequency calculation on a geometry optimized model of **1-Gd** where the counterion is omitted, [(Cp*$_2$Gd)$_2$flv$^•$]$^+$. Calculated spectra were generated through application of an 8 cm^{-1} Lorentzian line broadening. Calculated vibrations were empirically shifted by −150 cm^{-1} for a better match with the experimental spectra.

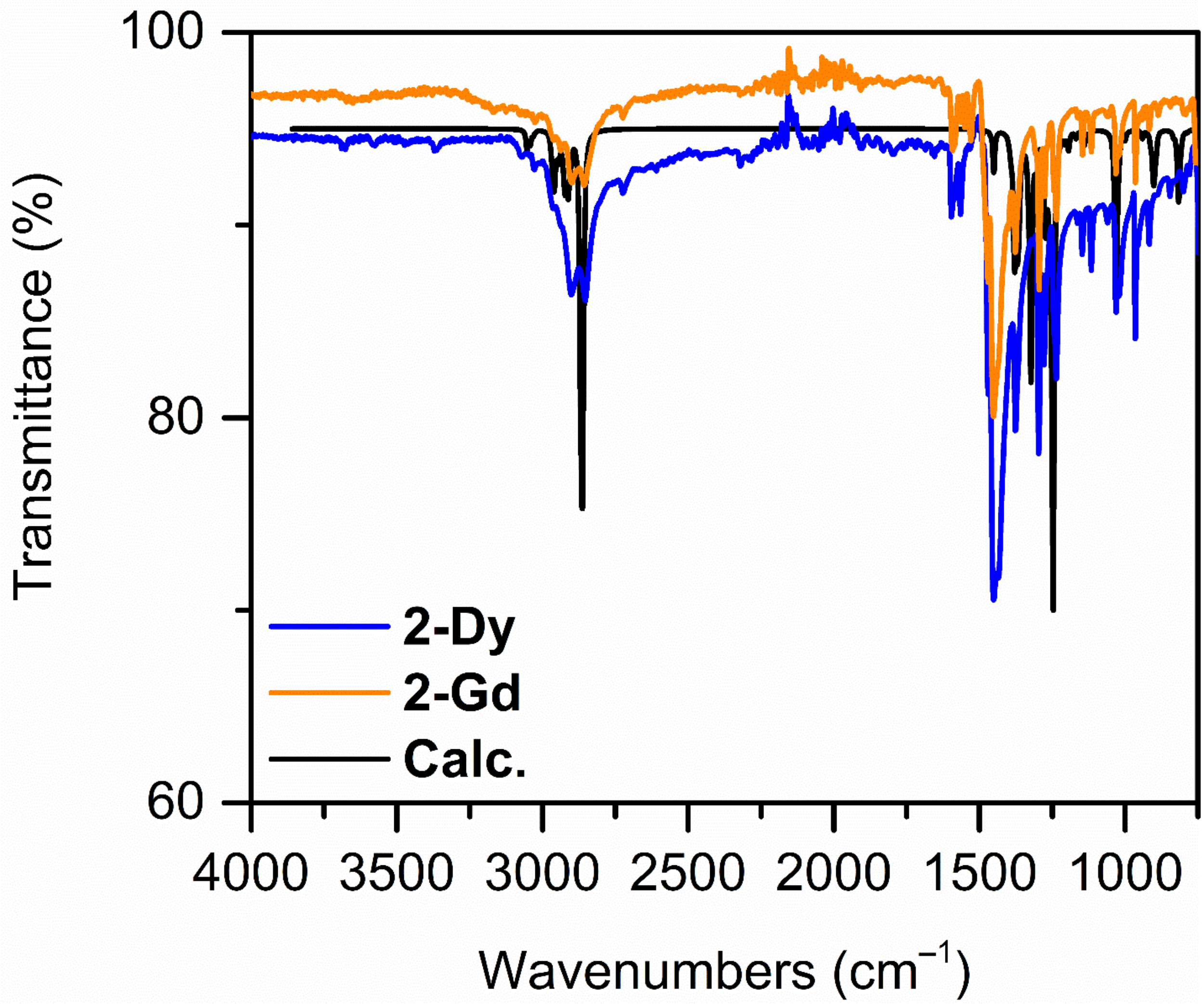

Figure S5. FTIR spectra of [(Cp*$_2$Dy)$_2$(μ-flv)], (**2-Dy**, blue) and [(Cp*$_2$Gd)$_2$(μ-flv)], (**2-Gd**, orange), collected on crushed crystalline solids under a nitrogen atmosphere, superimposed with the calculated spectrum for **2-Gd**, **Calc.** (black). **Calc.** represents a frequency calculation on a geometry optimized model of **2-Gd**. Calculated spectra were generated through application of an 8 cm^{-1} Lorentzian line broadening. Calculated vibrations were empirically shifted by −140 cm^{-1} for a better match with the experimental spectra.

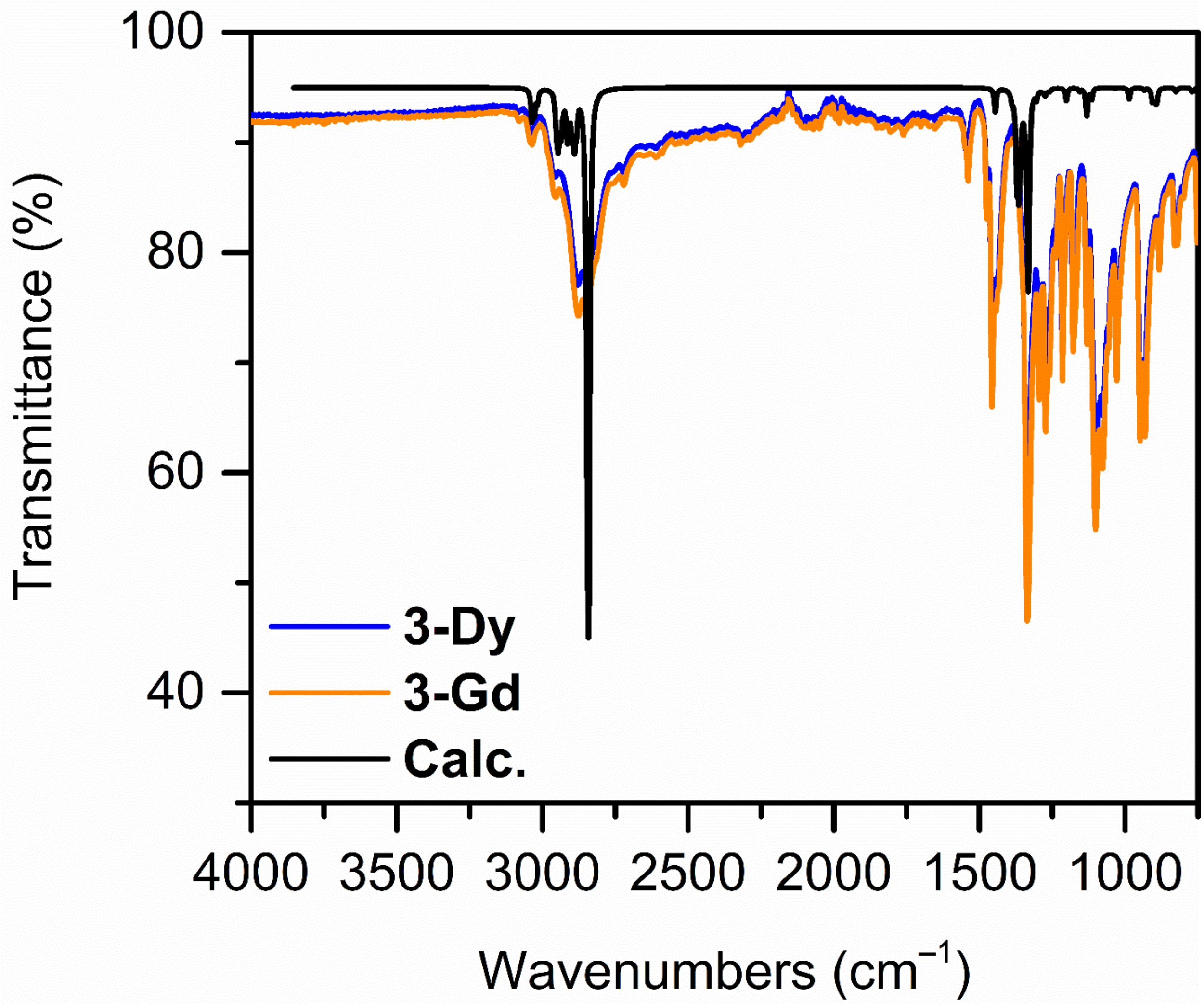

Figure S6. FTIR spectra of [K(crypt-222)][(Cp*$_2$Dy)$_2$(μ-flv$^•$)], (**3-Dy**, blue) and [K(crypt-222)][(Cp*$_2$Gd)$_2$(μ-flv$^•$)], (**3-Gd**, orange), collected on crushed crystalline solids under a nitrogen atmosphere, superimposed with the calculated spectrum for **3-Gd**, **Calc.** (black). **Calc.** represents a frequency calculation on a geometry optimized model of **3-Gd** where the counterion is omitted, [(Cp*$_2$Gd)$_2$flv$^•$]$^−$. Calculated spectra were generated through application of an 8 cm^{-1} Lorentzian line broadening. Calculated vibrations were empirically shifted by −145 cm^{-1} for a better match with the experimental spectra.

3 Cyclic Voltammetry

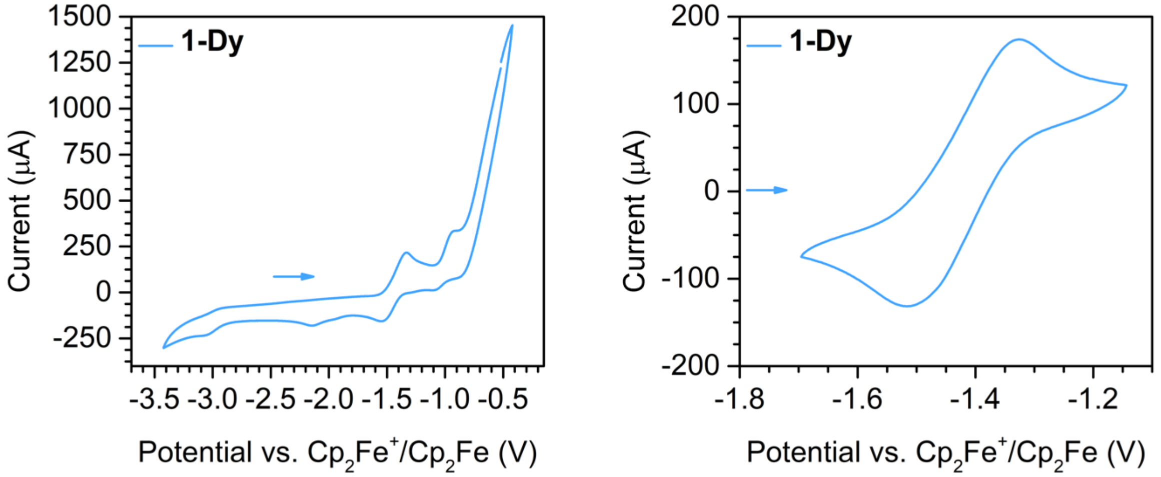

Figure S7. Full cyclic voltammogram of $[(Cp^*{}_2Dy)_2(\mu\text{-flv}^{\bullet})][Al(OC\{CF_3\}_3)_4]$ (**1-Dy**), measured in dichloromethane (left). Magnification of the quasi-reversible redox feature (right).

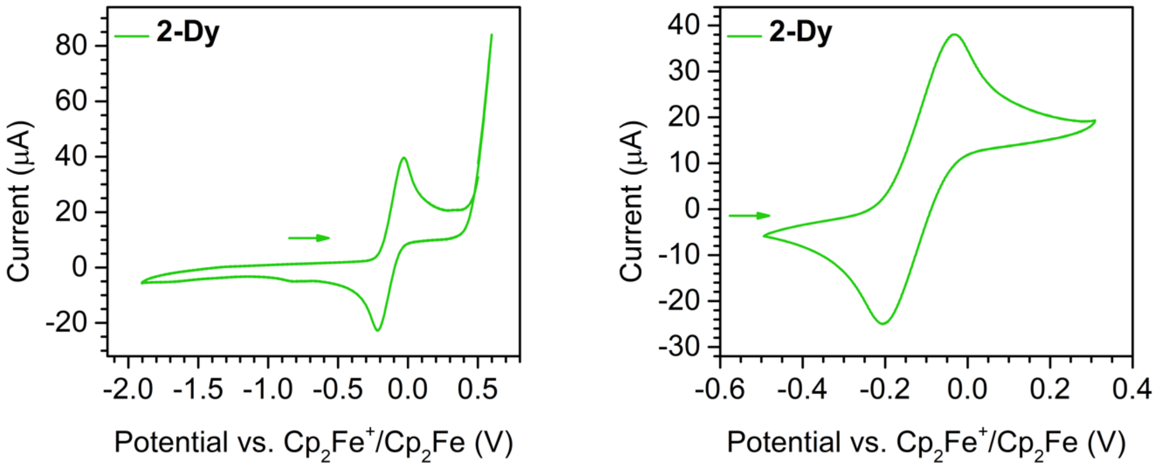

Figure S8. Full cyclic voltammogram of $[(Cp^*{}_2Dy)_2(\mu\text{-flv})]$ (**2-Dy**), measured in dichloromethane (left). Magnification of the quasi-reversible redox feature (right).

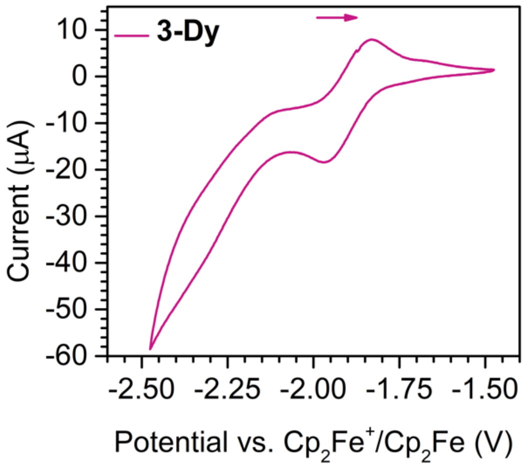

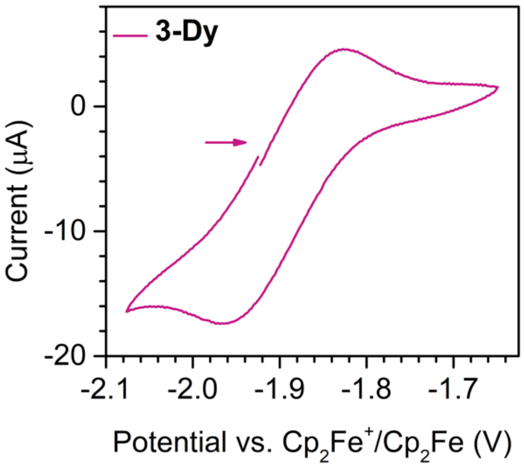

Figure S9. Full cyclic voltammogram of [K(crypt-222)][(Cp*$_2$Dy)$_2$(μ-flv$^{•}$)] (**3-Dy**), measured in THF (left). Magnification of the quasi-reversible redox feature (right).

4 Magnetic Measurements

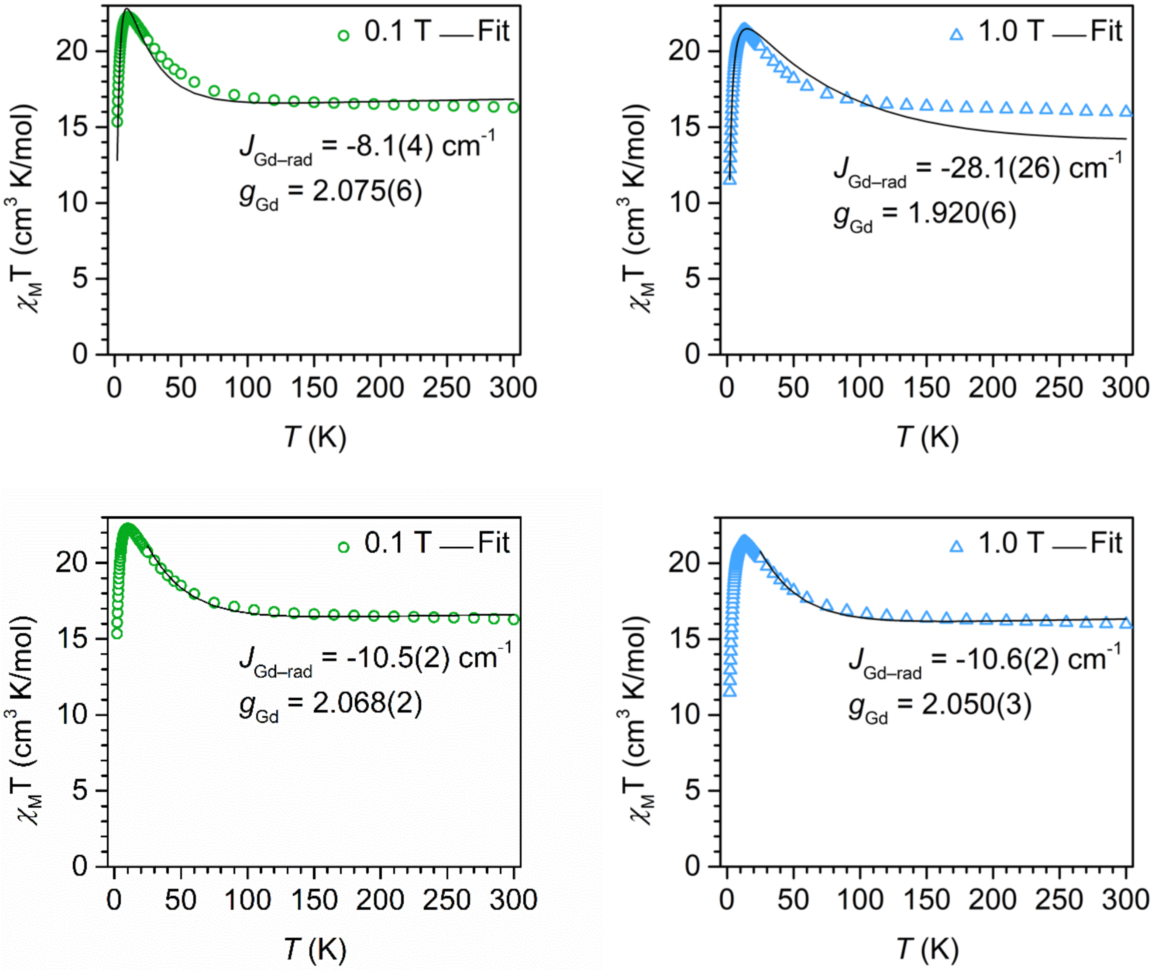

Figure S10. Variable-temperature dc magnetic susceptibility data for a restrained polycrystalline sample of **1-Gd** collected under 0.1 T (left), and 1.0 T (right) applied dc fields. The black lines represent fits to the data of **1-Gd** over the entire temperature range from 2 to 300 K (top) and from 25 to 300 K (bottom).

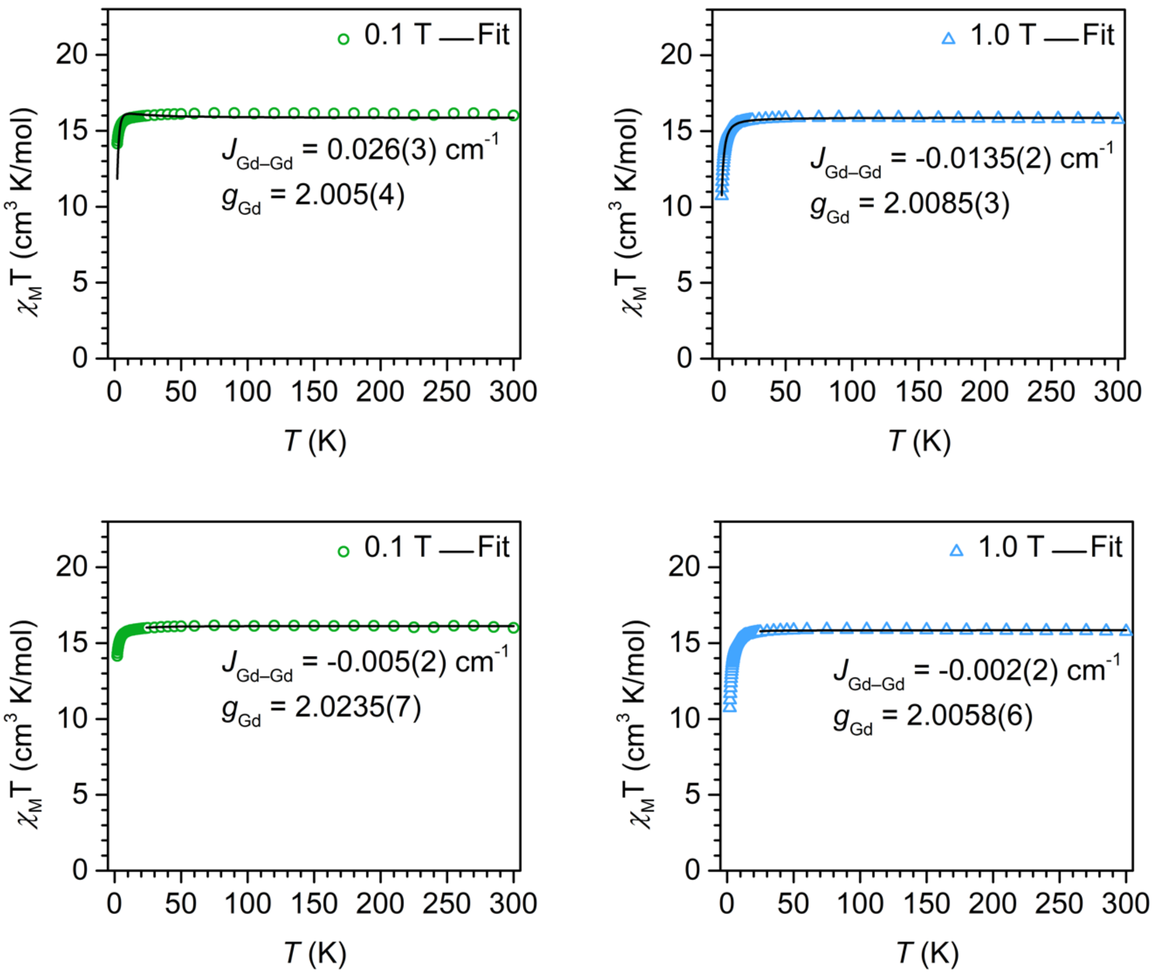

Figure S11. Variable-temperature dc magnetic susceptibility data for a restrained polycrystalline sample of **2-Gd** collected under 0.1 T (left), and 1.0 T (right) applied dc fields. The black lines represent fits to the data of **2-Gd** over the entire temperature range from 2 to 300 K (top) and from 25 to 300 K (bottom).

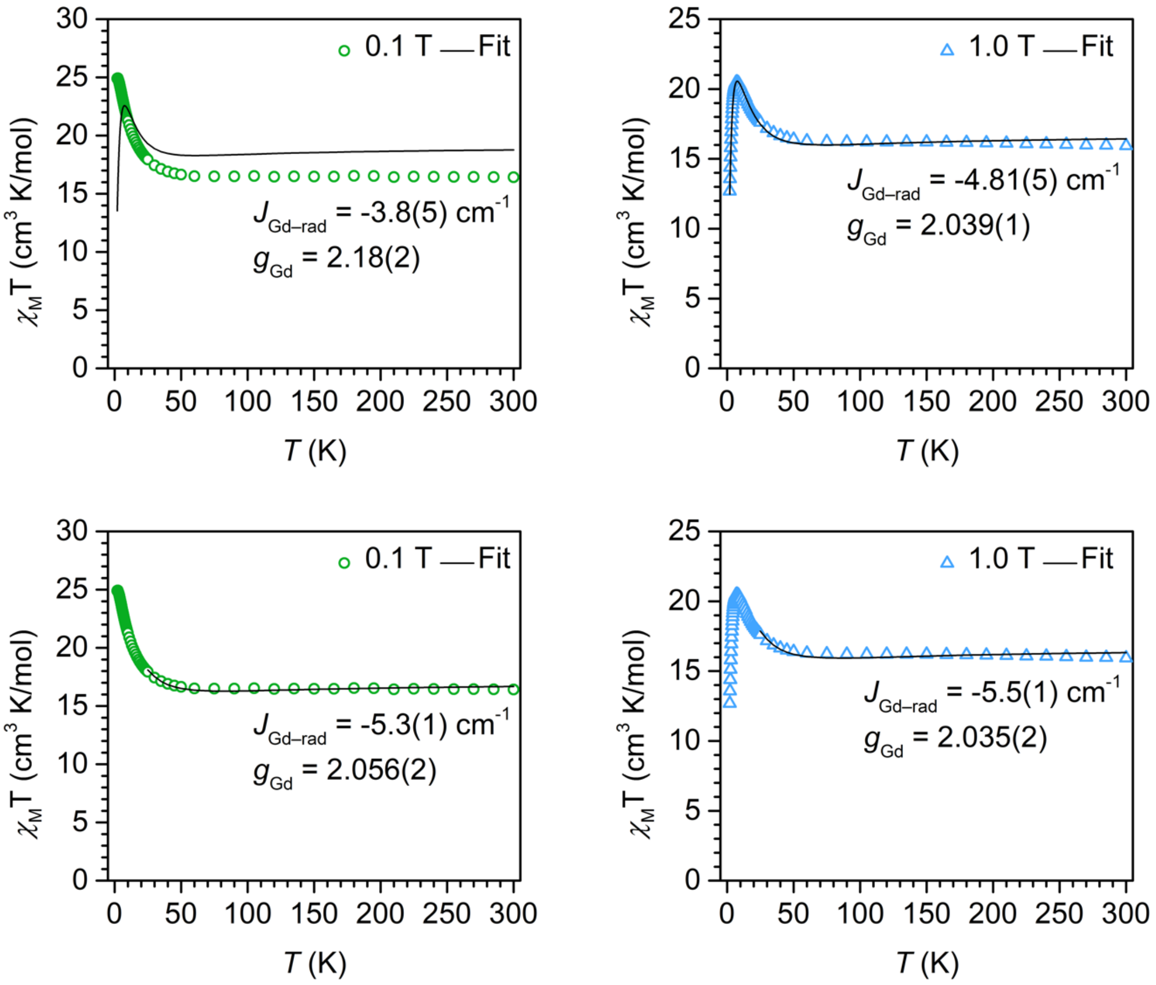

Figure S12. Variable-temperature dc magnetic susceptibility data for a restrained polycrystalline sample of **3-Gd** collected under 0.1 T (left), and 1.0 T (right) applied dc fields. The black lines represent fits to the data of **3-Gd** over the entire temperature range from 2 to 300 K (top) and from 25 to 300 K (bottom).

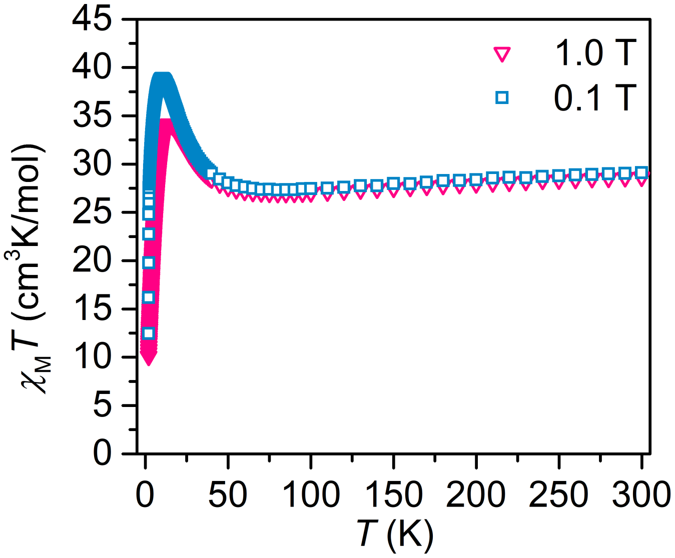

Figure S13. Variable-temperature dc magnetic susceptibility data for a restrained polycrystalline sample of **1-Dy** collected under 0.1 T (blue squares), and 1.0 T (pink triangles) applied dc fields.

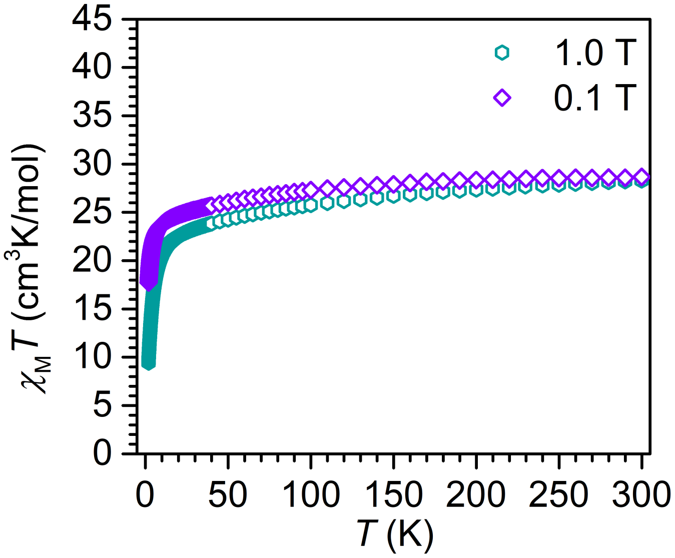

Figure S14. Variable-temperature dc magnetic susceptibility data for a restrained polycrystalline sample of **2-Dy** collected under 0.1 T (violet diamonds), and 1.0 T (teal hexagons) applied dc fields.

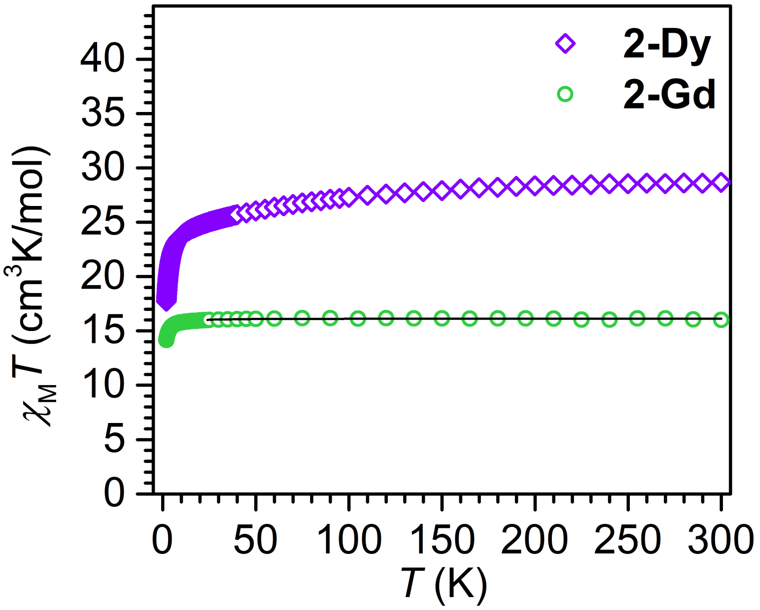

Figure S15. Variable-temperature dc magnetic susceptibility data for restrained polycrystalline samples of **2-Dy** (violet diamonds), and **2-Gd** (green circles), collected under a 0.1 T applied dc field. Black lines represent fits of the Gd data to a Heisenberg Hamiltonian, yielding $J_{Gd\text{-}Rad}$ = −0.005(2) cm^{-1} and g = 2.0235(7) (**2-Gd**).

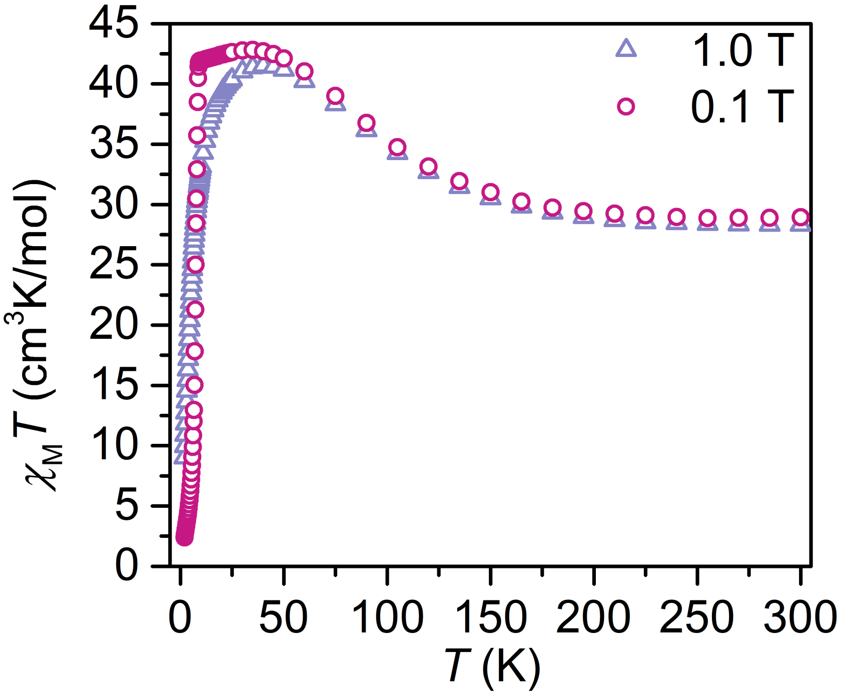

Figure S16. Variable-temperature dc magnetic susceptibility data for a restrained polycrystalline sample of **3-Dy** collected under 0.1 T (purple circles), and 1.0 T (lavender triangles) applied dc fields.

Table S6. Summary of fitting parameters J (cm^{-1}) and g for fits of the $\chi_M T$ vs. T data of **1-Gd – 3-Gd** at 0.1 T and 1.0 T.

***T* (K)**		**[(Cp*$_2$Gd)$_2$(μ-flv•)][Al(OC{CF$_3$}$_3$)$_4$] (1-Gd)**		**[(Cp*$_2$Gd)$_2$(μ-flv)] (2-Gd)**		**[K(crypt-222)][(Cp*$_2$Gd)$_2$(μ-flv•)] (3-Gd)**	
		0.1 T	1.0 T	0.1 T	1.0 T	0.1 T	1.0 T
25-300	J	–10.5(2)	–10.6(2)	–0.005(2)	–0.002(2)	–5.3(1)	–5.5(1)
	g	2.068(2)	2.050(3)	2.00235(7)	2.0058(6)	2.056(1)	2.035(2)
2-300	J	–8.1(4)	–28.1(26)	0.026(3)	–0.0135(2)	–3.8(5)	–4.81(5)
	g	2.075(6)	1.920(6)	2.005(4)	2.0085(3)	2.18(2)	2.039(1)

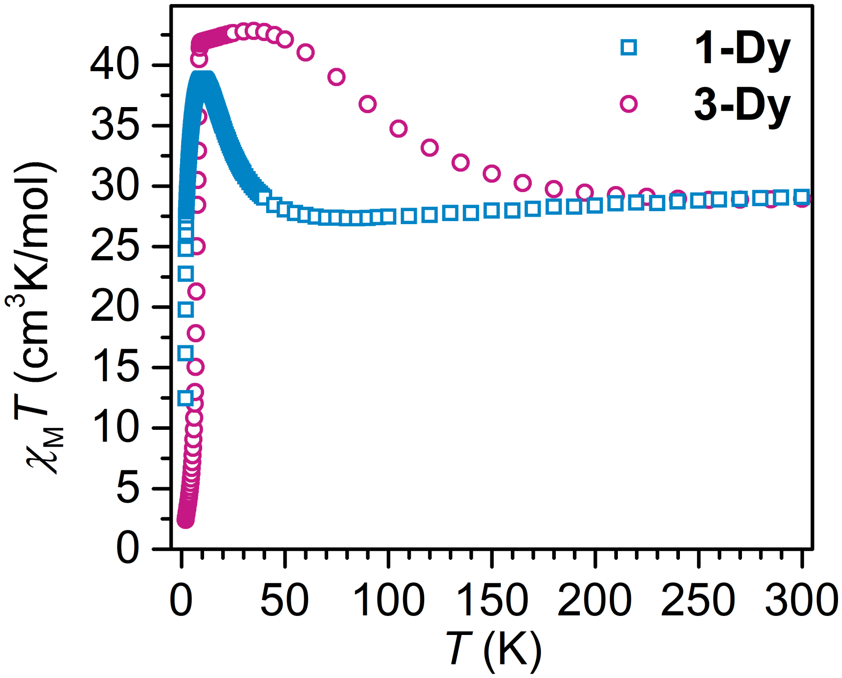

Figure S17. Variable-temperature dc magnetic susceptibility data for restrained polycrystalline samples of **1-Dy** (blue squares), and **3-Dy** (purple circles), collected under a 0.1 T applied dc field.

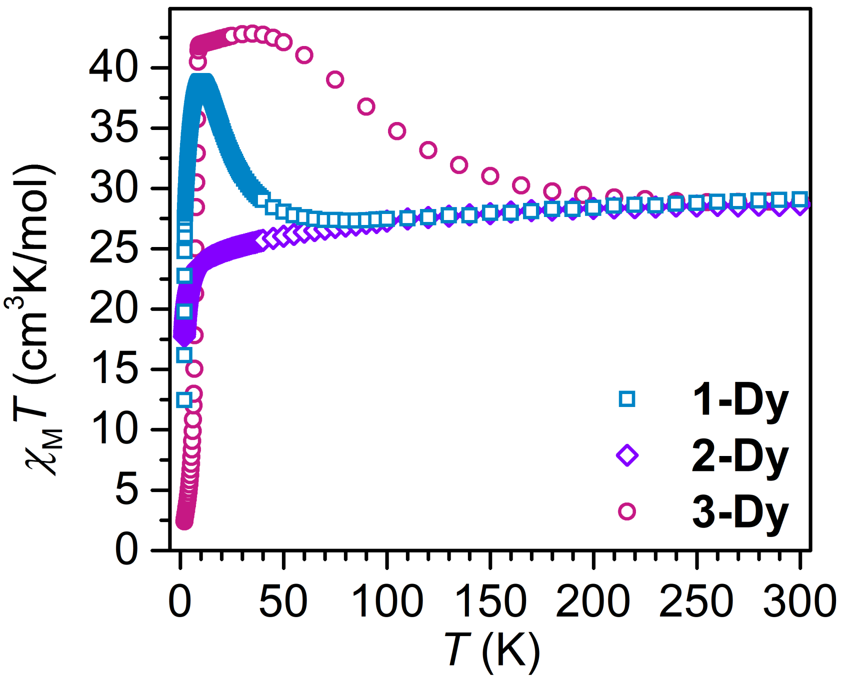

Figure S18. Variable-temperature dc magnetic susceptibility data for restrained polycrystalline samples of **1-Dy** (blue squares), **2-Dy** (violet diamonds), and **3-Dy** (purple circles), collected under a 0.1 T applied dc field.

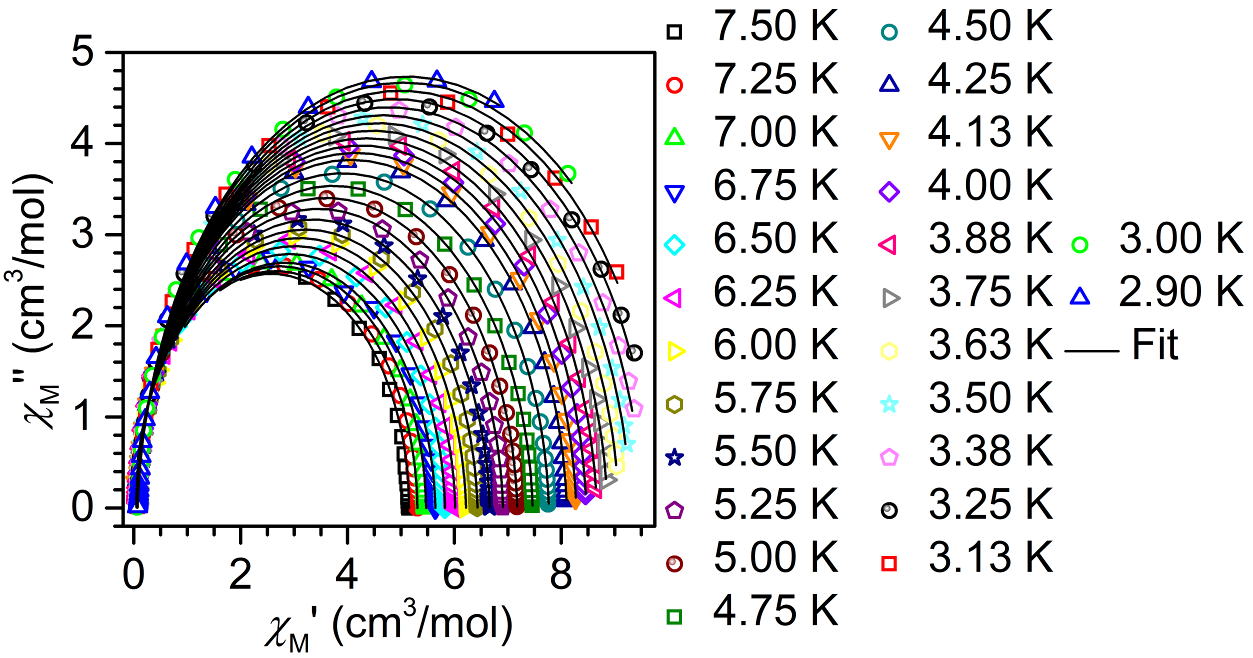

Figure S19. Cole-Cole (Argand) plots for ac susceptibility collected from 2.9 to 7.5 K under zero applied dc field for **1-Dy**. Symbols represent the experimental data points and the points representing the fits are connected by black solid lines.

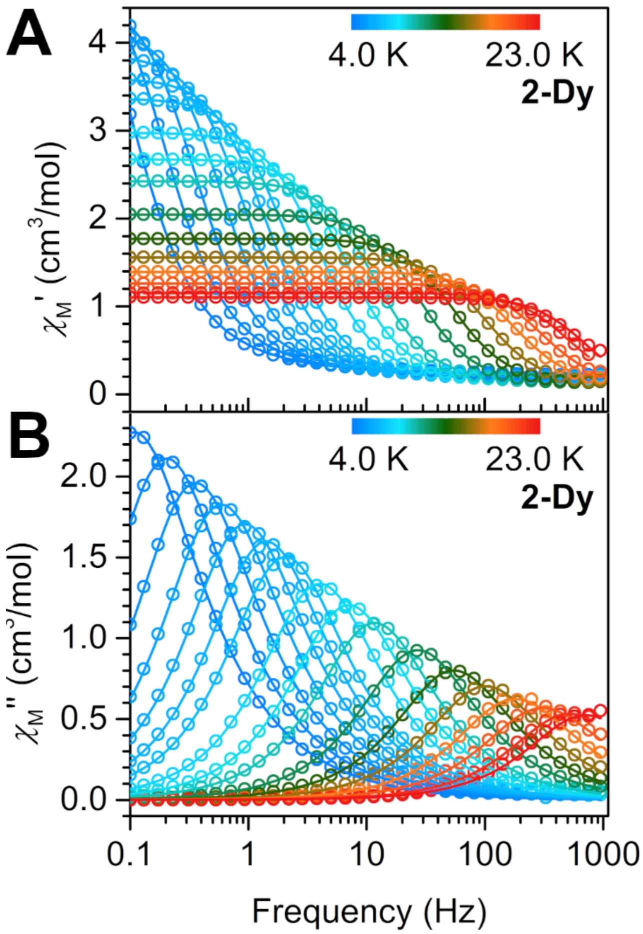

Figure S20. Variable-temperature, variable-frequency in-phase (χ_M', top) and out-of-phase (χ_M'', bottom) ac magnetic susceptibility data collected under zero applied dc field for **2-Dy**, from 4.0 to 23.0 K. Solid lines indicate the fits to the Cole-Davidson model.

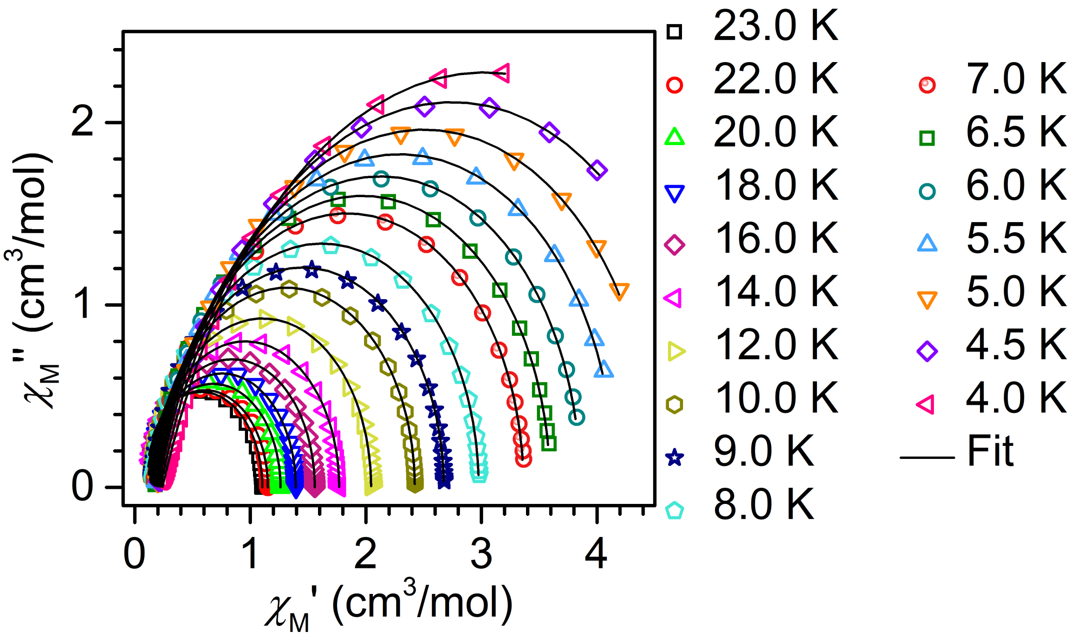

Figure S21. Cole-Cole (Argand) plots for ac susceptibility collected from 4.0 to 23.0 K under zero applied dc field for **2-Dy**. Symbols represent the experimental data points and the points representing the fits are connected by black solid lines.

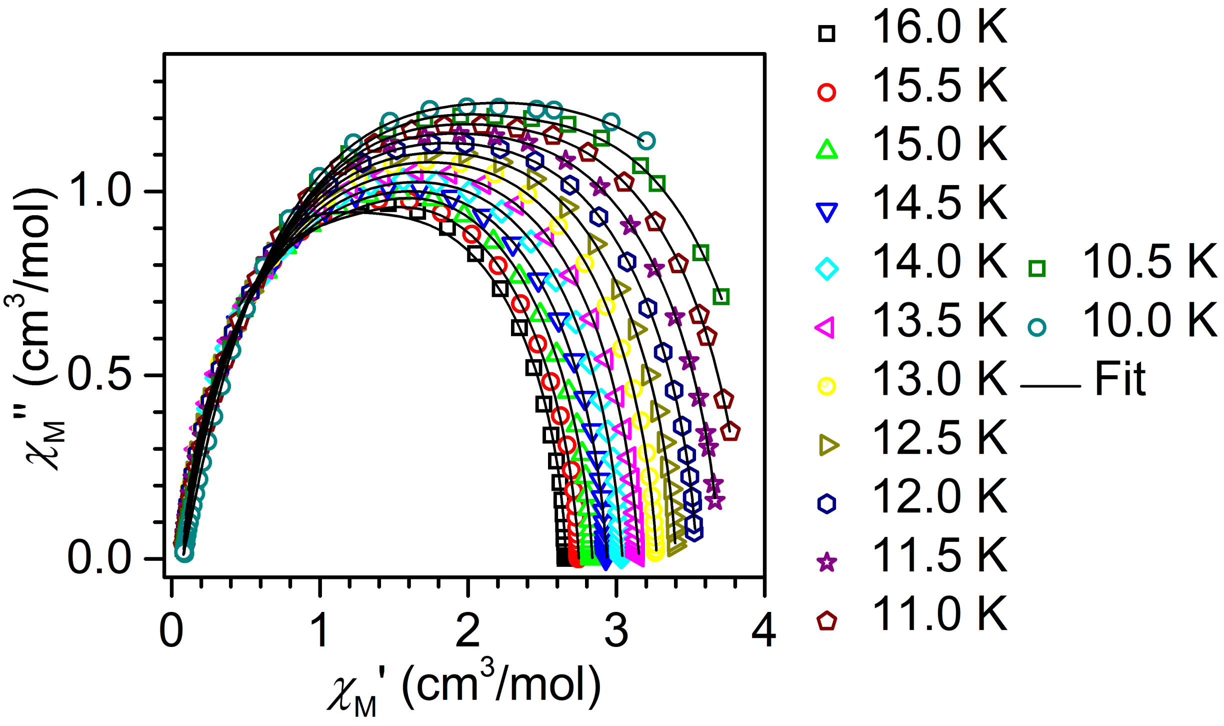

Figure S22. Cole-Cole (Argand) plots for ac susceptibility collected from 10.0 to 16.0 K under zero applied dc field for **3-Dy**. Symbols represent the experimental data points and the points representing the fits are connected by black solid lines.

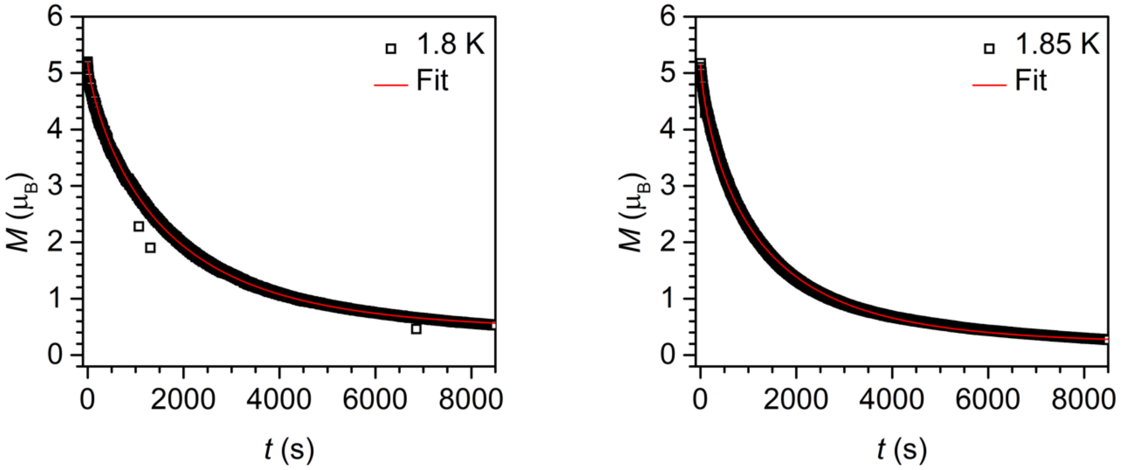

Figure S23. Plot of magnetization vs. time used to derive relaxation times for **1-Dy** at 1.8 K (left), and 1.85 K (right). The data (black squares) were fit (red line) to a function of the form $M(t) = M_{eq} + \left(M_0 - M_{eq}\right) \exp\left(-\left(\frac{t}{\tau *}\right)^{\beta}\right)$ where β is a stretch factor. Decays of the magnetization vs. time for **1-Dy** were obtained by applying a magnetic field of 7 T to the sample at each temperature for 5 min, and then quick removal of the magnetic field.

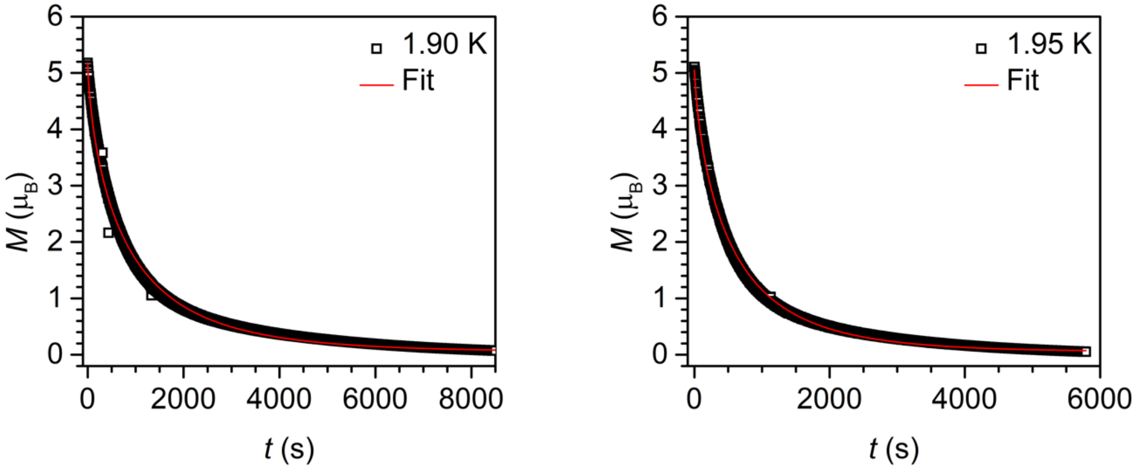

Figure S24. Plot of magnetization vs. time used to derive relaxation times for **1-Dy** at 1.90 K (left), and 1.95 K (right). The data (black squares) were fit (red line) to a function of the form $M(t) = M_{eq} + \left(M_0 - M_{eq}\right) \exp\left(-\left(\frac{t}{\tau *}\right)^{\beta}\right)$ where β is a stretch factor. Decays of the magnetization vs. time for **1-Dy** were obtained by applying a magnetic field of 7 T to the sample at each temperature for 5 min, and then quick removal of the magnetic field.

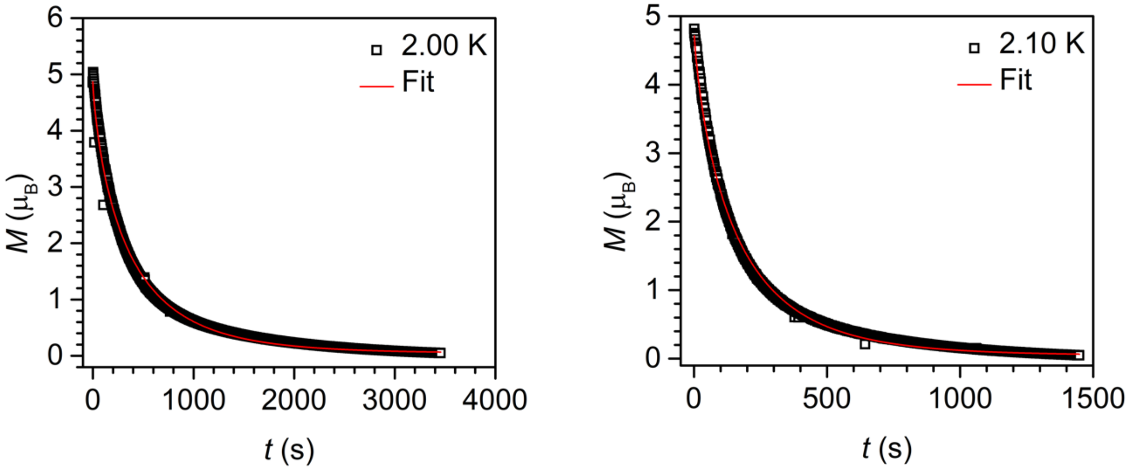

Figure S25. Plot of magnetization vs. time used to derive relaxation times for **1-Dy** at 2.00 K (left), and 2.10 K (right). The data (black squares) were fit (red line) to a function of the form $M(t) = M_{eq} + (M_0 - M_{eq})\exp\left(-\left(\frac{t}{\tau*}\right)^{\beta}\right)$ where β is a stretch factor. Decays of the magnetization vs. time for **1-Dy** were obtained by applying a magnetic field of 7 T to the sample at each temperature for 5 min, and then quick removal of the magnetic field.

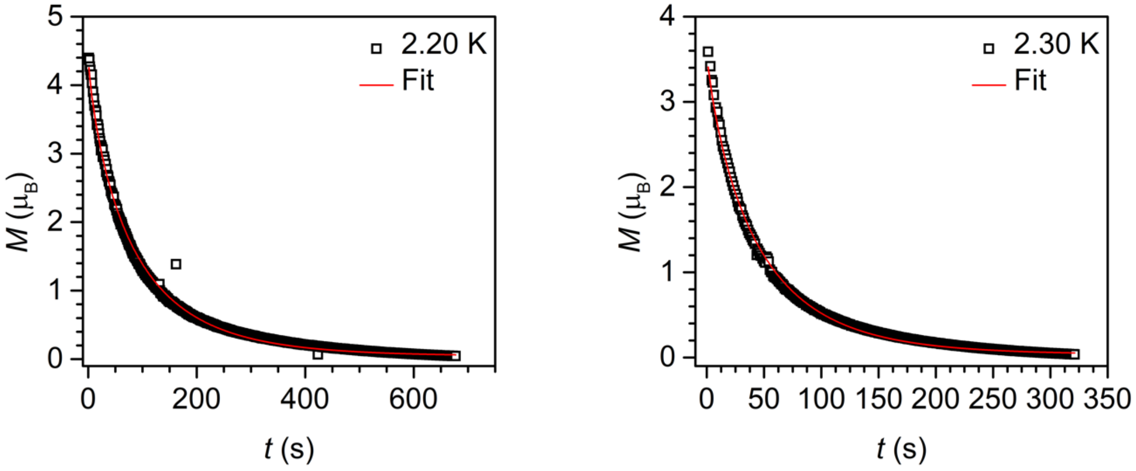

Figure S26. Plot of magnetization vs. time used to derive relaxation times for **1-Dy** at 2.20 K (left), and 2.30 K (right). The data (black squares) were fit (red line) to a function of the form $M(t) = M_{eq} + (M_0 - M_{eq})\exp\left(-\left(\frac{t}{\tau*}\right)^{\beta}\right)$ where β is a stretch factor. Decays of the magnetization vs. time for **1-Dy** were obtained by applying a magnetic field of 7 T to the sample at each temperature for 5 min, and then quick removal of the magnetic field.

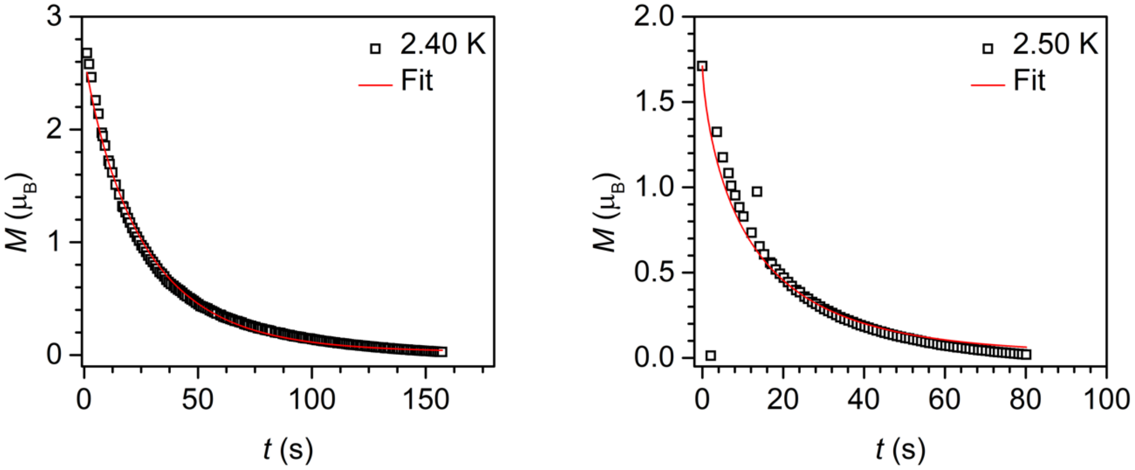

Figure S27. Plot of magnetization vs. time used to derive relaxation times for **1-Dy** at 2.40 K (left), and 2.50 K (right). The data (black squares) were fit (red line) to a function of the form $M(t) = M_{eq} + \left(M_0 - M_{eq}\right)\exp\left(-\left(\frac{t}{\tau*}\right)^{\beta}\right)$ where β is a stretch factor. Decays of the magnetization vs. time for **1-Dy** were obtained by applying a magnetic field of 7 T to the sample at each temperature for 5 min, and then quick removal of the magnetic field.

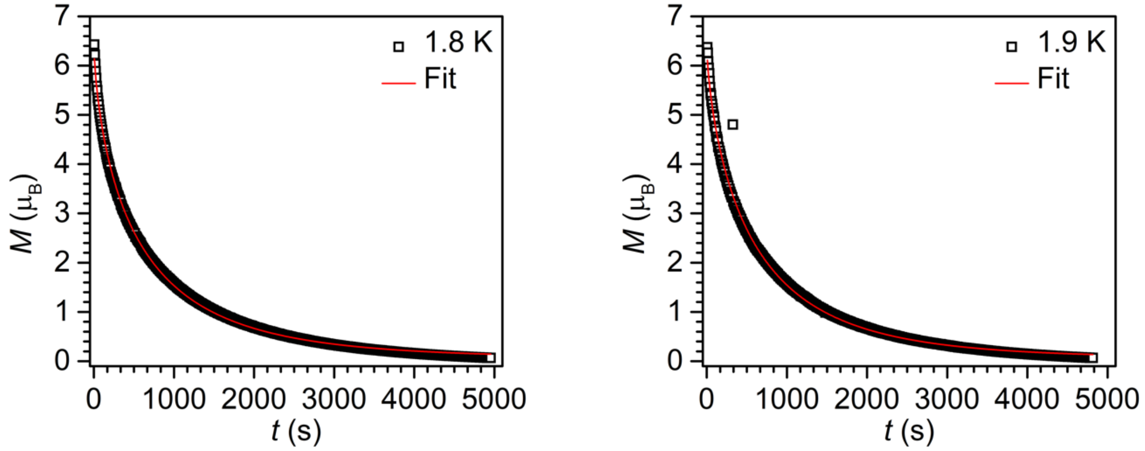

Figure S28. Plot of magnetization vs. time used to derive relaxation times for **3-Dy** at 1.8 K (left), and 1.9 K (right). The data (black squares) were fit (red line) to a function of the form $M(t) = M_{eq} + \left(M_0 - M_{eq}\right)\exp\left(-\left(\frac{t}{\tau*}\right)^{\beta}\right)$ where β is a stretch factor. Decays of the magnetization vs. time for **3-Dy** were obtained by applying a magnetic field of 7 T to the sample at each temperature for 5 min, and then quick removal of the magnetic field.

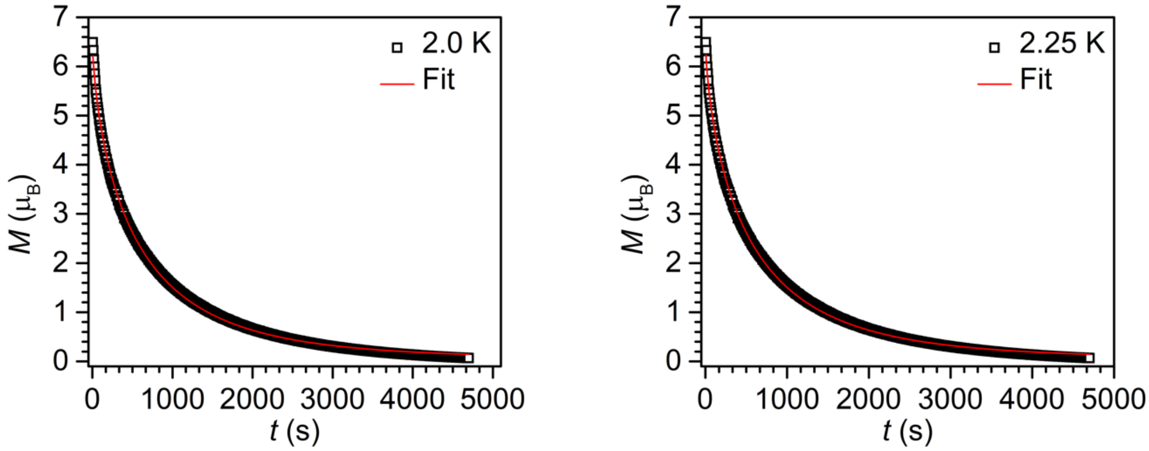

Figure S29. Plot of magnetization vs. time used to derive relaxation times for **3-Dy** at 2.0 K (left), and 2.25 K (right). The data (black squares) were fit (red line) to a function of the form $M(t) = M_{eq} + (M_0 - M_{eq}) \exp\left(-\left(\frac{t}{\tau *}\right)^{\beta}\right)$ where β is a stretch factor. Decays of the magnetization vs. time for **3-Dy** were obtained by applying a magnetic field of 7 T to the sample at each temperature for 5 min, and then quick removal of the magnetic field.

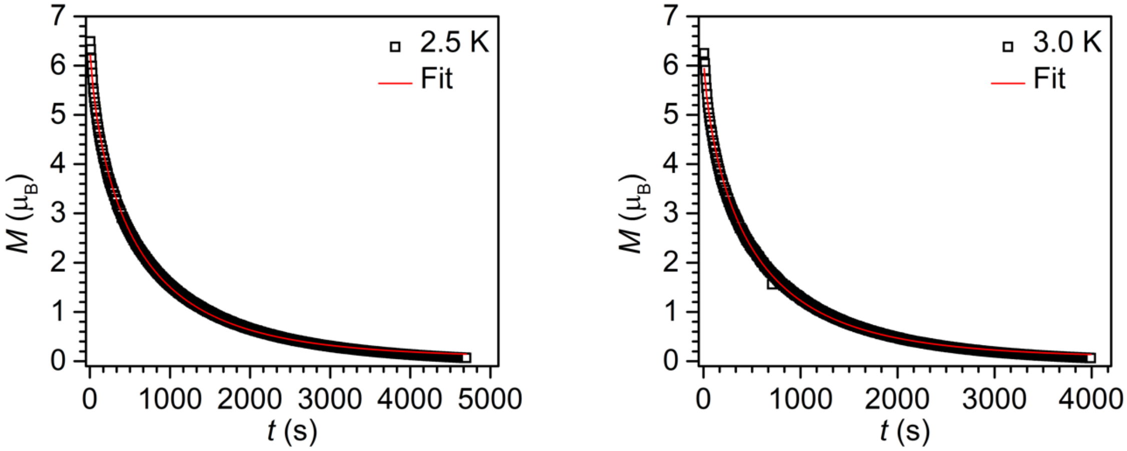

Figure S30. Plot of magnetization vs. time used to derive relaxation times for **3-Dy** at 2.5 K (left), and 3.0 K (right). The data (black squares) were fit (red line) to a function of the form $M(t) = M_{eq} + (M_0 - M_{eq}) \exp\left(-\left(\frac{t}{\tau *}\right)^{\beta}\right)$ where β is a stretch factor. Decays of the magnetization vs. time for **3-Dy** were obtained by applying a magnetic field of 7 T to the sample at each temperature for 5 min, and then quick removal of the magnetic field.

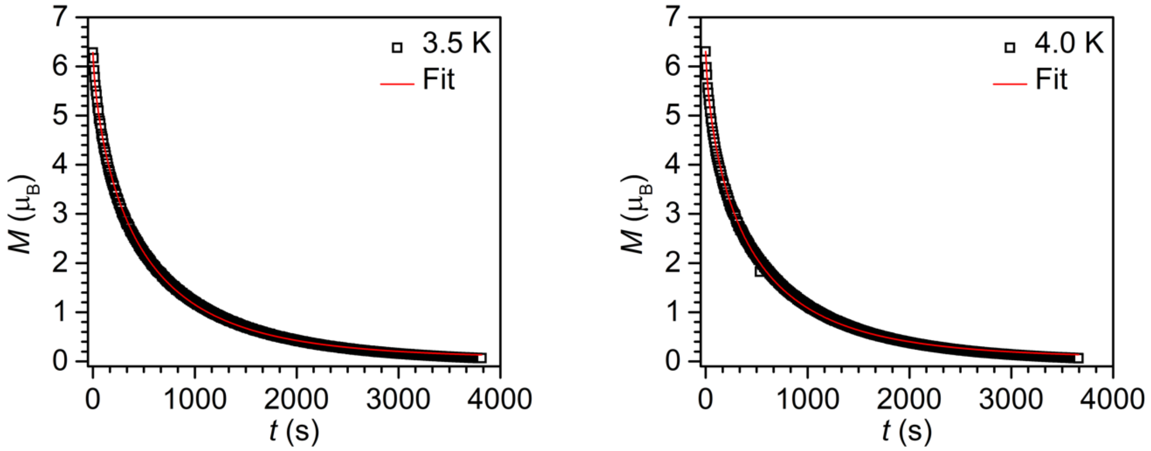

Figure S31. Plot of magnetization vs. time used to derive relaxation times for **3-Dy** at 3.5 K (left), and 4.0 K (right). The data (black squares) were fit (red line) to a function of the form $M(t) = M_{eq} + (M_0 - M_{eq})\exp\left(-\left(\frac{t}{\tau*}\right)^{\beta}\right)$ where β is a stretch factor. Decays of the magnetization vs. time for **3-Dy** were obtained by applying a magnetic field of 7 T to the sample at each temperature for 5 min, and then quick removal of the magnetic field.

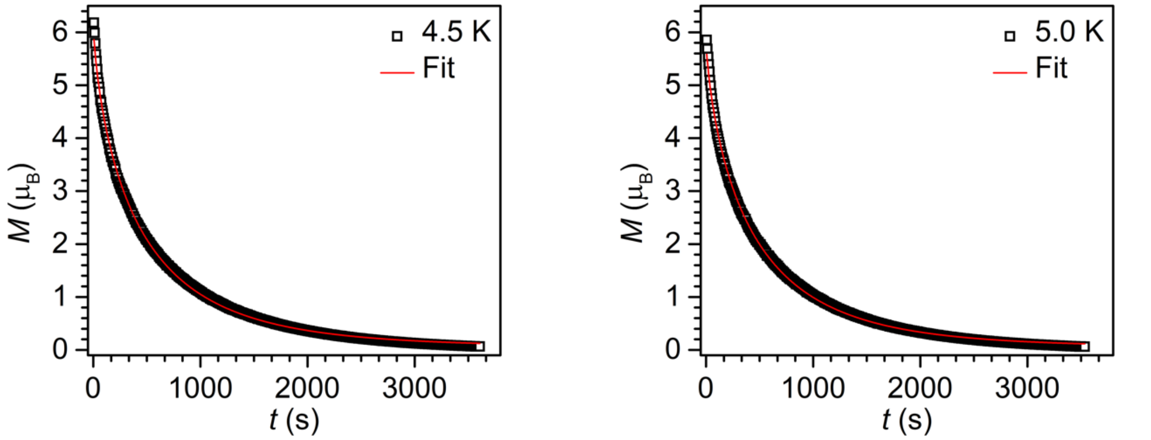

Figure S32. Plot of magnetization vs. time used to derive relaxation times for **3-Dy** at 4.5 K (left), and 5.0 K (right). The data (black squares) were fit (red line) to a function of the form $M(t) = M_{eq} + (M_0 - M_{eq})\exp\left(-\left(\frac{t}{\tau*}\right)^{\beta}\right)$ where β is a stretch factor. Decays of the magnetization vs. time for **3-Dy** were obtained by applying a magnetic field of 7 T to the sample at each temperature for 5 min, and then quick removal of the magnetic field.

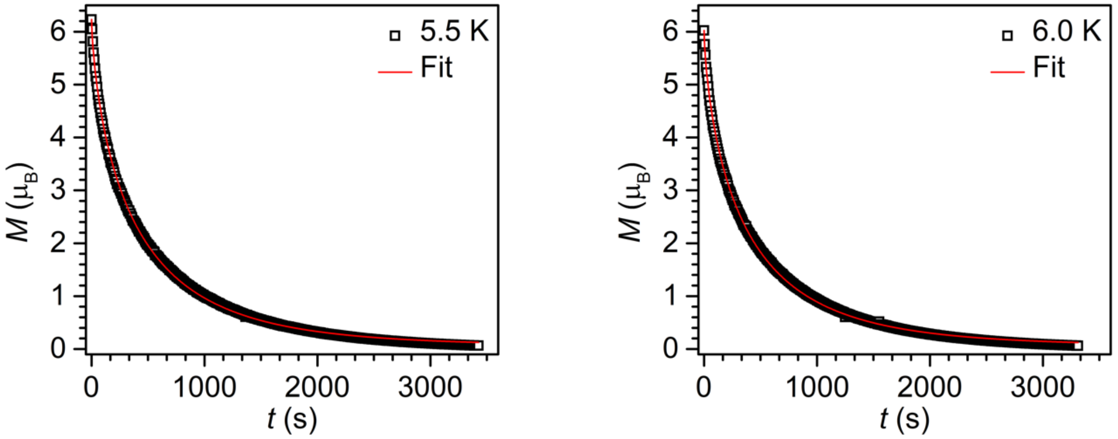

Figure S33. Plot of magnetization vs. time used to derive relaxation times for **3-Dy** at 5.5 K (left), and 6.0 K (right). The data (black squares) were fit (red line) to a function of the form $M(t) = M_{eq} + \left(M_0 - M_{eq}\right) \exp\left(-\left(\frac{t}{\tau*}\right)^{\beta}\right)$ where β is a stretch factor. Decays of the magnetization vs. time for **3-Dy** were obtained by applying a magnetic field of 7 T to the sample at each temperature for 5 min, and then quick removal of the magnetic field.

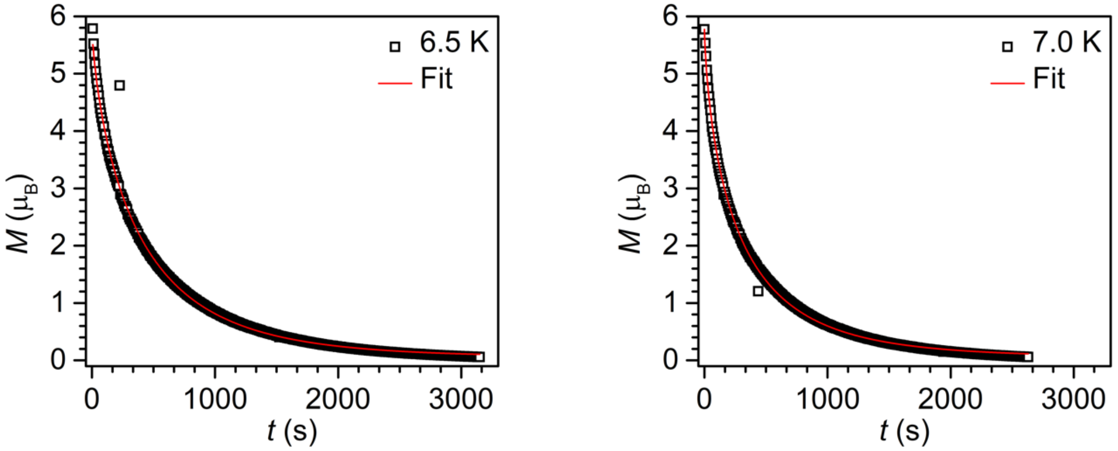

Figure S34. Plot of magnetization vs. time used to derive relaxation times for **3-Dy** at 6.5 K (left), and 7.0 K (right). The data (black squares) were fit (red line) to a function of the form $M(t) = M_{eq} + \left(M_0 - M_{eq}\right) \exp\left(-\left(\frac{t}{\tau*}\right)^{\beta}\right)$ where β is a stretch factor. Decays of the magnetization vs. time for **3-Dy** were obtained by applying a magnetic field of 7 T to the sample at each temperature for 5 min, and then quick removal of the magnetic field.

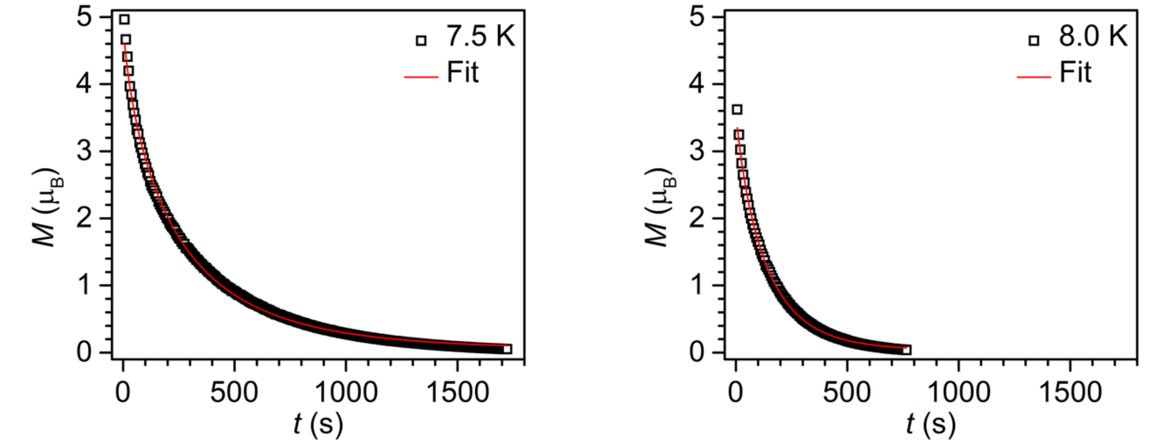

Figure S35. Plot of magnetization vs. time used to derive relaxation times for **3-Dy** at 7.5 K (left), and 8.0 K (right). The data (black squares) were fit (red line) to a function of the form $M(t) = M_{eq} + (M_0 - M_{eq})\exp\left(-\left(\frac{t}{\tau*}\right)^{\beta}\right)$ where β is a stretch factor. Decays of the magnetization vs. time for **3-Dy** were obtained by applying a magnetic field of 7 T to the sample at each temperature for 5 min, and then quick removal of the magnetic field.

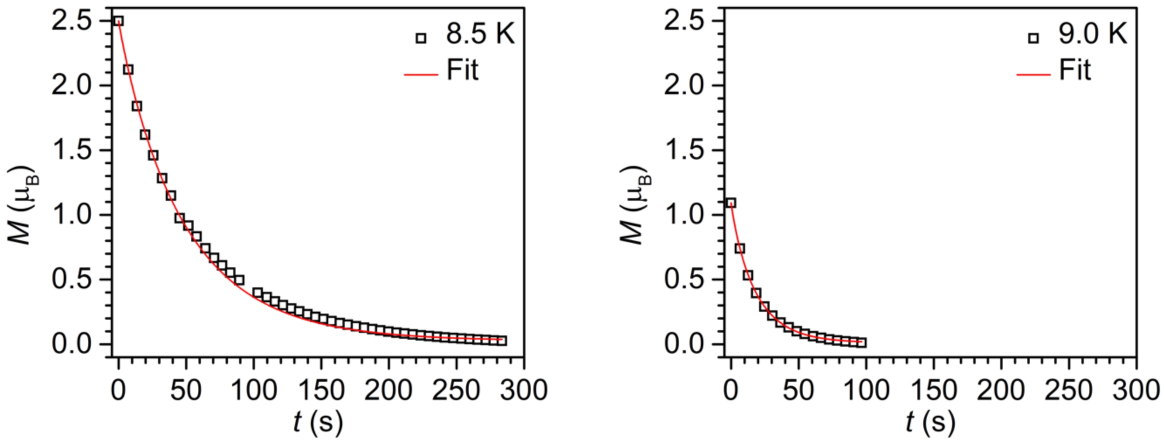

Figure S36. Plot of magnetization vs. time used to derive relaxation times for **3-Dy** at 8.5 K (left), and 9.0 K (right). The data (black squares) were fit (red line) to a function of the form $M(t) = M_{eq} + (M_0 - M_{eq})\exp\left(-\left(\frac{t}{\tau*}\right)^{\beta}\right)$ where β is a stretch factor. Decays of the magnetization vs. time for **3-Dy** were obtained by applying a magnetic field of 7 T to the sample at each temperature for 5 min, and then quick removal of the magnetic field.

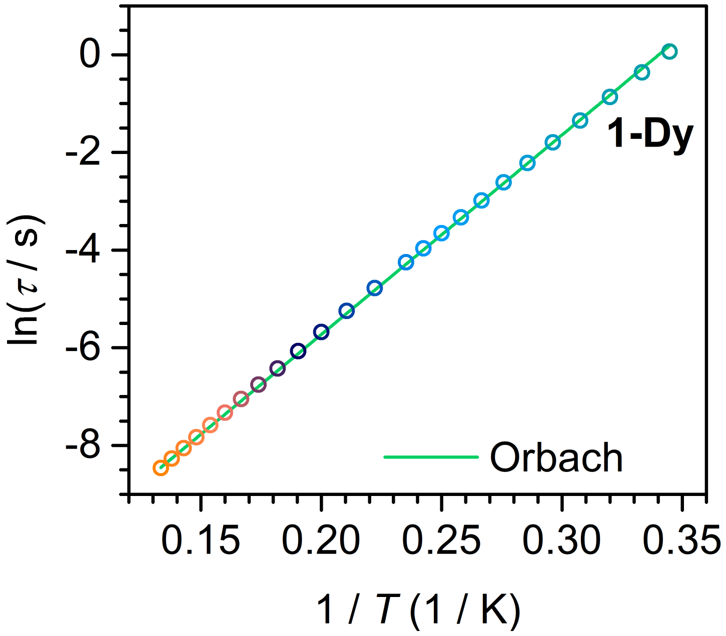

Figure S37. Plot of natural log of the relaxation time versus the inverse temperature for **1-Dy** (temperature range 2.9 to 7.5 K). Turquoise to orange circles represent data extracted from ac magnetic susceptibility measurements under zero applied dc field. The green line represents a fit to a single Orbach relaxation process yielding U_{eff} = 28.36(7) cm^{-1} and τ_0 = 9.2(2) x 10^{-7} s. Parameters used to calculate the contributions are given in Table S8.

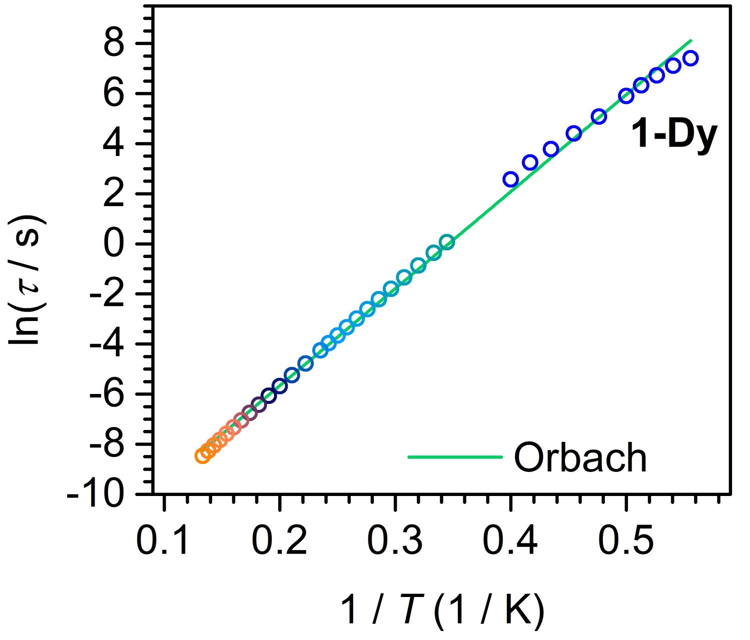

Figure S38. Plot of natural log of the relaxation time versus the inverse temperature for **1-Dy** (temperature range 1.8 to 7.5 K). Turquoise to orange circles represent data extracted from ac magnetic susceptibility measurements under zero applied dc field (temperature range 2.9 to 7.5 K), and dark blue circles represent data extracted from dc relaxation experiments (temperature range 1.8 to 2.5 K). The green line represents a fit to a single Orbach relaxation process yielding

U_{eff} = 26.9(2) cm^{-1} and τ_0 = 1.5(2) x 10^{-6} s. Parameters used to calculate the contributions are given in Table S8.

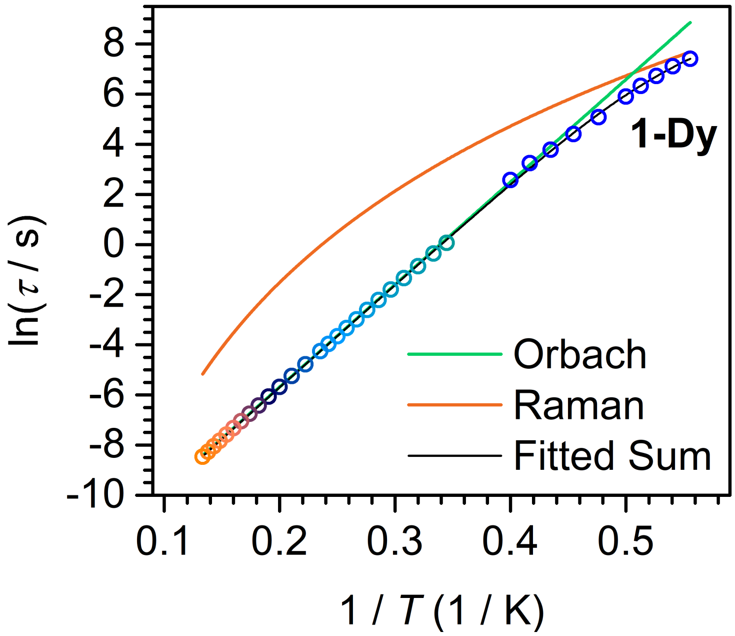

Figure S39. Individual contributions of the multiple magnetic relaxation pathways to the Arrhenius plot of **1-Dy**, from 1.8 to 7.5 K. Turquoise to orange circles represent data extracted from ac magnetic susceptibility measurements under zero applied dc field (temperature range 2.9 to 7.5 K), and dark blue circles represent data extracted from dc relaxation experiments (temperature range 1.8 to 2.5 K). Individual parameters used to calculate the contributions are given in Table S8. The black line represents a fit to an Orbach relaxation process and a Raman pathway yielding U_{eff} = 28.4(1) cm^{-1} and τ_0 = 9.6(4) x 10^{-7} s.

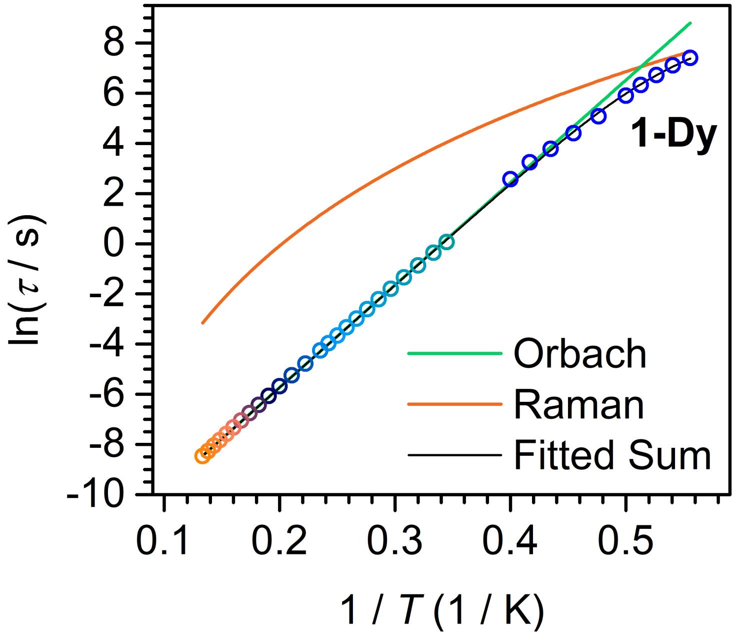

Figure S40. Individual contributions of the multiple magnetic relaxation pathways to the Arrhenius plot of **1-Dy**, from 1.8 to 7.5 K. Individual parameters used to calculate the contributions are given in Table S8. Turquoise to orange circles represent data extracted from ac magnetic susceptibility measurements under zero applied dc field (temperature range 2.9 to 7.5 K), and dark blue circles represent data extracted from dc relaxation experiments (temperature range 1.8 to 2.5 K). The black line represents a fit to an Orbach relaxation process and a Raman pathway yielding U_{eff} = 28.36 cm^{-1} and τ_0 = 9.2 x 10^{-7} s. The Orbach term was fixed to the values obtained from fitting the ac data.

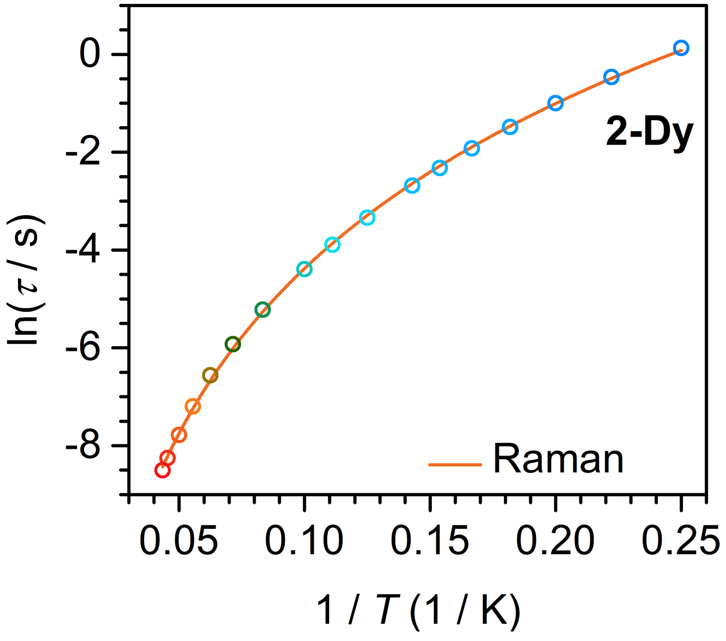

Figure S41. Plot of natural log of the relaxation time versus the inverse temperature for **2-Dy** (temperature range 4.0 to 23.0 K). The orange line represents a fit to a Raman process.

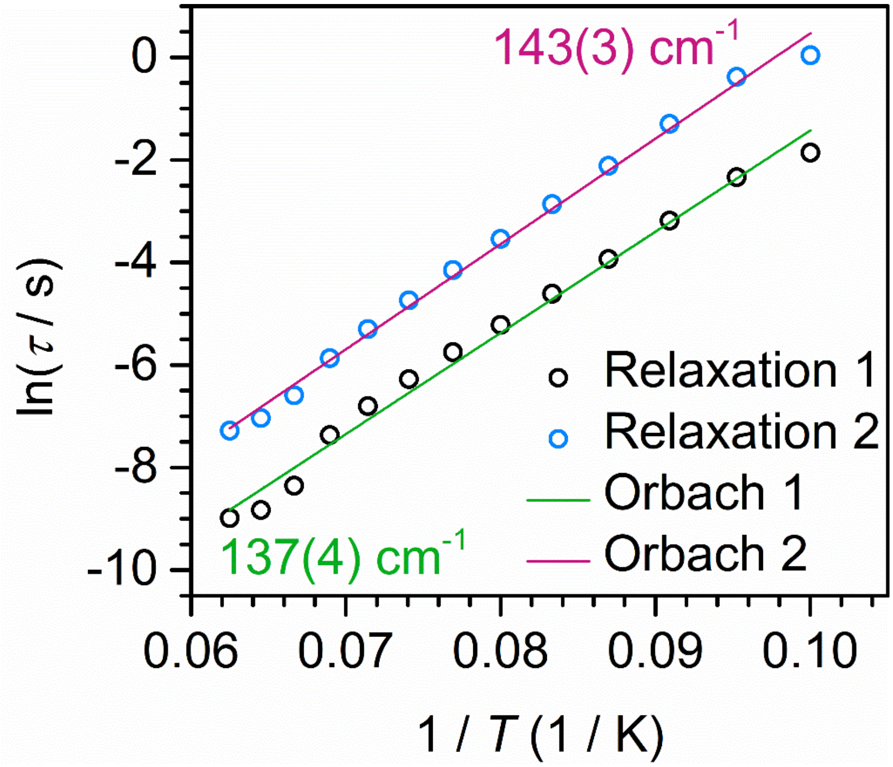

Figure S42. Plot of natural log of the relaxation time versus the inverse temperature for **3-Dy** (temperature range 10 to 16 K, blue and black circles). The green (process 1) and pink (process 2) lines represent fits to an Orbach relaxation mechanism yielding $U_{eff(1)}$ = 137(4) cm^{-1} and $\tau_{0(1)}$ = 6.4(33) x 10^{-10} s, and $U_{eff(2)}$ = 143(3) cm^{-1} and $\tau_{0(2)}$ = 1.9(7) x 10^{-9} s, respectively.

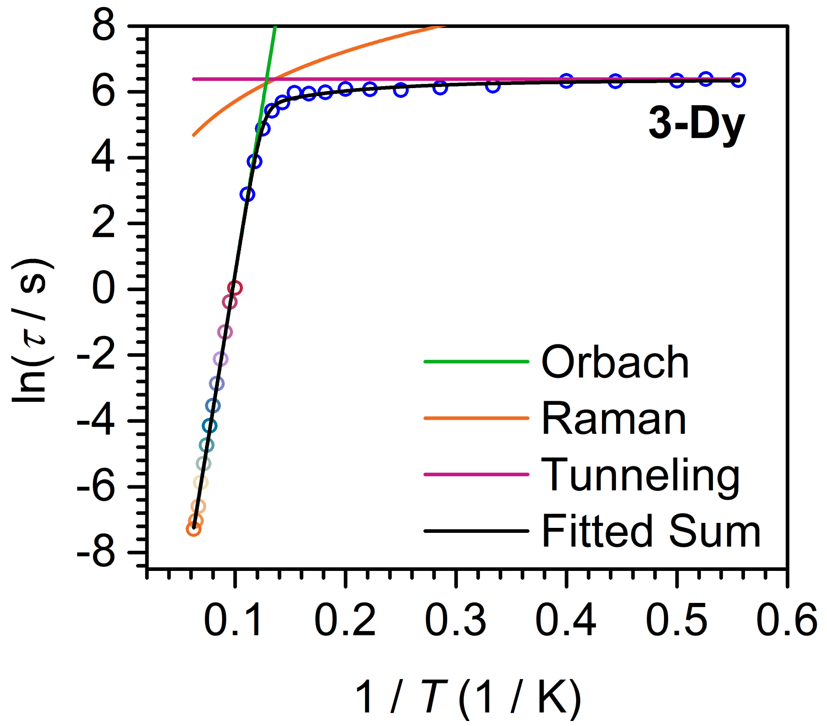

Figure S43. Individual contributions of the multiple magnetic relaxation pathways to the Arrhenius plot of **3-Dy**, from 1.8 to 16 K. Red to orange circles represent data extracted from ac magnetic susceptibility measurements under zero applied dc field (process 2), and blue circles represent data extracted from dc relaxation experiments. Individual parameters used to calculate the contributions are given in Table S8. The black line represents a fit to an Orbach relaxation process, a Raman, and a Quantum Tunneling pathway yielding U_{eff} = 143(2) cm^{-1} and τ_0 = 1.8(3) x 10^{-9} s.

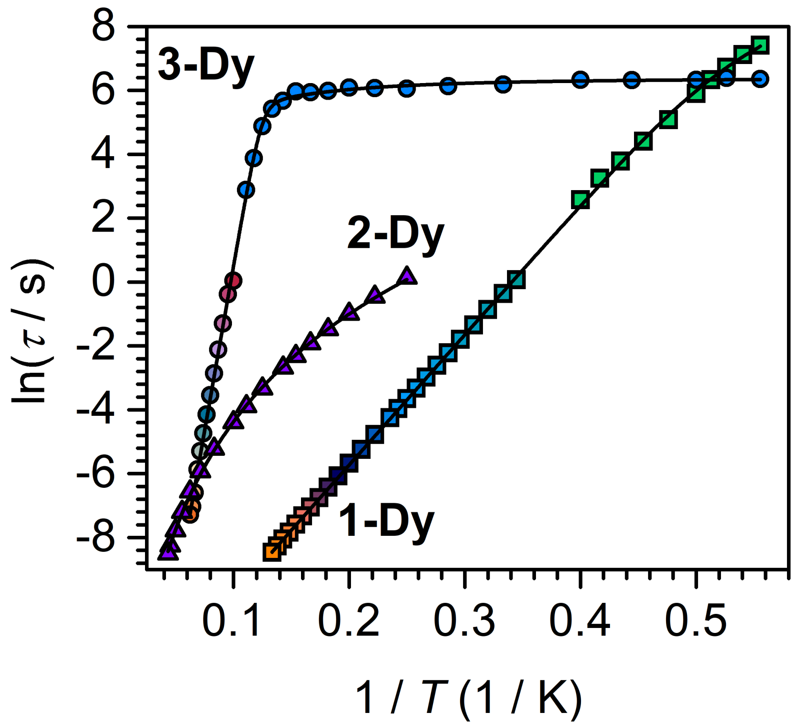

Figure S44. Plot of natural log of the relaxation time versus the inverse temperature for **1-Dy** (temperature range 1.8 to 7.5 K), for **2-Dy** (temperature range 4.0 to 23.0 K), and for **3-Dy** (temperature range 1.8 to 16 K). For **1-Dy**: Teal to orange squares represent data extracted from ac magnetic susceptibility measurements under zero applied dc field (2.9 to 7.5 K), and green squares represent data extracted from dc relaxation experiments (1.8 to 2.5 K). For **2-Dy**: Violet triangles depict data extracted from ac magnetic susceptibility measurements under zero applied dc field (uniform color for clarity). For **3-Dy**: Red to orange circles represent data extracted from ac magnetic susceptibility measurements under zero applied dc field (10 to 16 K), and blue circles represent data extracted from dc relaxation experiments (temperature range 1.8 to 9 K). For **1-Dy**, the black line describes a fit to a Raman and an Orbach relaxation process. For **2-Dy**, the black line describes a fit to a Raman relaxation process. For **3-Dy,** the black line represents a fit to a QTM, a Raman, and an Orbach relaxation mechanism.

Table S7. Relaxation times of **1-Dy**, **2-Dy**, and **3-Dy** at various temperatures. Data in blue and green were extracted from ac magnetic susceptibility measurements and dc relaxation experiments, respectively.

**1-Dy**		**2-Dy**		**3-Dy**	
***T* (K)**	**τ (s)**	***T* (K)**	**τ (s)**	***T* (K)**	**τ (s)**
1.80	1.65E+03	4.00	1.14E+00	1.80	5.74E+02
1.85	1.23E+03	4.50	6.30E-01	1.90	5.96E+02

1.90	8.31E+02	5.00	3.68E-01	2.00	5.63E+02
1.95	5.59E+02	5.50	2.27E-01	2.25	5.58E+02
2.00	3.66E+02	6.00	1.46E-01	2.50	5.63E+02
2.10	1.61E+02	6.50	9.78E-02	3.00	4.86E+02
2.20	8.16E+01	7.00	6.81E-02	3.50	4.59E+02
2.30	4.37E+01	8.00	3.54E-02	4.00	4.25E+02
2.40	2.58E+01	9.00	2.03E-02	4.50	4.35E+02
2.50	1.31E+01	10.00	1.24E-02	5.00	4.41E+02
2.90	1.07E+00	12.00	5.39E-03	5.50	3.97E+02
3.00	6.95E-01	14.00	2.67E-03	6.00	3.80E+02
3.13	4.20E-01	16.00	1.41E-03	6.50	3.91E+02
3.25	2.60E-01	18.00	7.48E-04	7.00	2.92E+02
3.38	1.66E-01	20.00	4.17E-04	7.50	2.26E+02
3.50	1.09E-01	22.00	2.61E-04	8.00	1.31E+02
3.63	7.32E-02	23.00	2.02E-04	8.50	4.83E+01
3.75	5.06E-02			9.00	1.79E+01
3.88	3.58E-02			10.00	1.04E+00
4.00	2.58E-02			10.50	6.82E-01
4.13	1.90E-02			11.00	2.73E-01
4.25	1.42E-02			11.50	1.20E-01
4.50	8.40E-03			12.00	5.70E-02
4.75	5.26E-03			12.50	2.90E-02
5.00	3.42E-03			13.00	1.58E-02
5.25	2.31E-03			13.50	8.76E-03
5.50	1.62E-03			14.00	5.00E-03
5.75	1.17E-03			14.50	2.83E-03
6.00	8.67E-04			15.00	1.37E-03
6.25	6.56E-04			15.50	8.79E-04
6.50	5.08E-04			16.00	6.87E-04
6.75	3.96E-04				
7.00	3.18E-04				
7.25	2.57E-04				
7.50	2.11E-04				

Table S8. Best-Fit Parameters for the Arrhenius plots of **1-Dy**, **2-Dy**, and **3-Dy**.

	U_{eff} (cm^{-1})	τ_0 (s)	C (s^{-1}K^{-n})	n	τ_{QTM} (s)
**1-Dy**[a]	28.36(7)	9.2(2) x 10^{-7}	-	-	-
**1-Dy**[b]	26.9(2)	1.5(2) x 10^{-6}	-	-	-
**1-Dy**[b]	28.4(1)	9.6(4) x 10^{-7}	2.3(12) x 10^{-6}	9.0(8)	-
**1-Dy**[bc]	28.36	9.2 x 10^{-7}	5.5(34) x 10^{-6}	7.6(9)	-
**2-Dy**	-	-	1.08(6) x 10^{-3}	4.87(2)	-
**3-Dy (A)**[a]	137(4)	6.4(33) x 10^{-10}	-	-	-
**3-Dy (B)**[a]	143(3)	1.9(7) x 10^{-9}	-	-	-
**3-Dy**[b]	143(2)	1.8(3) x 10^{-9}	2.2(81) x 10^{-5}	2.2(10)	594(74)

[a]Data extracted from ac susceptibility measurements. [b]Data extracted from ac and dc susceptibility measurements. [c]Orbach parameters fixed to ac fits.

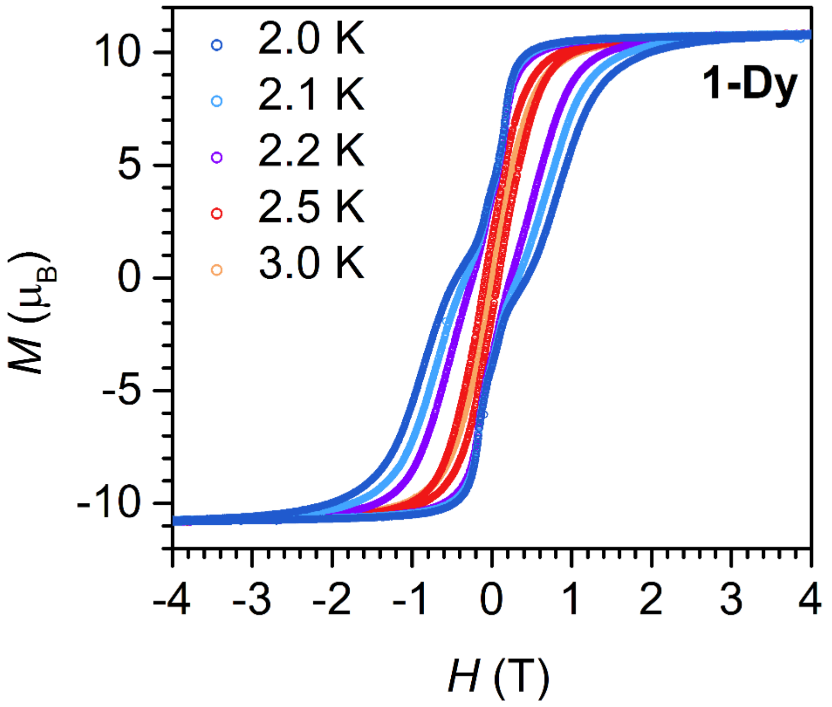

Figure S45. Plot of magnetization (M) vs magnetic field (H) at an average sweep rate of 0.01 T/s for **1-Dy** between 2 and 3 K.

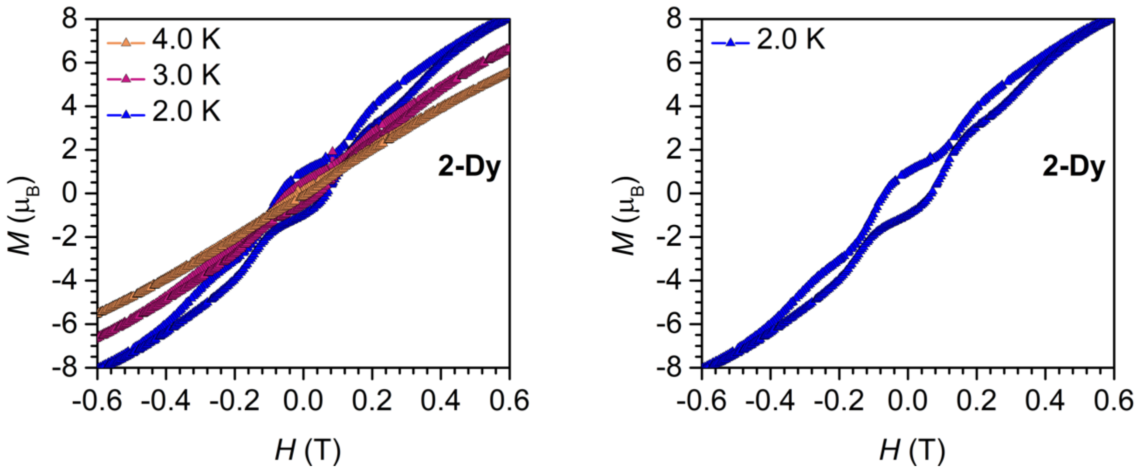

Figure S46. Plot of magnetization (*M*) vs. dc magnetic field (*H*) at an average sweep rate of 0.01 T/s for **2-Dy** from 2.0 to 4.0 K (left), and at 2.0 K (right).

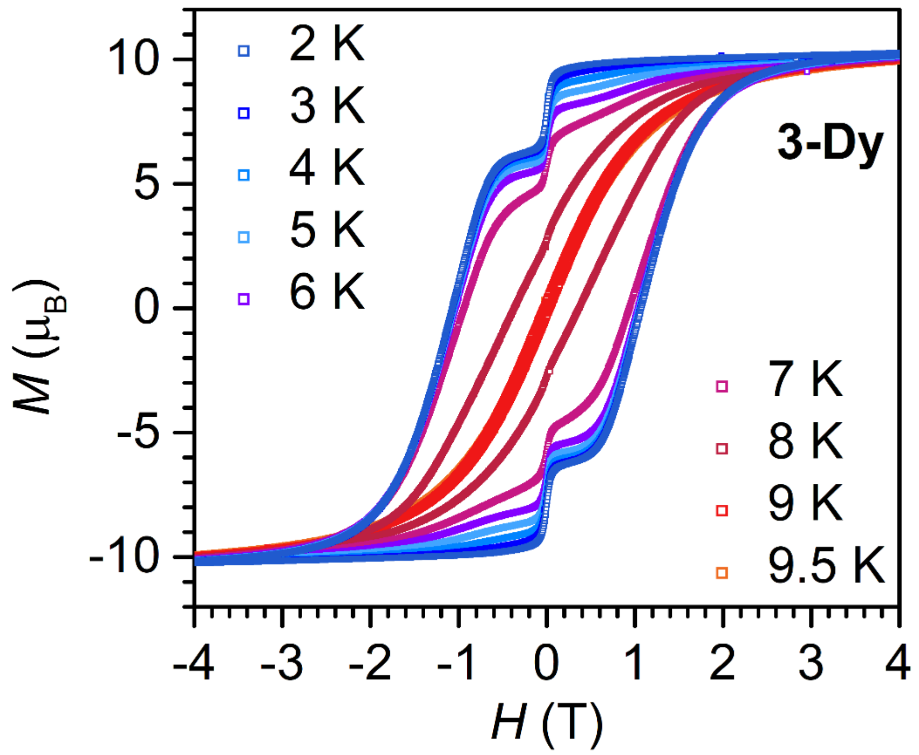

Figure S47. Plot of magnetization (*M*) vs magnetic field (*H*) at an average sweep rate of 0.01 T/s for **3-Dy** between 2 and 9.5 K.

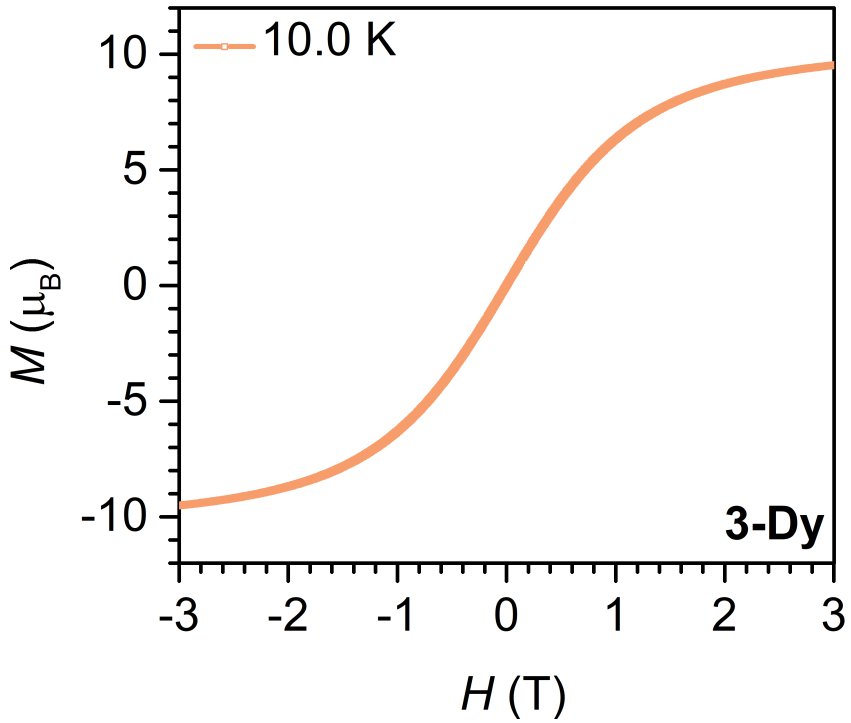

Figure S48. Plot of magnetization (*M*) vs. dc magnetic field (*H*) at an average sweep rate of 0.01 T/s for **3-Dy** at 10 K.

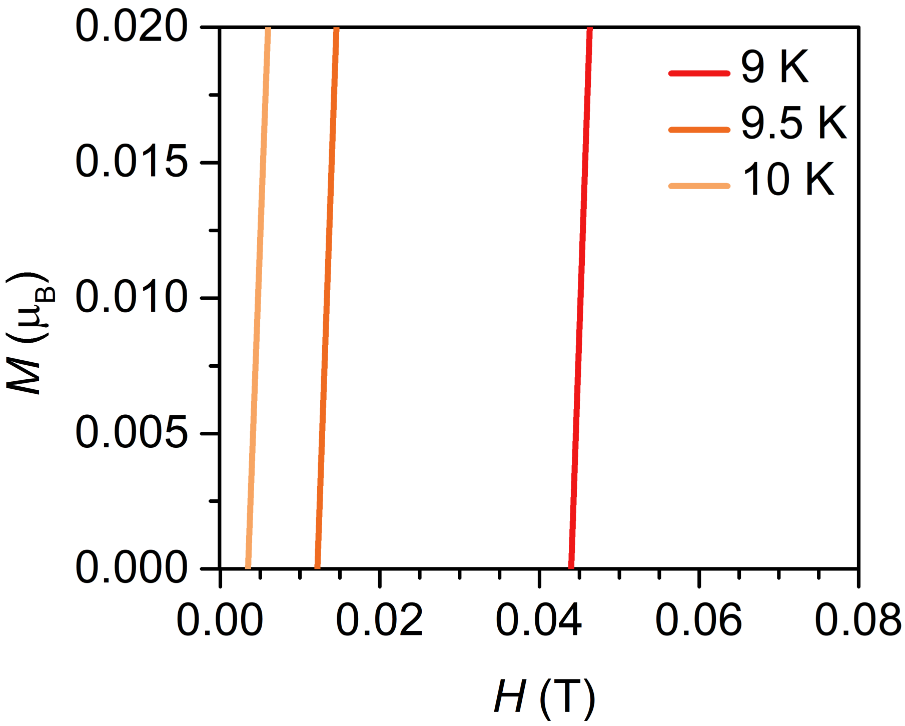

Figure S49. Magnified plot of magnetization (*M*) vs. dc magnetic field (*H*) at an average sweep rate of 0.01 T/s for **3-Dy**.

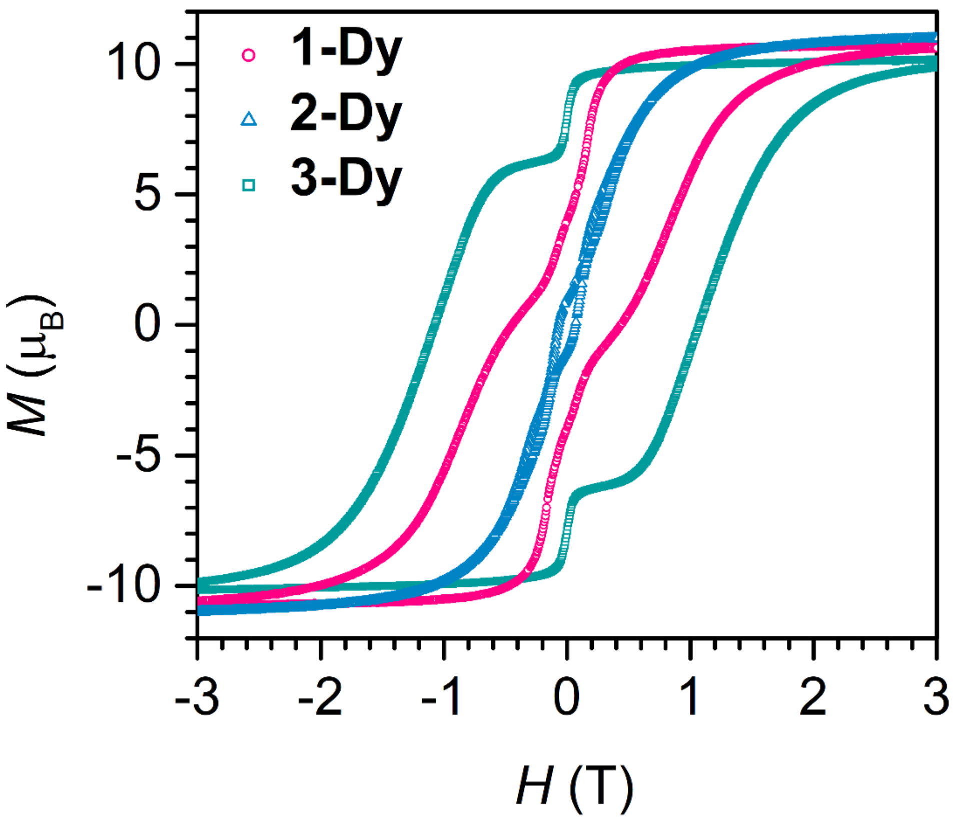

Figure S50. Plot of magnetization (*M*) vs. dc magnetic field (*H*) at an average sweep rate of 0.01 T/s for **1-Dy** (pink circles), **2-Dy** (blue triangles), and **3-Dy** (teal squares) at 2 K.

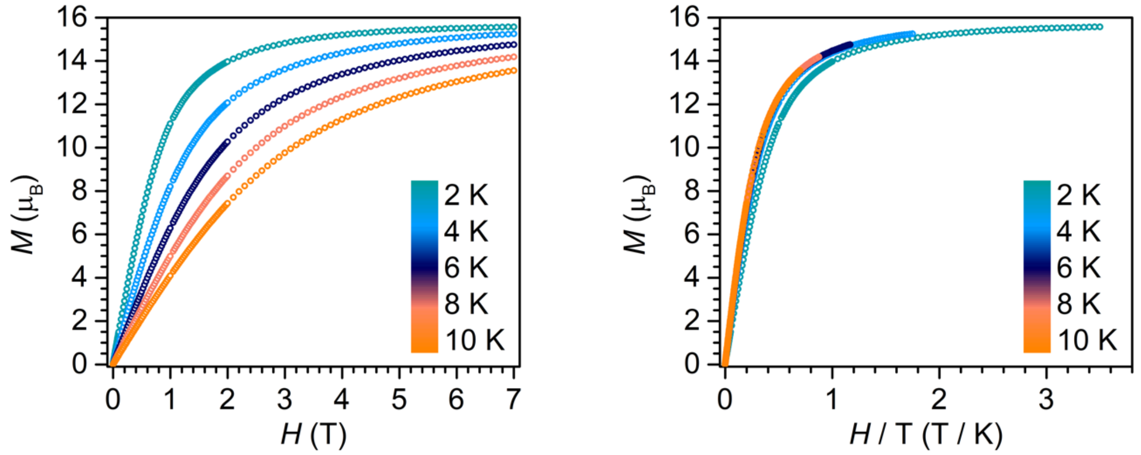

Figure S51. Variable-temperature field-dependent magnetization curves recorded for **1-Gd**, collected from 0 to 7 T (left). Reduced magnetization *M* vs *H*/*T* data for **1-Gd** (right). Measurements were carried out from 0 to 7 T at 2, 4, 6, 8, and 10 K.

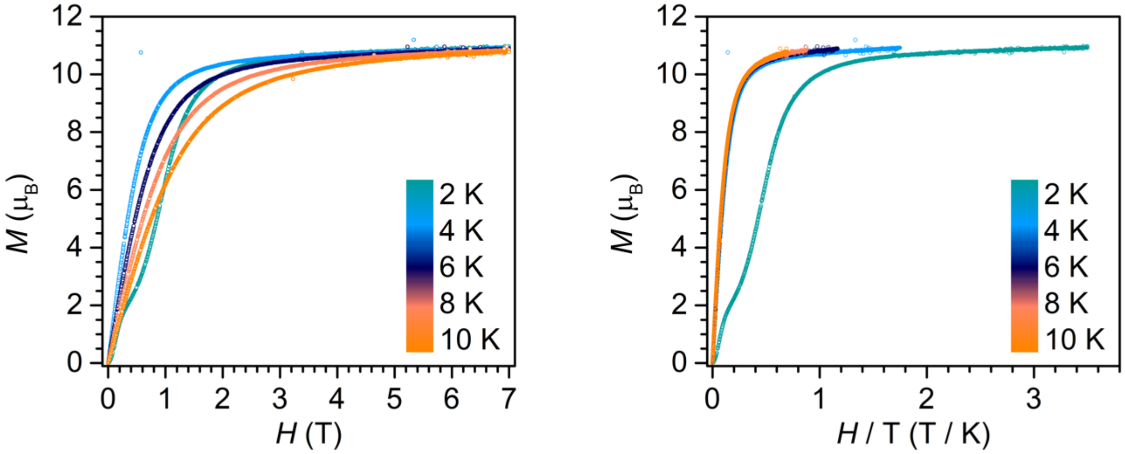

Figure S52. Variable-temperature field-dependent magnetization curves recorded for **1-Dy**, collected from 0 to 7 T (left). Reduced magnetization *M* vs *H*/*T* data for **1-Dy** (right). Measurements were carried out from 0 to 7 T at 2, 4, 6, 8, and 10 K.

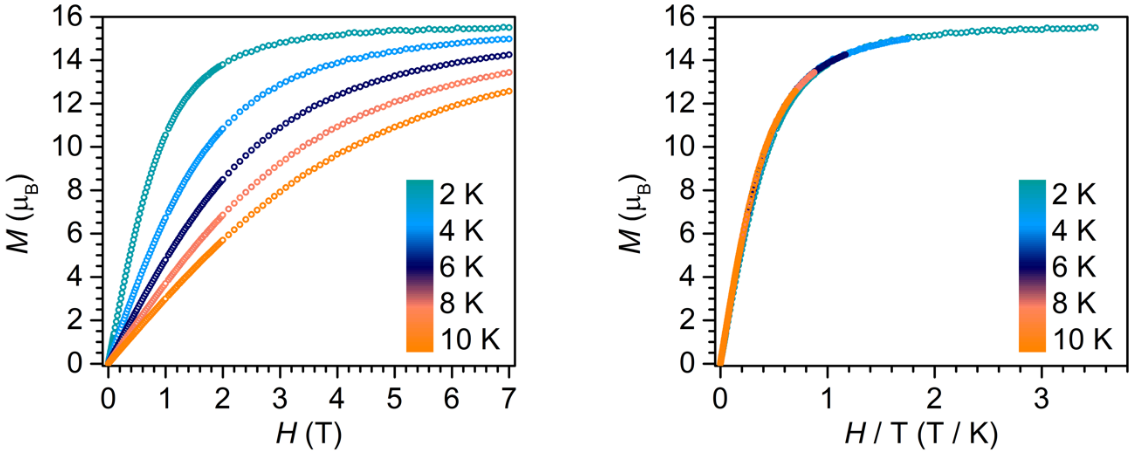

Figure S53. Variable-temperature field-dependent magnetization curves recorded for **2-Gd**, collected from 0 to 7 T (left). Reduced magnetization *M* vs *H*/*T* data for **2-Gd** (right). Measurements were carried out from 0 to 7 T at 2, 4, 6, 8, and 10 K.

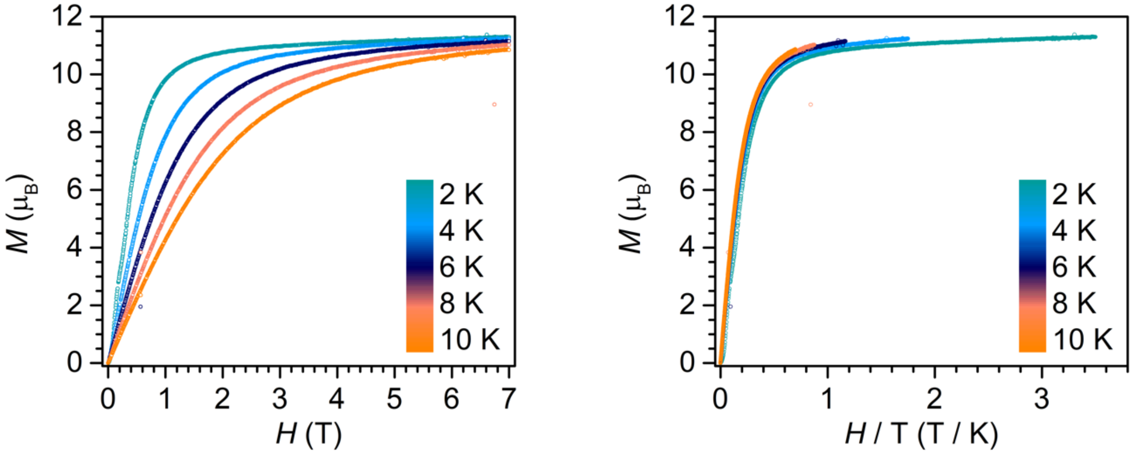

Figure S54. Variable-temperature field-dependent magnetization curves recorded for **2-Dy**, collected from 0 to 7 T (left). Reduced magnetization data of **2-Dy** (right). Measurements were carried out from 0 to 7 T at 2, 4, 6, 8, and 10 K.

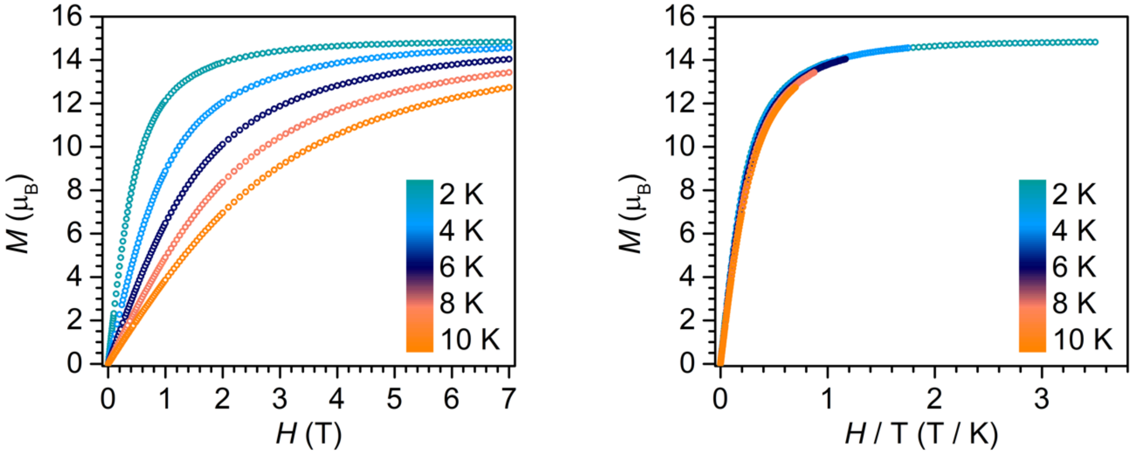

Figure S55. Variable-temperature field-dependent magnetization curves recorded for **3-Gd**, collected from 0 to 7 T (left). Reduced magnetization *M* vs *H*/*T* data for **3-Gd** (right). Measurements were carried out from 0 to 7 T at 2, 4, 6, 8, and 10 K.

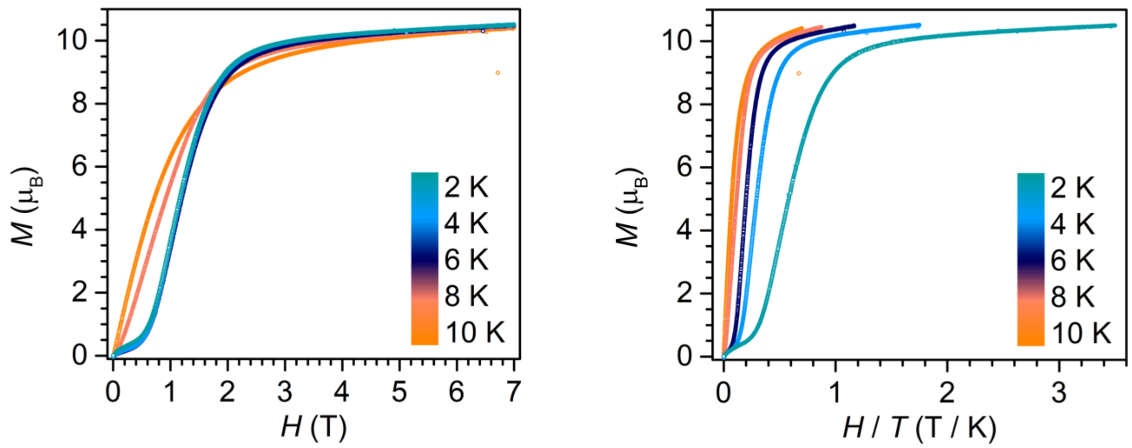

Figure S56. Variable-temperature field-dependent magnetization curves recorded for **3-Dy**, collected from 0 to 7 T (left). Reduced magnetization *M* vs *H*/*T* data for **3-Dy** (right). Measurements were carried out from 0 to 7 T at 2, 4, 6, 8, and 10 K.

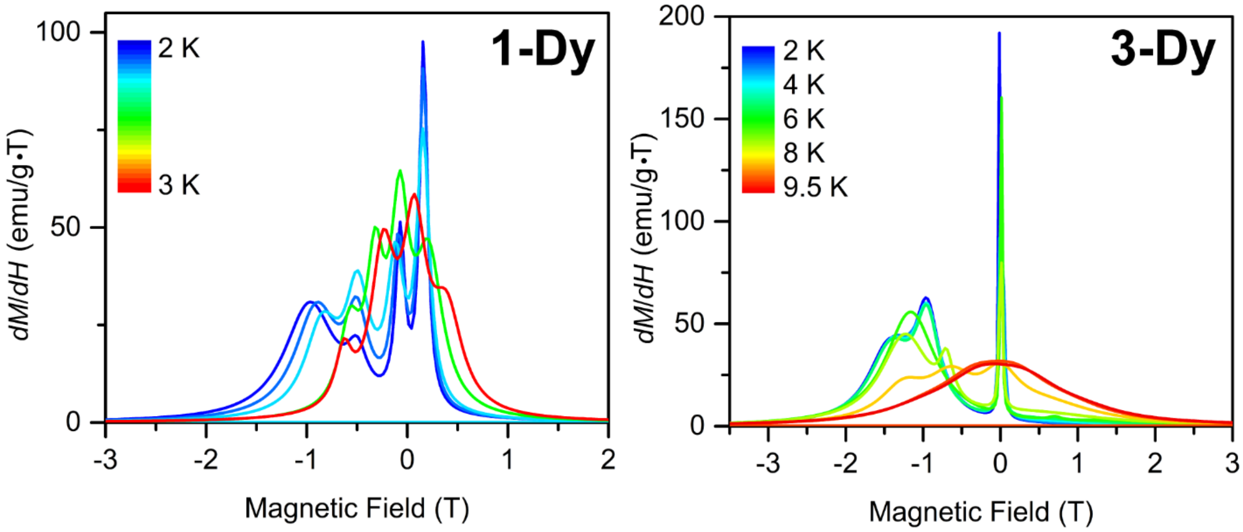

Figure S57. First derivative plots of the fits for the reverse sweep (7 T to –7 T) of the magnetic hysteresis loops of **1-Dy** from 2 K to 3 K (left), and **3-Dy** from 2 K to 9.5 K (right).

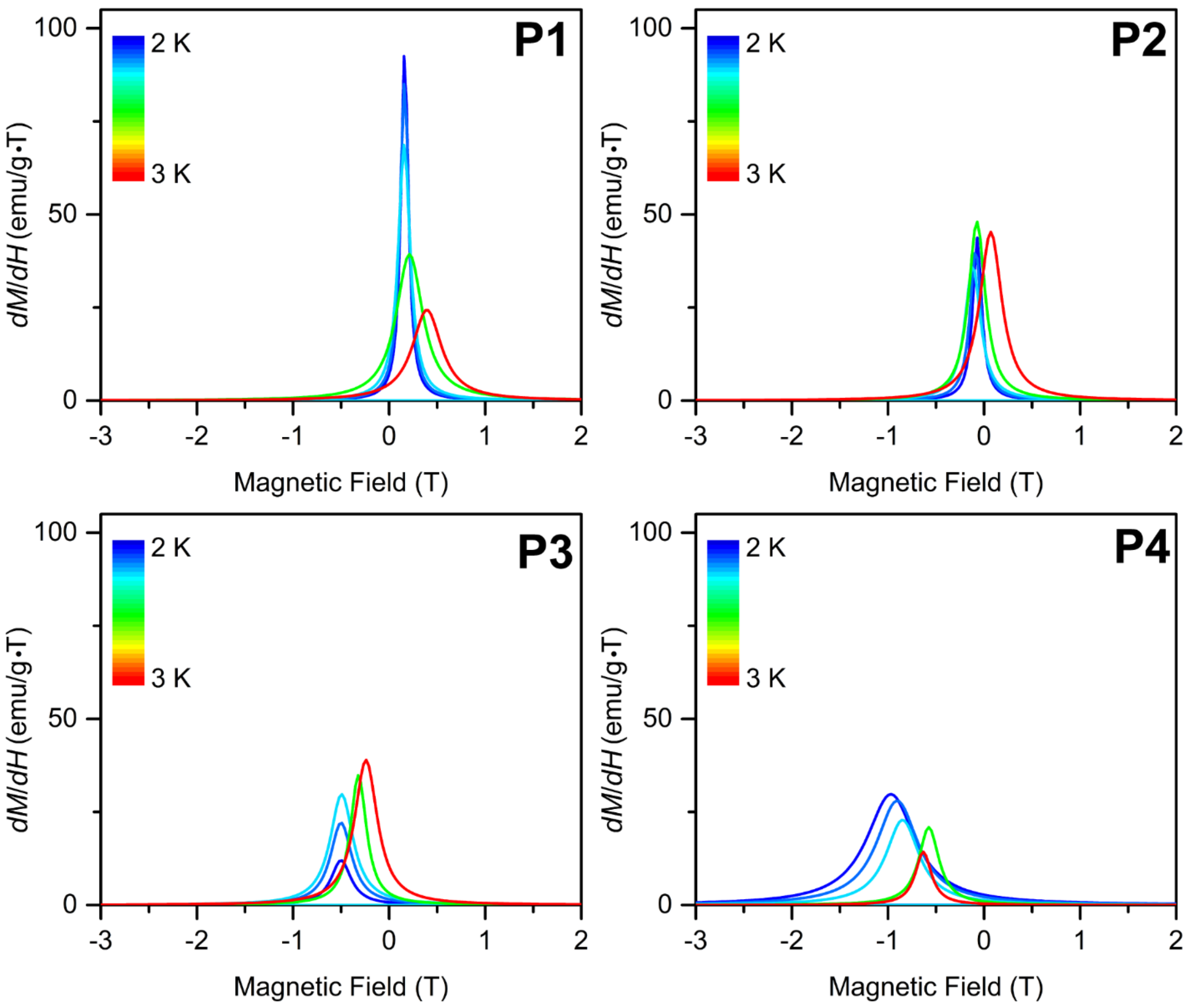

Figure S58. Temperature dependence of the four distinct demagnetization processes identified in the first derivative of the magnetic hysteresis of **1-Dy** through Cauchy probability distribution function analysis. The four processes are labeled as P1, P2, P3, and P4 and their variation in peak position on the field and peak amplitude are plotted from 2 K to 3 K.

Table S9. Cauchy probability distribution function analyses data of the four distinct demagnetization processes determined in the first derivative of magnetic hysteresis of **1-Dy** from 2 K to 3 K (γ = half-width at half-maximum, PC = percent contribution, F = field position). Fit parameters are for the arctan function fitting of the experimental magnetic hysteresis data.

**Temp. (K)**	**Demagnetization Processes**				**Fit Parameters**	
	**P1**	**P2**	**P3**	**P4**	**R^2**	**Reduced χ^2**
**2.0**	γ: 0.046 T<br>PC: 24.8%<br>F: 0.162 T	γ: 0.056 T<br>PC: 14.0%<br>F: –0.070 T	γ: 0.128 T<br>PC: 8.7%<br>F: –0.500 T	γ: 0.309 T<br>PC: 52.4%<br>F: –0.971 T	1.0000	0.0301
**2.1**	γ: 0.056 T<br>PC: 26.4%<br>F: 0.159 T	γ: 0.071 T<br>PC: 15.9%<br>F: –0.087 T	γ: 0.136 T<br>PC: 16.7%<br>F: –0.500 T	γ: 0.264 T<br>PC: 40.9%<br>F: –0.905 T	0.9999	0.0741
**2.2**	γ: 0.076 T<br>PC: 29.0%<br>F: 0.157 T	γ: 0.099 T<br>PC: 20.0%<br>F: –0.117 T	γ: 0.146 T<br>PC: 24.2%<br>F: –0.494 T	γ: 0.211 T<br>PC: 26.8%<br>F: –0.849 T	1.0000	0.0224
**2.5**	γ: 0.168 T<br>PC: 36.9%<br>F: 0.212 T	γ: 0.106 T<br>PC: 28.5%<br>F: –0.074 T	γ: 0.102 T<br>PC: 19.8%<br>F: –0.319 T	γ: 0.127 T<br>PC: 14.8%<br>F: –0.575 T	0.9999	0.0371
**3.0**	γ: 0.186 T<br>PC: 25.1%<br>F: 0.393 T	γ: 0.141 T<br>PC: 35.4%<br>F: 0.070 T	γ: 0.145 T<br>PC: 31.4%<br>F: –0.241 T	γ: 0.102 T<br>PC: 8.1%<br>F: –0.633 T	0.9998	0.1181

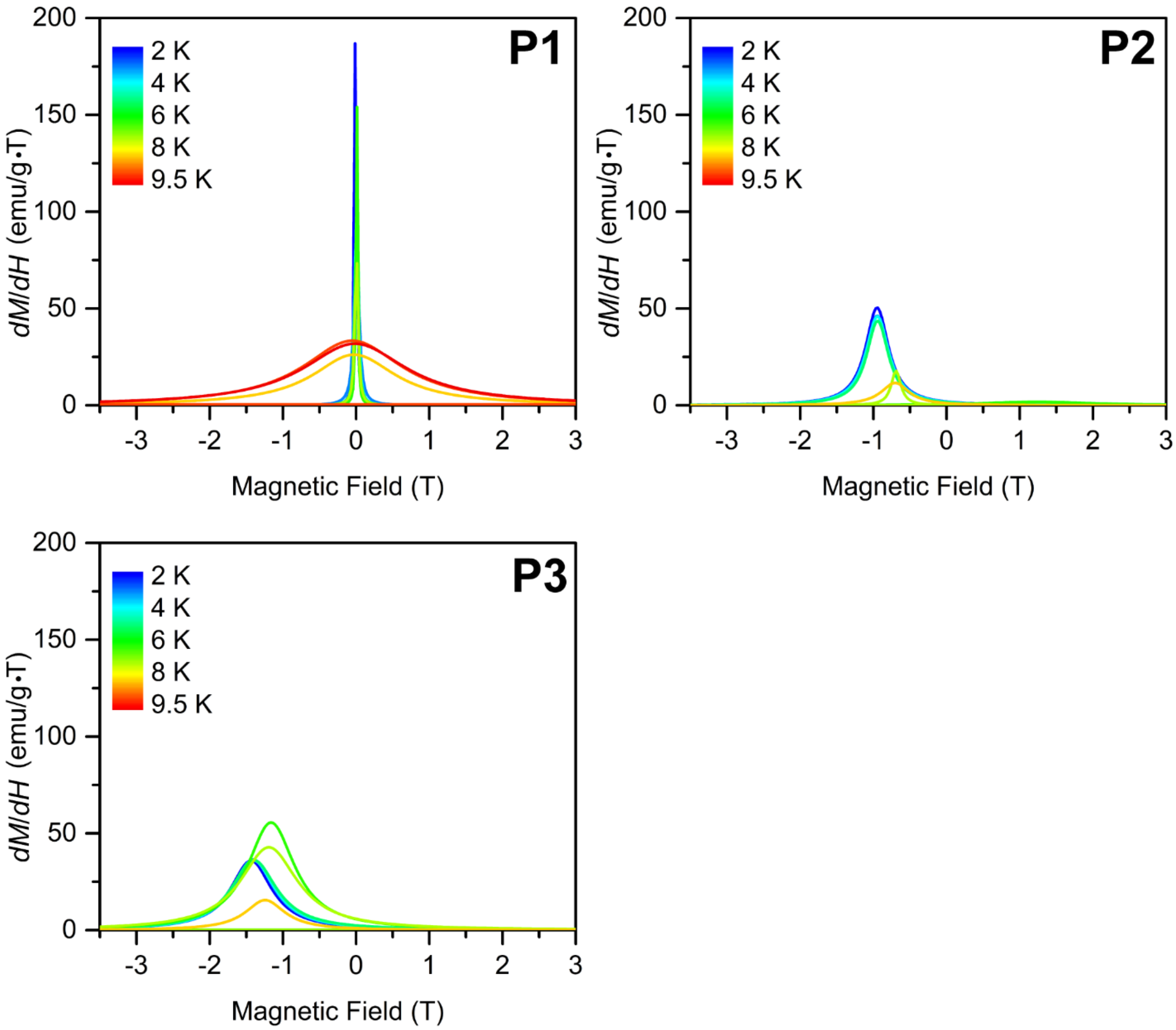

Figure S59. Temperature dependence of the three distinct demagnetization processes identified in the first derivative of the magnetic hysteresis of **3-Dy** through Cauchy probability distribution function analysis. The three processes are labeled as P1, P2, and P3 and their variation in peak position on the field and peak amplitude are plotted from 2 K to 9.5 K. Only P1 shows presence in the full temperature range while P2 and P3 contributions disappear after 8 K.

Table S10. Cauchy probability distribution function analyses data of the four distinct demagnetization processes determined in the first derivative of magnetic hysteresis of **3-Dy** from 2 K to 9.5 K (γ = half-width at half-maximum, PC = percent contribution, F = field position). Fit parameters are for the arctan function fitting of the experimental magnetic hysteresis data.

Temp. (K)	Demagnetization Processes			Fit Parameters	
	P1	P2	P3	R^2	Reduced χ^2
**2.0**	γ: 0.014 T<br>PC: 12.9%<br>F: –0.007 T	γ: 0.192 T<br>PC: 38.3%<br>F: –0.950 T	γ: 0.343 T<br>PC: 48.7%<br>F: –1.436 T	1.0000	0.0548
**3.0**	γ: 0.036 T<br>PC: 14.1%<br>F: –0.006 T	γ: 0.195 T<br>PC: 35.8%<br>F: –0.948 T	γ: 0.347 T<br>PC: 50.1%<br>F: –1.407 T	1.0000	0.0477
**4.0**	γ: 0.021 T<br>PC: 11.5%<br>F: –0.008 T	γ: 0.190 T<br>PC: 35.4%<br>F: –0.945 T	γ: 0.361 T<br>PC: 53.1%<br>F: –1.408 T	0.9998	0.1987
**5.0**	γ: 0.022 T<br>PC: 10.4%<br>F: 0.006 T	γ: 0.182 T<br>PC: 32.8%<br>F: –0.943 T	γ: 0.379 T<br>PC: 56.8%<br>F: –1.398 T	1.0000	0.0565
**6.0**	γ: 0.011 T<br>PC: 7.8%<br>F: 0.009 T	γ: 0.943 T<br>PC: 6.5%<br>F: –1.233T	γ: 0.394 T<br>PC: 85.6%<br>F: –1.163T	0.9999	0.1610
**7.0**	γ: 0.024 T<br>PC: 8.5%<br>F: 0.005 T	γ: 0.079 T<br>PC: 5.9%<br>F: –0.691 T	γ: 0.476 T<br>PC: 85.6%<br>F: –1.193 T	0.9999	0.0724
**8.0**	γ: 0.695 T<br>PC: 12.9%<br>F: –0.007 T	γ: 0.192 T<br>PC: 38.3%<br>F: –0.950 T	γ: 0.343 T<br>PC: 48.7%<br>F: –1.436 T	1.0000	0.0177
**9.0**	γ: 0.872 T<br>PC:<br>100.0%<br>F: –0.017 T	-	-	1.0000	0.0042
**9.5**	γ: 0.833 T<br>PC:<br>100.0%<br>F: –0.051 T	-	-	0.9948	0.3887

5 TD-DFT Calculations

Table S11. Majority contributions of the TD-DFT-calculated transition states for **1-Gd** on the def2-TZVP level on all atoms, SARC2-ZORA-QZVP on Gd atoms and ZORA-def2-TZVPP on flv atoms using the uB3LYP functional with D3BJ dispersion correction and DCM implicit solvent model. The calculated excitation energies were empirically shifted by 0.25 eV. Isovalue for all depictions is 0.03. Oscillator strength cutoff used is 0.05 and contributions higher than 15% are shown. (HOMO = 280, LUMO = 281)

λ (nm)	ν (cm^{-1})	Oscillator Strength	Occupied	Virtual	Weight (%)
255.0	39220	0.88841	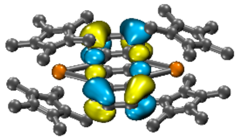 271α	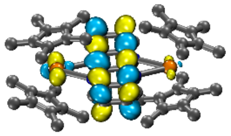 282α	33.2
			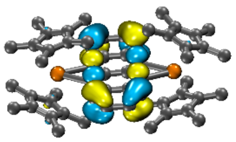 257β	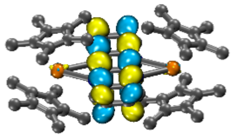 268β	20.7
429.3	23293	0.40854	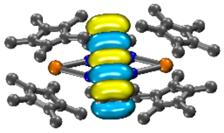 255β	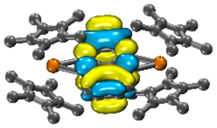 266β	59.5
377.4	26496	0.08923	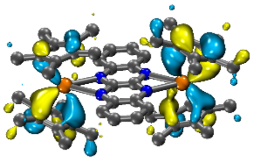 261β	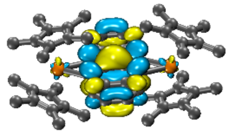 267β	95.8
429.8	23268	0.07753	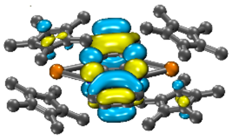 280α	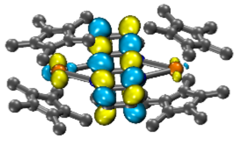 282α	69.9

255.7	39114	0.072269	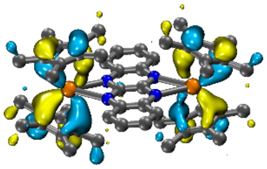 260β	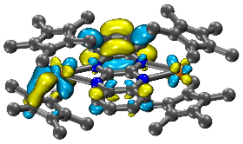 272β	30.9
414.3	24135	0.06067	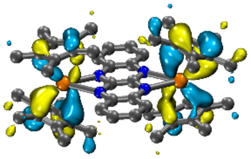 275α	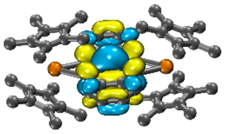 281α	45.8
867.0	11533	0.05902	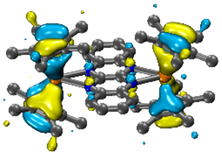 259β	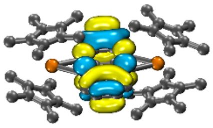 266β	97.8
255.5	39140	0.05811	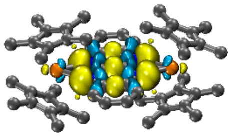 254β	267β	48.2
255.3	39164	0.05519	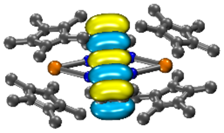 269α	281α	23.6
			255β	267β	24.8

Table S12. Majority contributions of the TD-DFT-calculated transition states for **2-Gd** on the def2-TZVP level on all atoms, SARC2-ZORA-QZVP on Gd atoms and ZORA-def2-TZVPP on flv atoms using the uB3LYP functional with D3BJ dispersion correction and THF implicit solvent model. The calculated excitation energies were empirically shifted by 0.37 eV. Isovalue for all depictions is 0.03. Oscillator strength cutoff used is 0.05 and contributions higher than 15% are shown. (HOMO = 280, LUMO = 281)

λ (nm)	ν (cm^{-1})	Oscillator Strength	Occupied	Virtual	Weight (%)
250.3	39958	0.37114	No individual transitions above 15%		
434.6	23009	0.29557	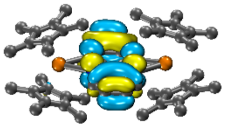 280α	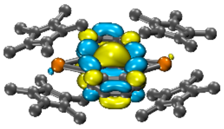 281α	24.9
			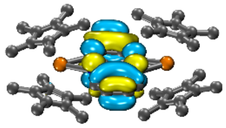 266β	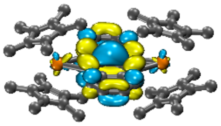 267β	26.6
			280α	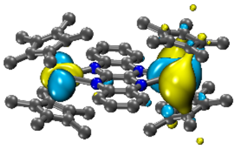 282α	41.0
250.1	39978	0.19768	No individual transitions above 15%		
441.9	22629	0.16034	266β	267β	18.6
			280α	281α	21.6
			280α	282α	53.3
250.1	39989	0.15293	No individual transitions above 15%		
258.1	38749	0.09489	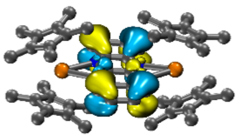 256β	267β	19.7

			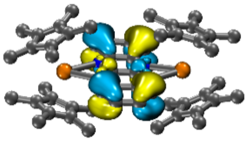 270α	281α	20.7
267.5	37381	0.09151	No individual transitions above 15%		
278.0	35969	0.08284	No individual transitions above 15%		
354.0	28251	0.08168	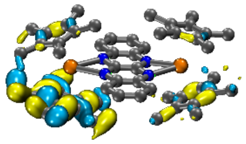 261β	267βα	37.6
			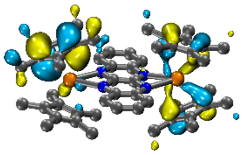 275α	281α	24.8
277.9	35979	0.06621	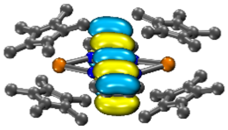 269α	281α	20.6
			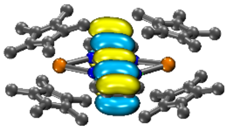 255β	267β	23.5
331.5	30162	0.06259	280α	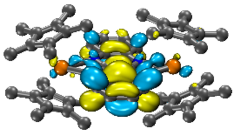 285α	30.2
			266β	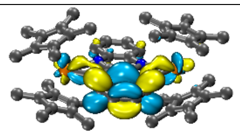 271β	37.2

Table S13. Majority contributions of the TD-DFT-calculated transition states for **3-Gd** on the def2-TZVP level on all atoms, SARC2-ZORA-QZVP on Gd atoms and ZORA-def2-TZVPP on flv atoms using the uB3LYP functional with D3BJ dispersion correction and THF implicit solvent model. The calculated excitation energies were empirically shifted by 0.75 eV. Isovalue for all depictions is 0.03. Oscillator strength cutoff used is 0.04 and contributions higher than 15% are shown. (HOMO = 281, LUMO = 282)

λ (nm)	ν (cm^{-1})	Oscillator Strength	Occupied	Virtual	Weight (%)
265.3	37695	0.14719	No individual transitions above 15%		
449.6	22242	0.13821	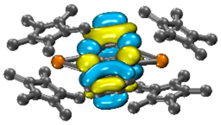 266β	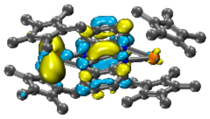 268β	73.6
266.5	37523	0.08187	No individual transitions above 15%		
264.2	37851	0.07504	No individual transitions above 15%		
468.0	21368	0.07173	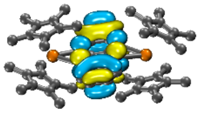 280α	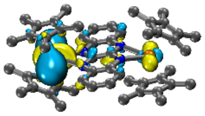 283α	17.2
			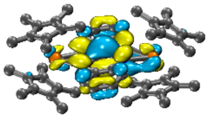 281α	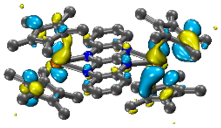 304α	24.2
			266β	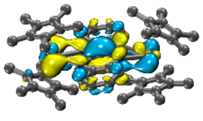 267β	34.2
273.1	36621	0.04592	266β	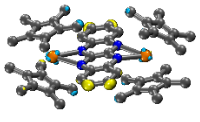 298β	35.8
324.5	30814	0.04455	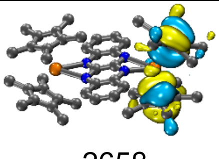 265β	267β	20.2

			265β	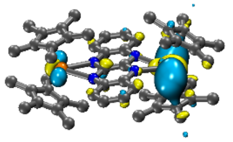 269β	45.1
360.0	27777	0.04284	280α	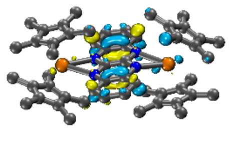 288α	15.3
			281α	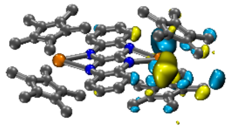 315α	22.2
265.6	37657	0.04246	No individual transitions above 15%		

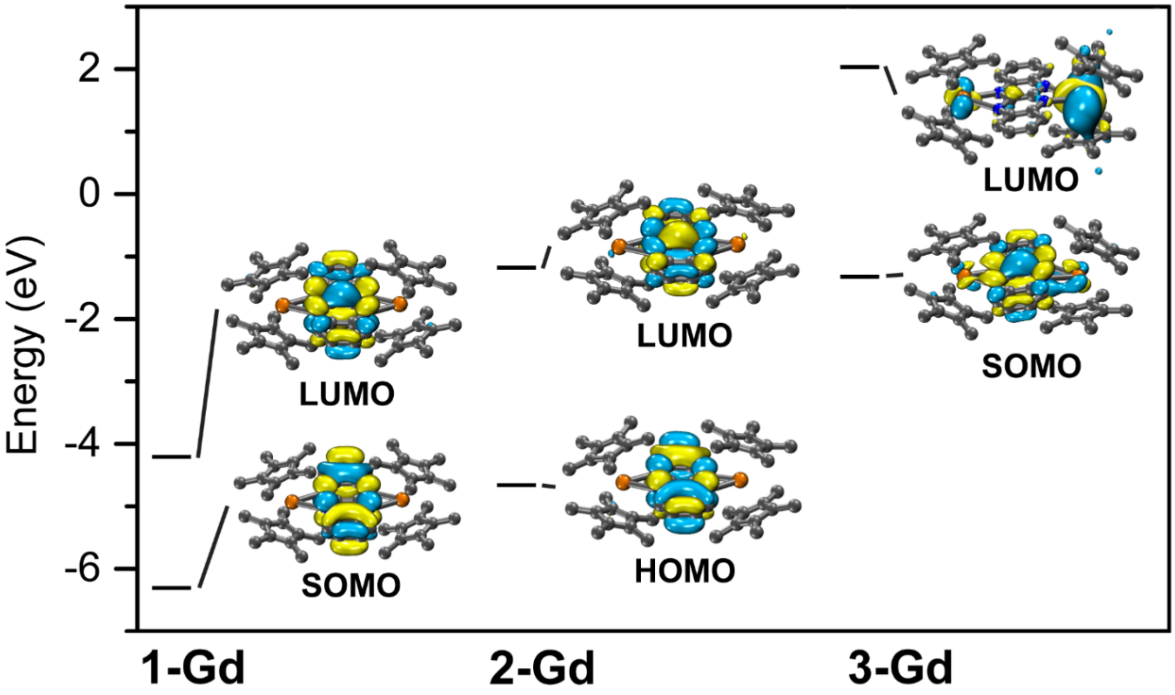

Figure S60. Calculated frontier molecular orbitals of **1-Gd** (left), **2-Gd** (center), and **3-Gd** (right). Molecular orbitals (MOs) are depicted with cyan and yellow surfaces. Orange, blue, and gray spheres represent Gd, N, and C atoms. N atoms are covered by molecular orbital isosurfaces. The lower MO pictures depict the highest singly occupied molecular orbitals for **1-Gd** and **3-Gd** (SOMOs), and highest doubly occupied molecular orbital for **2-Gd** (HOMO). The upper MO pictures depict the lowest unoccupied molecular orbital (LUMO) for each molecule. Isovalues used for depiction of all surfaces is 0.003.

6 Broken-Symmetry DFT

Table S14. Results of the broken-symmetry DFT calculations performed on the crystal coordinates of $[(Cp^*_2Gd)_2(\mu\text{-flv}^{\bullet})]^+$ in **1-Gd**, using multiple functionals. SARC2-ZORA-QZVP basis was used for Gd atoms and ZORA-def2-TZVPP was used for all flv atoms while ZORA-def2-TZVP basis was used for all atoms. The calculation employed D3BJ dispersion correction and SARC/J auxiliary basis set. Spin on all flv atoms were flipped with the spinflip function. EPR parameters were calculated with the EPRNMR block.

Functional	*g* values	*D* (cm^{-1})	*E*/*D*	*J* (cm^{-1})
B3LYP	1.9988926<br>2.0034305<br>2.0116698<br>2.0046643 (iso)	0.602776	0.282011	–14.35
CAM-B3LYP	1.987519<br>2.0038530<br>2.0093771<br>2.0039940 (iso)	0.604000	0.112650	–14.08
PBE0	1.9977108<br>2.0032888<br>2.0106874<br>2.0038956 (iso)	0.521020	0.225528	–13.81
REVPBE38	1.9854071<br>1.9994912<br>2.0029304<br>1.9959429 (iso)	–18.638363	0.011315	–1528.44
TPSS0	1.9974033<br>2.0032549<br>2.0106858<br>2.0037814 (iso)	–0.350724	0.302480	–14.01
TPSSh	1.9989384<br>2.0053665<br>2.0160510<br>2.0067853 (iso)	–1.225620	0.068877	–16.28
wB97M-D3BJ	1.9975384<br>2.0044869<br>2.0085485<br>2.0035246 (iso)	–0.171818	0.217755	–13.96

Table S15. Results of the broken-symmetry DFT calculations performed on the crystal coordinates of [(Cp*$_2$Gd)$_2$(μ-flv)], **2-Gd**, using multiple functionals. SARC2-ZORA-QZVP basis was used for Gd atoms and ZORA-def2-TZVPP was used for all flv atoms while ZORA-def2-TZVP basis was used for all atoms. The calculation employed D3BJ dispersion correction and SARC/J auxiliary basis set. Spin on one Gd center was flipped with the spinflip function.

Functional	*J* (cm^{-1})
B3LYP	–0.04
CAM-B3LYP	–0.01
REVPBE38	–0.01
TPSS0	–0.02

Table S16. Results of the broken-symmetry DFT calculations performed on the crystal coordinates of [(Cp*$_2$Gd)$_2$(μ-flv$^•$)]$^–$ in **3-Gd**, using multiple functionals. SARC2-ZORA-QZVP basis was used for Gd atoms and ZORA-def2-TZVPP was used for all flv atoms while ZORA-def2-TZVP basis was used for all atoms. The calculation employed D3BJ dispersion correction and SARC/J auxiliary basis set. Spin on all flv atoms were flipped with the spinflip function. EPR parameters were calculated with the EPRNMR block.

Functional	*g* values	*D* (cm^{-1})	*D*/*E*	*J* (cm^{-1})
B3LYP	1.9973741<br>2.0041935<br>2.0154863<br>2.0056846 (iso)	–0.995540	0.122891	–18.98
CAM-B3LYP	1.9952630<br>2.0026742<br>2.0134439<br>2.0037937 (iso)	–0.283850	0.128417	–14.60
PBE0	1.9963037<br>2.0036576<br>2.0152326<br>2.0050646 (iso)	–0.304691	0.212380	–11.23
REVPBE38	1.9963099<br>2.0017794<br>2.0131191<br>2.0037361 (iso)	1.272521	0.062310	–5.78
TPSS0	1.9969369<br>2.0030297<br>2.0152579<br>2.0050749 (iso)	–0.253105	0.189067	–8.82
TPSSh	2.0013234<br>2.0084406<br>2.0255475<br>2.0117705 (iso)	–0.750199	0.061367	–32.87
wB97M-D3BJ	1.9945884<br>2.0030348<br>2.0157845<br>2.0044692 (iso)	–0.235789	0.090086	–14.67

Table S17. Mulliken spin populations on the N atoms in flv ligands for $[(Cp^*_2Gd)_2(\mu\text{-flv}^\bullet)]^+$ in **1-Gd**, and $[(Cp^*_2Gd)_2(\mu\text{-flv}^\bullet)]^-$ in **3-Gd**, obtained following the broken-symmetry calculations with the TPSS0 functional. SARC2-ZORA-QZVP basis was used for Gd atoms and ZORA-def2-TZVPP was used for all flv atoms while ZORA-def2-TZVP basis was used for all atoms. The calculation employed D3BJ dispersion correction and SARC/J auxiliary basis set. Spin on all flv atoms were flipped with the spinflip function.

Compound	Mulliken Spin Populations			
	N1	N2	N3	N4
**1-Gd**	-0.181542	-0.194293	-0.181259	-0.194347
**3-Gd**	-0.129001	-0.144741	-0.127771	-0.142695

7 High-field Electron Paramagnetic Resonance

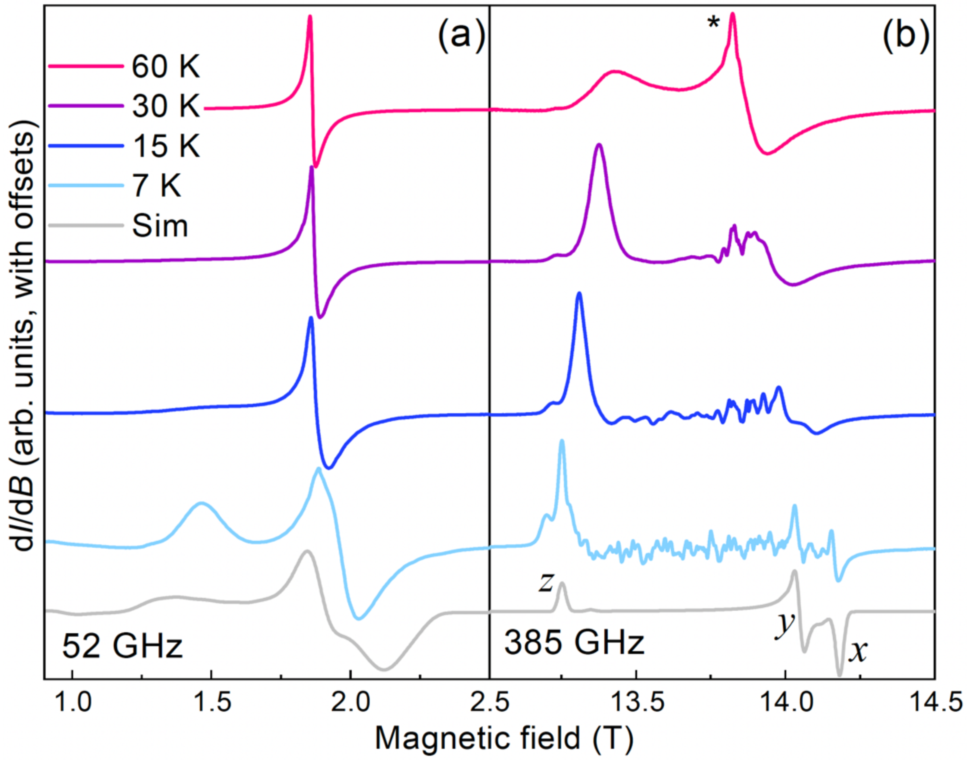

Figure S61. Temperature-dependent powder EPR spectra collected for **1-Gd** at frequencies of 52 GHz (a) and 385 GHz (b); the spectra are recorded in derivative mode, d*I*/d*B*, where *I* is the microwave intensity transmitted through the sample and *B* is the local magnetic field; see legend for corresponding temperatures. Simulations ("Sim" – gray lines) according to Eq. (8) in the main text are shown below the corresponding experimental spectra for the lowest temperature at both frequencies. These simulations were generated using a single set of spin Hamiltonian parameters given in Table S18. The asterisk denotes a broad radical signal at high temperatures, suggesting decoupling of the spins above 30 K.

Figure S61 displays temperature-dependent powder EPR spectra for **1-Gd** at frequencies of 52 and 385 GHz. The quality of these spectra is not as high as those obtained for **3-Gd** (see Figure 13 in main text). One reason is that the powder sample was not sufficiently ground. This can be seen clearly in the lowest temperature spectra at 385 GHz from what looks like noise in between the *z* and *xy* resonances. In fact, this apparent "noise" is way above the true noise level of the spectrometer. Because the sample resonantly absorbs in the entire region between *z* and *x* (even as $T \rightarrow 0$), larger crystallites within the powder that are randomly oriented with respect to the applied field can cause spikes in EPR intensity and, hence, in d*I*/d*B*. This apparent "noise" therefore obscures any of the real signals in between the *x*, *y* and *z* turning points, both at 7 K and 15 K. However, one can still clearly identify the three components of the $m_S = -^7/_2$ to $-^5/_2$ ground state transition. This provides sufficient information to constrain *g*, *D* and *E* (but not B_4^0) in Eq. (8) of the main text; these values are given in Table S18 and the best simulation is shown below the 7 K spectrum in Figure S61(b). The obtained parameterization was then used to simulate the lowest temperature 52 GHz spectrum, which is relatively featureless. This suggests an additional broadening mechanism at low fields, which could be due to dipolar field fluctuations within the sample (combined with the effects of insufficient sample grinding), as the spins are only partially polarized in this *B*,*T* regime. The simulations are not perfect, but do capture the strong central resonance, with broad resonances either side. Lastly, we note the appearance of a broad radical signal in the 60 K spectrum at 385 GHz. Similar to **3-Gd**, this suggests decoupling of the Gd^{III} spins from the radical, albeit at a higher temperature, reflecting the stronger coupling in **1-Gd**.

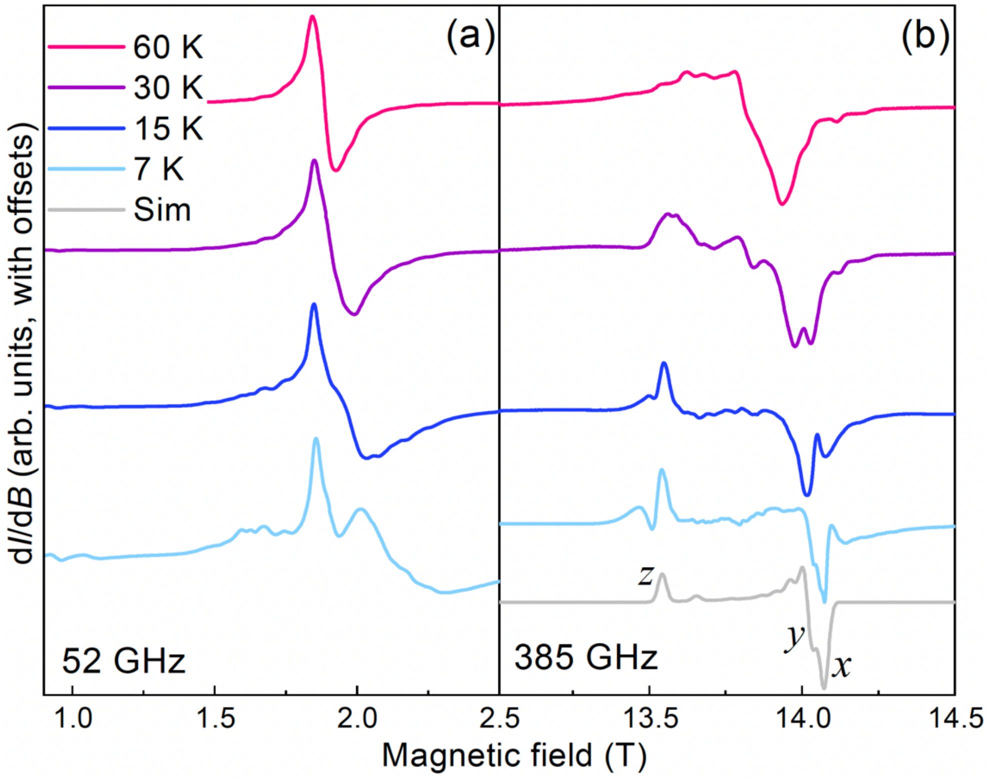

Figure S62. Temperature-dependent powder EPR spectra collected for **2-Gd** at frequencies of 52 GHz (a) and 385 GHz (b); see legend for corresponding temperatures. A simulation ("Sim" – gray line) according to Eq. (8) in the main text is shown below the 385 GHz, 7 K spectrum. The obtained spin Hamiltonian parameters are given in Table S18.

Figure S62 displays temperature-dependent powder EPR spectra for **2-Gd** at frequencies of 52 and 385 GHz. The quality of these spectra is not as high as those obtained for **3-Gd** (see Figure 13 in main text). However, one can still observe the ground state $m_S = -^7/_2$ to $-^5/_2$ *x*, *y* and *z* transitions. This provides sufficient information to constrain *g*, *D* and *E* (but not B_4^0) in Eq. (8) of the main text; these values are given in Table S18 and the best simulation is shown below the 7 K spectrum in Figure S62(b). We note, however, that a spin $S = ^7/_2$ Hamiltonian was employed in this case, reflecting the expected uncoupled nature of **2-Gd** and the absence of a radical. The overall span of the spectrum is about half of that for **1-Gd**, giving some hits that the overall spin state is lower. One also observes some features in the 15 K spectra, but only a few on either side of the strong central $m_S = -^1/_2$ to ½ transition at 52 GHz. Again, this appears to confirm the lower spin value of $S = ^7/_2$. Finally, we note that there is no radical signal in the 385 GHz spectrum at the highest temperature, confirming the absence of the radical bridge.

Table S18. Comparison of spin-Hamiltonian parameters deduced from EPR for all samples.

Sample	*S*	g_{iso}	*D* (cm^{-1})	*E*/*D*	B_4^0 (× 10^{-6} cm^{-1})
**1-Gd**	$^{13}/_2$	1.990	–0.0444	0.079	-
**2-Gd**	$^7/_2$	1.982	–0.0517	0.055	-
**3-Gd**	$^{13}/_2$	1.987	–0.0370	0.117	1.67

8 Coordinates of Optimized Structures

Cartesian coordinates of the geometry optimized structure of the $[(Cp^*{}_2Gd)_2(\mu\text{-flv}^{\bullet})]^+$ cation in **1-Gd**, at def2-TZVP level using the uTPSSh functional, SARC/J auxiliary basis set and D3BJ dispersion correction. (Final single point energy = –3850.402126576428 Hartrees)

Gd	11.41656240334952	16.78036697580494	7.73028207373332
N	10.97165564582354	14.28966084533764	7.61339360683389
N	12.54868181922815	15.13134177228449	6.18515678390229
C	8.81784907410680	16.91675442231715	6.82011182949319
C	12.04873945263169	16.65589902314568	10.36446936179806
C	12.82008616311792	15.57446151817214	9.88331964646844
C	10.10279605579662	18.82664583363535	6.80239736803792
C	10.40811022441775	18.07522863176504	5.62850462542996
C	9.61945558133685	16.90045318470451	5.64482986944154
C	13.81395441640242	16.08478964464737	9.00026997231200
C	9.11443995862461	18.09347507822343	7.54592953677986
C	13.66857412278542	17.48830629488323	8.93260322860811
C	12.57881969203379	17.85604879443427	9.79758094905620
C	11.92233245390218	14.06398296542546	6.69683594105762
C	10.34123525449586	13.21088699805562	8.15063912736459
C	13.52103565637566	14.91250387419768	5.26066250175577
C	12.26906198068908	12.72981474287363	6.29258902361442
C	14.21116239409318	16.00023943816634	4.69149278472616
C	13.86407043304972	13.57885012654241	4.85317717311156
C	9.35174208596765	13.38820523812811	9.13698975375684
C	8.71035449528764	12.30273764817746	9.68577598334323
C	9.03371714132437	10.99461392391087	9.26903852596801
C	7.76877401235773	15.90425445998324	7.16750304324821
C	15.19232509685730	15.78931421843649	3.75105575026459
C	10.95845812788602	16.58632725247177	11.39332954311464
C	12.71219992446293	14.14611350664600	10.31759671945272
C	10.57743907641531	20.21704216370233	7.09623766422315
C	11.33687412425059	18.51766270626331	4.53692787108013
C	10.67663011982841	11.87834975849277	7.73323796257357
C	9.57428370755560	15.84334252223829	4.58446181493374
C	14.89683389765671	15.26714435016274	8.36507086251092
C	15.52194606126005	14.48002771201412	3.34335457917862
C	8.42102193959831	18.57259841511934	8.78684666624448
C	14.56853092868554	18.44631745333575	8.20904432828538
C	12.22801832322151	19.24871507712832	10.21800367249216
H	9.11276800081154	14.39699661553541	9.44851918240927
H	7.95110953309567	12.45019644536790	10.44376691794758
H	13.94638986082873	17.00105527229121	5.00890545242750
H	10.49761483038369	15.81351011366064	4.00377672132643
H	8.75210768708020	16.02132606495692	3.88302813220999
H	9.41436521574807	14.85156860025751	5.01552820016500
H	15.71573510140822	16.63282017226399	3.31817313844873
H	14.53260732023677	14.28359064137427	8.05670548789724
H	15.71789440433701	15.09604886204018	9.06954300721355

H	15.31837563465488	15.75641077971098	7.48582061249526
H	11.71921633128477	13.90924485571619	10.70154158449967
H	13.43049280965355	13.93076759882248	11.11625964706380
H	12.92797936391718	13.45241391368735	9.50217979058048
H	8.07235726024401	14.88979032736358	6.90010073790351
H	6.83627377853706	16.11400568573649	6.63246424878120
H	7.53210782251984	15.91424544647188	8.23344553272548
H	10.19263494537704	17.34899166813554	11.23127385647474
H	11.35555465125646	16.74257900370163	12.40225871845959
H	10.46661845538456	15.61115936743527	11.39152786593546
H	12.21478808218260	19.03971902781325	4.93054582044720
H	10.83793814059951	19.21524029469655	3.85558742809920
H	11.68599881962449	17.67486560844666	3.93788133561209
H	15.04522596425591	17.97544944297139	7.34629223766012
H	15.37160482235802	18.80452860067783	8.86255376928763
H	14.03096639755792	19.33065505095626	7.85598635755526
H	10.46931135585824	20.46990005297977	8.15122351126930
H	9.99220109241071	20.94828328492478	6.52683202038825
H	11.62408955807684	20.36636792128106	6.81679715886346
H	8.08787420527744	17.74226531201661	9.41405370096664
H	7.53169781829867	19.16158457958917	8.53753375461296
H	9.06587188172144	19.21112077260779	9.39430632785598
H	12.47510058817666	19.98534040063096	9.45264063530181
H	12.79075300056541	19.52068330588813	11.11921506409420
H	11.16927747820395	19.35105903445360	10.46508354750655
Gd	12.81881100261025	10.01065380774430	5.29006040397807
N	13.22815757203931	12.50193735566604	5.38603653705968
N	11.63867234967985	11.66222599233795	6.79822479188292
C	14.87070871248542	13.39696031220793	3.88551763874498
C	9.99847265565978	10.78760816732340	8.31115608234505
H	8.51930589191605	10.14957490335382	9.70960580736273
H	16.29454366365486	14.32943503303646	2.59960924251797
C	14.28956925719223	8.03732956133600	6.11617747120166
C	10.73433698840186	9.12736260423264	3.95164791592616
C	11.87374467892183	8.98602638578130	3.07852713266757
C	14.43162835701684	9.85533173535526	7.51705908590824
C	15.34079492814777	10.05680673343694	6.44050648902606
C	15.25102939076288	8.94894377728425	5.56660520893425
C	12.24110587402440	10.28658959207821	2.63149131888436
C	13.78140362588003	8.61458839245183	7.32264282614644
C	11.33057326297870	11.21323144559664	3.19304842675087
C	10.40555800963901	10.50190568148346	4.00592350484643
H	15.11552855249649	12.38653152610008	3.58393591425953
H	10.26157241156297	9.78794260116629	7.98728082894371
C	14.00748948483267	6.64749951562147	5.63432058391123
C	9.94881675578286	8.00016665584258	4.55410972999450
C	12.41692101377188	7.69737995353793	2.54478112696941
C	14.26463137034962	10.78060010931378	8.68312849846898
C	16.29584622653034	11.20547191454721	6.32164129445251
C	16.09313538392843	8.70389735408459	4.34872945399076
C	13.32478284191816	10.58835972036868	1.63843235530825

C	12.80200993658711	7.95415457606078	8.24781311641424
C	11.26091348626057	12.67899460095483	2.89793509252207
C	9.21345814821193	11.10239797591419	4.68736452707963
H	14.25568038535459	6.52297580231201	4.58011952298118
H	14.60698005172259	5.92176481240570	6.19607488570692
H	12.96051357568323	6.36437524300201	5.77348207537173
H	9.44268467447874	8.30585901286036	5.47329511016125
H	9.17207529608354	7.65015121484142	3.86498864797173
H	10.57854656143468	7.13766742076312	4.78634291259239
H	12.25503189973545	6.86494652036912	3.23007582658051
H	11.91712381452219	7.44133052716602	1.60287410466587
H	13.48561860469716	7.75991439220928	2.32735161403207
H	13.29333631984903	10.65937557966407	9.16567968710905
H	15.03274461150885	10.60044753546663	9.44283626127941
H	14.35901883036758	11.82579083463924	8.37806222991005
H	15.84130102471941	12.15062578828669	6.62652135921513
H	17.17195289068031	11.04673554511111	6.95953579246341
H	16.66127538924537	11.32504208474036	5.30050046814828
H	16.36876083975448	9.63714908754300	3.85116661365346
H	17.02629527815931	8.19234739740386	4.60754202367728
H	15.57732888510415	8.07747304075423	3.61749120177588
H	14.21378371745937	9.97157968081153	1.79835424977988
H	12.98618489143374	10.39585426130642	0.61456829976164
H	13.62902261209827	11.63552277607388	1.68349222116039
H	11.98879020202915	7.45850727575602	7.70680936261474
H	13.28996318925997	7.18167314984026	8.85179243397291
H	12.35823380861968	8.67214297966113	8.93955280238674
H	12.21427548251709	13.06804675677701	2.53810775097167
H	10.51167996477359	12.88382341577530	2.12554048289912
H	10.97232965084711	13.25852990654315	3.77775657231474
H	9.40267519945223	12.13166956675616	5.00055968032601
H	8.35186704125102	11.12631884664840	4.01123095511702
H	8.91444941708207	10.53720086497250	5.57186079620622

Cartesian coordinates of the geometry optimized structure of [(Cp*$_2$Gd)$_2$(μ-flv)], **2-Gd**, at def2-TZVP level using the uTPSSh functional, SARC/J auxiliary basis set and D3BJ dispersion correction. (Final single point energy = –3850.598038828176 Hartrees)

Gd	5.03810854849622	2.69357834786725	9.98618037561604
N	3.47947137538728	2.60773657269265	8.12046530076722
N	5.09745481917146	4.23882639560222	8.11446818076066
C	6.29594959833351	0.91694053008967	8.28114862668529
C	7.33218980985894	1.80239863802344	8.68490085578959
C	3.71530367591735	4.92805201174349	10.93521141171524
C	5.85392707892116	0.20518617800629	9.42118439275370
C	2.80689754756127	3.84830657448254	11.09815081619760
C	6.62027493247786	0.65080118566258	10.54321584384507
C	4.83214168322915	4.69847403615869	11.77743838967587
C	3.35634822733815	2.94615441776517	12.04553234254836
C	4.60980040727342	3.47752389612957	12.48043100151379
C	7.53011049685641	1.65167621631016	10.07842745175840
C	2.33188245433125	2.07293483172906	7.58079056075161
C	3.99569999808547	3.71302673182109	7.57343363259984
C	5.64182529031913	5.37103489376395	7.55219081106306
C	1.75750032464175	0.92062111233382	8.12796408551571
C	1.73685488883846	2.66162837088673	6.43252886667650
C	3.36517781893551	4.33564425689641	6.43536939807428
C	6.80045220776892	5.94441413466349	8.08646697298423
H	7.27111343035954	5.45928275930318	8.93325624669740
C	7.34097198197134	7.09694312275830	7.53669222515564
C	5.81553401113436	0.74532759044284	6.87233358915353
C	8.12961896614882	2.66899039664560	7.75749450313805
C	3.47348265980514	6.13837062210531	10.08516128562538
C	4.84353331178627	-0.90301653211793	9.44756948820334
H	2.21735881258592	0.48203860266811	9.00553469803210
C	0.62332044103663	0.35546001132290	7.56475355954492
C	1.45508047603662	3.74945733249123	10.45769238687613
H	8.24523092939349	7.52087970600125	7.95659840354356
C	6.72240297128014	7.70733991823376	6.44293962076828
H	0.18889787457511	-0.53336119109197	8.00636966679126
C	6.61240152933392	0.01488729397151	11.89939711531813
C	5.99809275510385	5.62192756036294	11.97869697338081
C	2.68959259722023	1.70777464585747	12.56998612568512
C	5.44488882568551	2.96513255498120	13.61395322959298
H	7.03752420831254	0.67170453635262	12.65910278913128
H	7.20756568965312	-0.90685767511036	11.89740424420832
H	5.60403474020613	-0.26197026286890	12.21978164895194
H	4.80973689163093	0.32419798676424	6.83343587053101
H	6.47646995263099	0.08089692432526	6.30371865639765
H	5.79328350718859	1.70158504469998	6.34361642381262
C	8.58105507646981	2.33588935145893	10.90309567531450
H	1.44482964322811	4.20082645944675	9.46374824503670
H	0.69826690263515	4.26975745953600	11.05657850125413
H	1.12668724901094	2.71444067473501	10.34714047497351
H	7.50066816315687	3.15288716938596	7.00716334627423

H	8.88032590161574	2.07721160586828	7.22076290778639
H	8.66395367050339	3.45441675878377	8.29472108698785
H	4.40326527637859	6.64289292267318	9.81869925142196
H	2.83968646393708	6.86568009708111	10.60543582258838
H	2.96330070564197	5.88025537575779	9.15392227818574
H	8.78582512963969	3.34708224798969	10.54075361730423
H	9.53124876514260	1.78978774084633	10.87844966972155
H	8.28406908002884	2.41543478116968	11.95166385482165
H	5.28580859840345	1.90002932165678	13.78830859595229
H	5.19367899205865	3.48666225766585	14.54575753994633
H	6.51331941463146	3.12205141407814	13.44278832636011
H	4.19928251439086	-0.85705086125748	10.33201344187542
H	5.33158520534151	-1.88470190137956	9.46605152843658
H	4.20208145068574	-0.87679070407361	8.56525496237803
H	2.06392139622720	1.23394489680937	11.80829615990556
H	2.03656983725185	1.93152122802852	13.42176418528284
H	3.41707872349115	0.96664535092541	12.91137894975589
H	6.93054677657614	5.07500045819130	12.14835722328675
H	5.84904087933960	6.27178776956239	12.84916070425784
H	6.14583061708105	6.27186342705689	11.11369872639634
C	5.00994145721975	5.99593869284187	6.44394706627108
N	2.29271859014637	3.78374247008204	5.86378782874436
C	0.60164870033974	2.06914337260501	5.87001797004602
Gd	2.24505815418782	5.45954990294525	4.11846386828422
N	3.84920664125622	5.47231468423075	5.92206552003993
C	0.04473630481854	0.92978323904245	6.43053289056897
C	5.56449249803426	7.16299473518550	5.90915988173859
H	7.14363324359745	8.60707773489816	6.01064632459878
H	0.16330846929889	2.52579145061499	4.99042524061297
C	0.41014931242108	6.60790974618495	5.73869918634478
C	-0.29896019508544	6.15054161169654	4.59711809988366
C	3.49460364620616	3.29315943219008	2.92799109827836
C	1.25896920076817	7.67249341178491	5.33180568119936
C	4.55626176894808	4.22475551573868	3.07629101074801
C	1.08048061050799	7.87261679984815	3.93717723957969
C	2.54767563580135	3.84284728210882	2.02956615902270
C	4.27595195019590	5.35417050255494	2.26620873672336
C	3.04228577801847	5.12412642308799	1.60294867331506
C	0.10785085629552	6.93431507834720	3.48145629775990
H	-0.83936379931080	0.48811365721024	5.98652561672006
H	5.06979897617741	7.62818852967623	5.06458144398356
C	0.25557494244108	6.10766486152652	7.14310731122311
C	-1.36504142695393	5.09489140889932	4.56715313977626
C	3.45741031369559	1.91861562101544	3.52328415286588
C	2.12258563673223	8.49685881067975	6.23950894704086
C	5.80350080440650	3.98567609569711	3.86959327887256
C	1.72548542262815	8.94737795756151	3.11128492373763
C	1.33328163744373	3.14740352955424	1.48914735671680
C	5.16839794530954	6.54100548227705	2.04859114256980
C	2.49361733646612	5.94459780945780	0.47667545303527
C	-0.52029524927873	6.88631401073149	2.12114540149221

H	1.19943719165226	6.16471521173827	7.68998354230135
H	-0.47492309392242	6.70462433821754	7.70120684804501
H	-0.07933807525255	5.06909984270460	7.16985151773706
H	-1.31472285609323	4.44989897620424	5.44582563474707
H	-2.36586097464387	5.54229691495134	4.54848056070745
H	-1.29107616260880	4.45787818924124	3.67919136861378
H	2.43708462183061	1.55161114429960	3.64549334866679
H	3.98828573688761	1.20328284240116	2.88395594747058
H	3.93313704274672	1.88951687231098	4.50505289709020
H	2.93083230793626	8.98712582409670	5.69275348217949
H	1.53885812839399	9.28717353806795	6.72583971192260
H	2.57806679489673	7.89498128062899	7.02849530717874
H	5.59154925863847	3.45591961680984	4.80148528841564
H	6.51434192926518	3.36998569083339	3.30650822203249
H	6.30767580513624	4.91728253642783	4.12923657038231
H	1.75466776211326	8.67930680807592	2.05281145715382
H	1.17985140683400	9.89503545766296	3.18811588989515
H	2.75281121141308	9.14710397429239	3.42967499955343
H	0.54440884505164	3.85239297969036	1.21473930784221
H	1.57348858402425	2.57102829919704	0.58740301626559
H	0.91610665176362	2.44336999631379	2.21367345747564
H	5.86742229634555	6.67288644656721	2.87639787544333
H	5.76483242219456	6.42836885840573	1.13545145585479
H	4.59796088126885	7.46854366302901	1.94076679400510
H	2.63875932390463	7.01581661616041	0.63639986618248
H	3.00288306544311	5.69196360655252	-0.46198812196157
H	1.42895658645455	5.76716453878206	0.32231870513570
H	-0.69477030227316	5.86138859861373	1.78030140488561
H	-1.49623507337887	7.38658184860934	2.12627921331170
H	0.09460934155175	7.38660004611166	1.37236434632958

Cartesian coordinates of the geometry optimized structure of the $[(Cp^{*}{}_{2}Gd)_{2}(\mu\text{-flv}^{\bullet})]^{-}$ anion in **3-Gd**, at def2-TZVP level using the uTPSSh functional, SARC/J auxiliary basis set and D3BJ dispersion correction. (Final single point energy = –3850.688490127771 Hartrees)

Gd	-5.69806151542765	10.72267322454482	17.72187959552526
N	-4.78948742977963	9.34045387692065	15.87848157463883
N	-3.32853622959107	10.70368743166382	16.99360270531980
C	-7.81457464746039	12.19041837682771	16.76054877036240
C	-5.74534471848979	12.86112838329190	16.01437901273666
C	-6.95951355405585	9.60031974907618	19.87515542718182
C	-6.61766288607314	8.50417851430470	19.03845987304433
C	-5.94895340088464	13.41650365607713	17.30471859414614
C	-7.23039894269250	13.00939112323299	17.76446303315755
C	-5.75273381513794	10.14787227990510	20.39135401394684
C	-4.67117212087047	9.37758840824614	19.88583313292649
C	-5.20498898611218	8.35964206815783	19.05458202559708
C	-6.89680852029723	12.10175582724204	15.68006317133000
Gd	-0.30952708641616	8.06308088680057	13.85620876975240
N	-2.61071753495946	7.92565203478585	14.75813470242101
N	-1.15365963491578	9.35542880558268	15.79421593571762
C	0.47049529253968	6.35723691281264	15.84272145793289
C	0.35923570985660	8.90548068907206	11.34439949833686
C	-1.65982925438787	9.71312851827992	12.09292410344564
C	-0.04128470893278	5.51512676838412	14.82110902977420
C	1.91436622516474	6.44454549860496	14.05423161754383
C	0.84846272088360	5.56794741803005	13.71499630958605
C	-0.63356976686459	10.52071008673076	12.64575005190131
C	1.67560850563026	6.93543146634397	15.36605280633278
C	-1.04740912879072	8.70671086942340	11.29971207558682
C	-2.41208331599127	8.96841082993383	15.57012297961954
C	-3.90919178591493	7.55857052856024	14.47336378625219
C	-3.52859994234190	9.68792055752266	16.15044020733493
C	-5.00485198233620	8.26283086727105	15.04577856076846
C	-0.93881925823503	10.42797899466604	16.63257103189941
C	-2.03265960084031	11.09523772299704	17.25063079290583
C	-0.14428361333477	6.54778605265862	17.19737744689763
C	-6.30668227163164	7.85494995186903	14.74119739997851
C	-9.19374423860517	11.59873899326153	16.78512187000090
C	-1.77543788156281	12.16685729652971	18.11064402816154
C	0.36053282345969	10.87680685222013	16.88612925546299
H	-7.12949053583363	8.40218546194130	15.18699866009687
C	-6.54238581193380	6.78669498294793	13.88353842803470
H	-9.21428346162781	10.58746431443762	16.36545871904477
H	-9.90353724106986	12.19864236655330	16.20107694945834
H	-9.58461219450597	11.53778040565691	17.80316079179030
C	1.36720687507104	8.17374419941306	10.50975812900979
C	0.61558447749031	10.01920136260767	12.19036056050940
H	1.18184675535419	10.36411044507624	16.39851173440972
C	0.59551215049838	11.94925600318525	17.73867819032695
H	0.13543520370835	7.50703185265073	17.63677587250357

H	0.16518959063924	5.75816857563854	17.89421915472741
H	-1.23533461642362	6.52270956424768	17.14288702462789
C	-3.13230094670353	9.95497364032782	12.23734016011907
C	-4.16661541267247	6.49246039409297	13.60674249925917
C	-4.56160587046357	13.07896517061091	15.12031102630437
C	-8.35495109294287	9.99290261937631	20.25917768352062
C	-1.25897054568722	4.64740089175612	14.93728150609928
C	3.15720801480934	6.70305902200252	13.25594884225912
C	0.72246629288331	4.75858302714832	12.45740369756085
C	-7.58994066592391	7.60447704565949	18.33306511732536
H	-2.62015071478496	12.65928980257320	18.57906591924607
C	-0.47568757217659	12.59332074673163	18.35674114860872
C	-5.02636692635953	14.35471803892519	18.02583368198690
H	-7.56108652957220	6.48975001360038	13.66042566761305
C	-5.46880575977705	6.10685051076846	13.30992076562249
C	-7.88980708174483	13.49954467426192	19.01874509853346
C	-0.84061101453980	11.75409911054701	13.47309922011970
C	-5.63064204770848	11.27299359202220	21.37692481723288
H	-8.71277964435915	12.85060372844573	19.32455920905833
H	-8.30652983037144	14.50641276413221	18.88242259011072
H	-7.18695410349567	13.55897887181323	19.85495826380164
H	-3.31996868783560	5.97283824436278	13.17268736565704
C	-3.22608212216231	9.55102292212051	20.24671173732725
C	-4.41636591599840	7.27391765449217	18.38546027263153
H	-0.30040871204594	13.42750098350140	19.02697852738584
C	2.60280581221100	7.83064766490059	16.13382812382184
H	-0.31333097409959	4.70535525335396	12.10662718754836
H	1.06170989634941	3.72499881778311	12.60382601399844
H	1.31955733888636	5.18106163851022	11.64615240690733
C	-1.76207029028688	7.67247383089371	10.47932003222284
H	2.37613167075425	8.27550815650442	10.91349291780316
H	1.38901429325803	8.56465199914927	9.48377783841614
H	1.14595608298771	7.10517956162720	10.43680717552626
H	-5.64401420921117	5.27655466787443	12.63488116569813
H	-2.07080382201489	5.15534474515132	15.46258684764234
H	-1.03926223834541	3.72254692584672	15.48654299627722
H	-1.63691222920920	4.35281687270051	13.95499107238485
H	-4.78027593305317	11.92452644030505	21.14912064954405
H	-5.48003416308562	10.90257124854671	22.39932046874467
H	-6.52797210121084	11.89574144246242	21.38847358346658
C	-7.13691282919611	11.41279012720461	14.36963666112315
H	-4.96045109711890	14.12850383842685	19.09587600228783
H	-5.36549442868163	15.39580935677557	17.94408729296910
H	-4.01596440747605	14.31083485315887	17.61512532451961
H	-2.56570622434610	9.18909445911402	19.45658699303945
H	-2.97502395040316	8.99910358020017	21.16201813504471
H	-2.97130621831532	10.59901640214080	20.42223180863609
H	-8.40136703430013	11.02059179192588	20.62467139117157
H	-8.74295160850483	9.34811436252862	21.05890657035059
H	-9.05024264708383	9.90823889284413	19.41927824256989
H	-3.71171444750490	9.03921666793700	12.10896789195317

H	-3.49019275161128	10.67835011895398	11.49369596627787
H	-3.38068664156269	10.36036220923851	13.22086754618927
C	1.95592747674702	10.62633667643734	12.48690295155398
H	2.98150435952637	8.65593675587682	15.52045245042936
H	3.48051233014836	7.28159388873694	16.49961856756475
H	2.10634489760904	8.26501587691332	17.00331608934179
H	-1.76624217053414	11.70331206943353	14.04945066613942
H	-0.90212452694550	12.64988501926409	12.84146639800190
H	-0.02822482293194	11.91334673363622	14.18528758757824
H	1.61208093805284	12.27989063567770	17.92053765663935
H	-8.46320699692845	8.15589305186047	17.96935490868478
H	-7.96666554713824	6.81419612784446	18.99599041478592
H	-7.12836762483560	7.11421569822814	17.47343331583208
H	3.03626677349475	6.40313635715526	12.21377149519462
H	4.01157801859097	6.14295520251079	13.65834170570634
H	3.44188764386095	7.76029492490673	13.26391329745208
H	-3.65248930112362	13.27982291389168	15.68999501728918
H	-4.72231582163511	13.92552481540423	14.44034582008730
H	-4.36861721654721	12.20063157308171	14.50032740399013
H	-4.90621913303007	6.91323540551067	17.47894235611666
H	-4.28096760260554	6.41172197147270	19.05070135023041
H	-3.41834425928512	7.61760913389313	18.10377309349455
H	-6.23653429390844	10.92021525600799	13.99641673008813
H	-7.46641118226483	12.12354825483656	13.60060338283011
H	-7.91442265959908	10.64999665213556	14.45440708396992
H	-1.17748071576130	6.75163911518502	10.39067770305629
H	-1.95723509641337	8.02589383079455	9.45797285525019
H	-2.72820461703892	7.41105162882123	10.91834588848231
H	2.00038119636113	11.02518238405116	13.50511177466588
H	2.18620833960426	11.45906569614830	11.80934900559818
H	2.76291896037405	9.89603189875535	12.38335104434618

9 Python Scripts for Magnetic Hysteresis Analyses

Python script used for fitting experimental hysteresis data and generating the first derivative.[1]

```
import pandas as pd
import numpy as np
from scipy.optimize import curve_fit
import matplotlib.pyplot as plt
import os

# Step 1: Define the file path and load the CSV file
file_path = "FilePath/FileName.csv"
try:
    data = pd.read_csv(file_path)
except FileNotFoundError:
    print(f"Error: The file {file_path} was not found.")
    exit()

# Step 2: Extract the magnetic field and moment per gram columns
H = data['Field'].values  # Magnetic field in Tesla
M_g = data['Moment_emu_g'].values  # Magnetic moment per gram in emu/g

# Step 3: Check for invalid data
if not (np.all(np.isfinite(H)) and np.all(np.isfinite(M_g))):
    print("Error: Data contains NaN or infinite values.")
    exit()

# Step 4: Split into descending and ascending branches
k = H.argmin()  # Index of minimum field
H_desc = H[:k+1]  # Descending branch
M_g_desc = M_g[:k+1]
H_asc = H[k:]     # Ascending branch
M_g_asc = M_g[k:]

# Step 5: Define the scaled arctangent model with four components and baseline
def arctan_model(H, Ms1, a1, Hc1, Ms2, a2, Hc2, Ms3, a3, Hc3, Ms4, a4, Hc4, baseline):
    """
    Scaled Model: M(H) = Ms1 * (2/pi) * arctan(a1 * (H - Hc1)) + Ms2 * (2/pi) * arctan(a2 * (H - Hc2))
+ Ms3 * (2/pi) * arctan(a3 * (H - Hc3)) + Ms4 * (2/pi) * arctan(a4 * (H - Hc4)) + baseline
    Parameters:
        Ms1, Ms2, Ms3, Ms4: Saturation magnetizations per gram (emu/g)
        a1, a2, a3, a4: Steepness factors
        Hc1, Hc2, Hc3, Hc4: Coercive fields (Tesla)
        baseline: Constant offset (emu/g)
    """
    return (Ms1 * (2 / np.pi) * np.arctan(a1 * (H - Hc1)) +
            Ms2 * (2 / np.pi) * np.arctan(a2 * (H - Hc2)) +
            Ms3 * (2 / np.pi) * np.arctan(a3 * (H - Hc3)) +
            Ms4 * (2 / np.pi) * np.arctan(a4 * (H - Hc4)) +
            baseline)

# Step 6: Set initial guesses and bounds
```

```
p0 = [15, 15, 0.05, 15, 15, -0.5, 4, 50, 0.03, 2, 60, 0.01, 0.0]  # [Ms1, a1, Hc1, Ms2, a2, Hc2, Ms3,
a3, Hc3, Ms4, a4, Hc4, baseline]
bounds = (
    [0, 1, -2.5, 0, 1, -2.5, 0, 1, -0.7, 0, 1, -0.5, -10],  # Lower bounds
    [50, 50, 2.5, 50, 50, 2.5, 50, 50, 0.7, 50, 100, 0.5, 10]  # Upper bounds
)

# Step 7: Fit the descending branch
try:
    popt_desc, _ = curve_fit(
        arctan_model, H_desc, M_g_desc, p0=p0, bounds=bounds, maxfev=40000
    )
    print("Descending branch fit parameters [Ms1, a1, Hc1, Ms2, a2, Hc2, Ms3, a3, Hc3, Ms4, a4,
Hc4, baseline]:", popt_desc)
except RuntimeError as e:
    print("Error fitting descending branch:", e)

# Step 8: Fit the ascending branch
p0_asc = [15, 15, -0.05, 15, 15, 0.5, 4, 50, -0.03, 2, 60, -0.01, 0.0]
try:
    popt_asc, _ = curve_fit(
        arctan_model, H_asc, M_g_asc, p0=p0_asc, bounds=bounds, maxfev=40000
    )
    print("Ascending branch fit parameters [Ms1, a1, Hc1, Ms2, a2, Hc2, Ms3, a3, Hc3, Ms4, a4,
Hc4, baseline]:", popt_asc)
except RuntimeError as e:
    print("Error fitting ascending branch:", e)

# Step 9: Generate fitted curves
H_fit_desc = np.linspace(min(H_desc), max(H_desc), 500)
M_g_fit_desc = arctan_model(H_fit_desc, *popt_desc)
H_fit_asc = np.linspace(min(H_asc), max(H_asc), 500)
M_g_fit_asc = arctan_model(H_fit_asc, *popt_asc)

# Step 10: Compute goodness-of-fit metrics
def goodness_of_fit(y_true, y_pred):
    mask = np.isfinite(y_true) & np.isfinite(y_pred)
    y_true = y_true[mask]
    y_pred = y_pred[mask]

    ss_tot = np.sum((y_true - np.mean(y_true))**2)
    ss_res = np.sum((y_true - y_pred)**2)
    r_squared = 1 - ss_res / ss_tot if ss_tot != 0 else np.nan

    rmse = np.sqrt(np.mean((y_true - y_pred)**2))

    chi_squared = np.sum((y_true - y_pred)**2)
    dof = len(y_true) - len(popt_desc)
    reduced_chi_squared = chi_squared / dof if dof > 0 else np.nan

    return r_squared, rmse, chi_squared, reduced_chi_squared
```

```
# Interpolate the fit to match raw data points
interp_desc = np.interp(H_desc, H_fit_desc, M_g_fit_desc)
interp_asc = np.interp(H_asc, H_fit_asc, M_g_fit_asc)

# Compute metrics
r2_desc, rmse_desc, chi2_desc, reduced_chi2_desc = goodness_of_fit(M_g_desc, interp_desc)
r2_asc, rmse_asc, chi2_asc, reduced_chi2_asc = goodness_of_fit(M_g_asc, interp_asc)

# Step 11: Export fit lines to CSV
output_dir = os.path.dirname(file_path)
output_file = os.path.join(output_dir, "HysFit_SArcTan_FitLines.csv")
fit_data = {
    'Field_T': np.concatenate([H_fit_desc, H_fit_asc]),
    'Moment_emu_g_Descending_Fit': np.concatenate([M_g_fit_desc, np.full_like(M_g_fit_asc,
np.nan)]),
    'Moment_emu_g_Ascending_Fit': np.concatenate([np.full_like(M_g_fit_desc, np.nan),
M_g_fit_asc])
}
fit_df = pd.DataFrame(fit_data)
fit_df.to_csv(output_file, index=False)
print(f"Fit lines exported to {output_file}")

# Step 12: Calculate the first derivatives of the fit lines
def arctan_derivative(H, Ms1, a1, Hc1, Ms2, a2, Hc2, Ms3, a3, Hc3, Ms4, a4, Hc4, baseline):
    """
    First derivative of the scaled arctangent model with respect to H.
    dM/dH = (2/pi) * [ Ms1 * a1 / (1 + (a1*(H-Hc1))^2) + Ms2 * a2 / (1 + (a2*(H-Hc2))^2) + Ms3 * a3
/ (1 + (a3*(H-Hc3))^2) + Ms4 * a4 / (1 + (a4*(H-Hc4))^2) ]
    (Baseline does not contribute to the derivative)
    """
    term1 = Ms1 * (2 / np.pi) * a1 / (1 + (a1 * (H - Hc1))**2)
    term2 = Ms2 * (2 / np.pi) * a2 / (1 + (a2 * (H - Hc2))**2)
    term3 = Ms3 * (2 / np.pi) * a3 / (1 + (a3 * (H - Hc3))**2)
    term4 = Ms4 * (2 / np.pi) * a4 / (1 + (a4 * (H - Hc4))**2)
    return term1 + term2 + term3 + term4

# Compute derivatives
dM_dH_desc = arctan_derivative(H_fit_desc, *popt_desc)
dM_dH_asc = arctan_derivative(H_fit_asc, *popt_asc)

# Step 13: Export derivatives to CSV
output_deriv_file = os.path.join(output_dir, "HysFit_SArcTan_FitLinesDeriv.csv")
deriv_data = {
    'Field_T': np.concatenate([H_fit_desc, H_fit_asc]),
    'dM_dH_Descending_Fit': np.concatenate([dM_dH_desc, np.full_like(dM_dH_asc, np.nan)]),
    'dM_dH_Ascending_Fit': np.concatenate([np.full_like(dM_dH_desc, np.nan), dM_dH_asc])
}
deriv_df = pd.DataFrame(deriv_data)
deriv_df.to_csv(output_deriv_file, index=False)
print(f"Derivatives exported to {output_deriv_file}")
```

```
# Step 14: Export parameters with goodness-of-fit metrics
output_params_desc_file = os.path.join(output_dir,
"HysFit_SArcTan_Parameters_Descending_XK.txt")
with open(output_params_desc_file, 'w') as f:
    f.write("Scaled Arctangent Fit Parameters for Descending Branch (X K):\n")
    f.write("==============================================\n\n")
    f.write(f"  Ms1 (Saturation Mag 1) = {popt_desc[0]:.4f} emu/g\n")
    f.write(f"  a1 (Steepness 1)       = {popt_desc[1]:.4f} T^-1\n")
    f.write(f"  Hc1 (Coercive Field 1) = {popt_desc[2]:.4f} T\n")
    f.write(f"  Ms2 (Saturation Mag 2) = {popt_desc[3]:.4f} emu/g\n")
    f.write(f"  a2 (Steepness 2)       = {popt_desc[4]:.4f} T^-1\n")
    f.write(f"  Hc2 (Coercive Field 2) = {popt_desc[5]:.4f} T\n")
    f.write(f"  Ms3 (Saturation Mag 3) = {popt_desc[6]:.4f} emu/g\n")
    f.write(f"  a3 (Steepness 3)       = {popt_desc[7]:.4f} T^-1\n")
    f.write(f"  Hc3 (Coercive Field 3) = {popt_desc[8]:.4f} T\n")
    f.write(f"  Ms4 (Saturation Mag 4) = {popt_desc[9]:.4f} emu/g\n")
    f.write(f"  a4 (Steepness 4)       = {popt_desc[10]:.4f} T^-1\n")
    f.write(f"  Hc4 (Coercive Field 4) = {popt_desc[11]:.4f} T\n")
    f.write(f"  Baseline               = {popt_desc[12]:.4f} emu/g\n")
    f.write("--------------------------------\n\n")
    f.write("Goodness-of-Fit Metrics (vs. Raw Data):\n")
    f.write("==============================================\n")
    f.write(f"  R-squared            = {r2_desc:.4f}\n")
    f.write(f"  RMSE                 = {rmse_desc:.4f} emu/g\n")
    f.write(f"  Chi-squared          = {chi2_desc:.4f}\n")
    f.write(f"  Reduced Chi-squared  = {reduced_chi2_desc:.4f}\n")
print(f"Descending parameters exported to {output_params_desc_file}")

output_params_asc_file = os.path.join(output_dir,
"HysFit_SArcTan_Parameters_Ascending_XK.txt")
with open(output_params_asc_file, 'w') as f:
    f.write("Scaled Arctangent Fit Parameters for Ascending Branch (X K):\n")
    f.write("==============================================\n\n")
    f.write(f"  Ms1 (Saturation Mag 1) = {popt_asc[0]:.4f} emu/g\n")
    f.write(f"  a1 (Steepness 1)       = {popt_asc[1]:.4f} T^-1\n")
    f.write(f"  Hc1 (Coercive Field 1) = {popt_asc[2]:.4f} T\n")
    f.write(f"  Ms2 (Saturation Mag 2) = {popt_asc[3]:.4f} emu/g\n")
    f.write(f"  a2 (Steepness 2)       = {popt_asc[4]:.4f} T^-1\n")
    f.write(f"  Hc2 (Coercive Field 2) = {popt_asc[5]:.4f} T\n")
    f.write(f"  Ms3 (Saturation Mag 3) = {popt_asc[6]:.4f} emu/g\n")
    f.write(f"  a3 (Steepness 3)       = {popt_asc[7]:.4f} T^-1\n")
    f.write(f"  Hc3 (Coercive Field 3) = {popt_asc[8]:.4f} T\n")
    f.write(f"  Ms4 (Saturation Mag 4) = {popt_asc[9]:.4f} emu/g\n")
    f.write(f"  a4 (Steepness 4)       = {popt_asc[10]:.4f} T^-1\n")
    f.write(f"  Hc4 (Coercive Field 4) = {popt_asc[11]:.4f} T\n")
    f.write(f"  Baseline               = {popt_asc[12]:.4f} emu/g\n")
    f.write("--------------------------------\n\n")
    f.write("Goodness-of-Fit Metrics (vs. Raw Data):\n")
    f.write("==============================================\n")
    f.write(f"  R-squared            = {r2_asc:.4f}\n")
```

```
    f.write(f"  RMSE                 = {rmse_asc:.4f} emu/g\n")
    f.write(f"  Chi-squared          = {chi2_asc:.4f}\n")
    f.write(f"  Reduced Chi-squared  = {reduced_chi2_asc:.4f}\n")
print(f"Ascending parameters exported to {output_params_asc_file}")

# Step 15: Plot the original hysteresis loop
plt.figure(figsize=(10, 6))
plt.plot(H_desc, M_g_desc, 'c.', label='Descending Data')
plt.plot(H_fit_desc, M_g_fit_desc, 'darkgreen', label='Descending Fit')
plt.plot(H_asc, M_g_asc, 'm.', label='Ascending Data')
plt.plot(H_fit_asc, M_g_fit_asc, 'purple', label='Ascending Fit')
plt.xlabel('Magnetic Field (T)')
plt.ylabel('Magnetic Moment per Gram (emu/g)')
plt.title('Hysteresis Loop with Scaled Arctangent Fits (X K)')
plt.legend()
plt.grid(True)
plt.show()

# Step 16: Plot the derivatives
plt.figure(figsize=(10, 6))
plt.plot(H_fit_desc, dM_dH_desc, 'darkgreen', label='Descending Fit Derivative')
plt.plot(H_fit_asc, dM_dH_asc, 'purple', label='Ascending Fit Derivative')
plt.xlabel('Magnetic Field (T)')
plt.ylabel('dM/dH (emu/g/T)')
plt.title('First Derivative of Hysteresis Loop Fits (X K)')
plt.legend()
plt.grid(True)
plt.show()
```

Python script used for Cauchy probability distribution function analyses of the first derivatives of magnetic hysteresis fits.[1]

```
import pandas as pd
import numpy as np
from scipy.optimize import curve_fit
import matplotlib.pyplot as plt
import os

# Step 1: Define the file path for the derivative data
input_file = "FilePathFileName.csv"
try:
    deriv_data = pd.read_csv(input_file)
except FileNotFoundError:
    print(f"Error: The file {input_file} was not found.")
    exit()

# Step 2: Extract descending and ascending branch derivatives
H_desc = deriv_data['Field_T'][~pd.isna(deriv_data['dM_dH_Descending_Fit'])].values
dM_dH_desc =
deriv_data['dM_dH_Descending_Fit'][~pd.isna(deriv_data['dM_dH_Descending_Fit'])].values
```

```
H_asc = deriv_data['Field_T'][~pd.isna(deriv_data['dM_dH_Ascending_Fit'])].values
dM_dH_asc                                                                          =
deriv_data['dM_dH_Ascending_Fit'][~pd.isna(deriv_data['dM_dH_Ascending_Fit'])].values

# Step 3: Define the Cauchy PDF model with variable number of components
def cauchy_model(H, *params):
    """
    Sum of N Cauchy PDFs to fit dM/dH.
    Parameters: [P1, gamma1, HP1, P2, gamma2, HP2, ...] for N components.
    """
    n_components = len(params) // 3
    result = np.zeros_like(H, dtype=float)
    for i in range(n_components):
        P = params[3 * i]
        gamma = params[3 * i + 1]
        HP = params[3 * i + 2]
        result += P / (np.pi * gamma * (1 + ((H - HP) / gamma)**2))
    return result

# Step 4: Determine number of components based on observed peaks
from scipy.signal import find_peaks
peaks_desc, properties_desc = find_peaks(dM_dH_desc, height=0.03)
peaks_asc, properties_asc = find_peaks(dM_dH_asc, height=0.03)
n_components_desc = max(len(peaks_desc), 1)
n_components_asc = max(len(peaks_asc), 1)
if n_components_desc < 1:
    n_components_desc = 2
if n_components_asc < 1:
    n_components_asc = 2
print(f"Detected {n_components_desc} components for descending branch.")
print(f"Detected {n_components_asc} components for ascending branch.")

# Step 5: Set initial guesses and bounds for descending branch using peak positions
initial_guesses_desc = []
bounds_lower_desc = []
bounds_upper_desc = []
peak_heights_desc   =   properties_desc['peak_heights']   if   len(peaks_desc)   >   0   else
[max(dM_dH_desc)]
peak_positions_desc = H_desc[peaks_desc] if len(peaks_desc) > 0 else [0.0]
for i in range(n_components_desc):
    P_guess = peak_heights_desc[min(i, len(peak_heights_desc)-1)] * 3
    HP_guess = peak_positions_desc[min(i, len(peak_positions_desc)-1)]
    gamma_guess = 0.1 if abs(HP_guess) < 0.1 else 0.3
    initial_guesses_desc.extend([P_guess, gamma_guess, HP_guess])
    bounds_lower_desc.extend([0, 0.01, -7])
    bounds_upper_desc.extend([P_guess * 5, 1, 7])
p0_desc = np.array(initial_guesses_desc)
bounds_desc = (np.array(bounds_lower_desc), np.array(bounds_upper_desc))

# Step 6: Set initial guesses and bounds for ascending branch using peak positions
initial_guesses_asc = []
```

```
bounds_lower_asc = []
bounds_upper_asc = []
peak_heights_asc = properties_asc['peak_heights'] if len(peaks_asc) > 0 else [max(dM_dH_asc)]
peak_positions_asc = H_asc[peaks_asc] if len(peaks_asc) > 0 else [0.0]
for i in range(n_components_asc):
    P_guess = peak_heights_asc[min(i, len(peak_heights_asc)-1)] * 3
    HP_guess = peak_positions_asc[min(i, len(peak_positions_asc)-1)]
    gamma_guess = 0.1 if abs(HP_guess) < 0.1 else 0.3
    initial_guesses_asc.extend([P_guess, gamma_guess, HP_guess])
    bounds_lower_asc.extend([0, 0.01, -7])
    bounds_upper_asc.extend([P_guess * 5, 1, 7])
p0_asc = np.array(initial_guesses_asc)
bounds_asc = (np.array(bounds_lower_asc), np.array(bounds_upper_asc))

# Step 7: Fit the derivatives with the Cauchy model
try:
    popt_cauchy_desc, _ = curve_fit(
        cauchy_model, H_desc, dM_dH_desc, p0=p0_desc, bounds=bounds_desc, maxfev=60000
    )
    print("Cauchy fit parameters for descending branch:", popt_cauchy_desc)
except RuntimeError as e:
    print("Error fitting Cauchy model to descending branch:", e)

try:
    popt_cauchy_asc, _ = curve_fit(
        cauchy_model, H_asc, dM_dH_asc, p0=p0_asc, bounds=bounds_asc, maxfev=60000
    )
    print("Cauchy fit parameters for ascending branch:", popt_cauchy_asc)
except RuntimeError as e:
    print("Error fitting Cauchy model to ascending branch:", e)

# Step 8: Calculate individual Cauchy components and sort by HP with reversal for descending
def compute_components(H, params, is_descending=False):
    n_components = len(params) // 3
    components = []
    hp_indices = []
    for i in range(n_components):
        P = params[3 * i]
        gamma = params[3 * i + 1]
        HP = params[3 * i + 2]
        component = P / (np.pi * gamma * (1 + ((H - HP) / gamma)**2))
        components.append(component)
        hp_indices.append((i, HP))
    # Sort components by HP
    sorted_indices = [i[0] for i in sorted(hp_indices, key=lambda x: x[1])]
    if is_descending:
        sorted_indices = sorted_indices[::-1]  # Reverse order for descending branch
    sorted_components = [components[i] for i in sorted_indices]
    return sorted_components, sorted_indices

# Descending branch (reverse order)
```

```
components_desc, sorted_indices_desc = compute_components(H_desc, popt_cauchy_desc, is_descending=True)
total_P_desc = sum(popt_cauchy_desc[::3])
percent_contrib_desc = [(popt_cauchy_desc[3 * sorted_indices_desc[i]] / total_P_desc * 100) for i in range(n_components_desc)]
Ms_desc = [(popt_cauchy_desc[3 * sorted_indices_desc[i]] * popt_cauchy_desc[3 * sorted_indices_desc[i] + 1]) / 2 for i in range(n_components_desc)]

# Ascending branch (normal order)
components_asc, sorted_indices_asc = compute_components(H_asc, popt_cauchy_asc, is_descending=False)
total_P_asc = sum(popt_cauchy_asc[::3])
percent_contrib_asc = [(popt_cauchy_asc[3 * sorted_indices_asc[i]] / total_P_asc * 100) for i in range(n_components_asc)]
Ms_asc = [(popt_cauchy_asc[3 * sorted_indices_asc[i]] * popt_cauchy_asc[3 * sorted_indices_asc[i] + 1]) / 2 for i in range(n_components_asc)]

# Step 9: Compute goodness-of-fit metrics
def goodness_of_fit(y_true, y_pred, n_params):
    mask = np.isfinite(y_true) & np.isfinite(y_pred)
    y_true = y_true[mask]
    y_pred = y_pred[mask]

    ss_tot = np.sum((y_true - np.mean(y_true))**2)
    ss_res = np.sum((y_true - y_pred)**2)
    r_squared = 1 - ss_res / ss_tot if ss_tot != 0 else np.nan

    rmse = np.sqrt(np.mean((y_true - y_pred)**2))

    chi_squared = np.sum((y_true - y_pred)**2)
    dof = len(y_true) - n_params
    reduced_chi_squared = chi_squared / dof if dof > 0 else np.nan

    return r_squared, rmse, chi_squared, reduced_chi_squared

# Compute metrics for descending branch
cauchy_fit_desc = cauchy_model(H_desc, *popt_cauchy_desc)
r2_desc, rmse_desc, chi2_desc, reduced_chi2_desc = goodness_of_fit(dM_dH_desc, cauchy_fit_desc, len(popt_cauchy_desc))

# Compute metrics for ascending branch
cauchy_fit_asc = cauchy_model(H_asc, *popt_cauchy_asc)
r2_asc, rmse_asc, chi2_asc, reduced_chi2_asc = goodness_of_fit(dM_dH_asc, cauchy_fit_asc, len(popt_cauchy_asc))

# Step 10: Export fits and parameters
output_dir = os.path.dirname(input_file)

# Export descending fit to CSV
output_fit_desc_file = os.path.join(output_dir, "HysFit_SArcTan_CauchyFit_Descending_XK.csv")
```

```
fit_desc_data = {
    'Field_T': H_desc,
    'dM_dH_Descending_Fit': dM_dH_desc,
    'Cauchy_Fit': cauchy_model(H_desc, *popt_cauchy_desc)
}
# Add individual components to the dictionary
for i, comp in enumerate(components_desc):
    fit_desc_data[f'Descending_P_{i+1}'] = comp
fit_desc_df = pd.DataFrame(fit_desc_data)
fit_desc_df.to_csv(output_fit_desc_file, index=False)
print(f"Descending fit exported to {output_fit_desc_file}")

# Export ascending fit to CSV
output_fit_asc_file = os.path.join(output_dir, "HysFit_SArcTan_CauchyFit_Ascending_XK.csv")
fit_asc_data = {
    'Field_T': H_asc,
    'dM_dH_Ascending_Fit': dM_dH_asc,
    'Cauchy_Fit': cauchy_model(H_asc, *popt_cauchy_asc)
}
# Add individual components to the dictionary
for i, comp in enumerate(components_asc):
    fit_asc_data[f'Ascending_P_{i+1}'] = comp
fit_asc_df = pd.DataFrame(fit_asc_data)
fit_asc_df.to_csv(output_fit_asc_file, index=False)
print(f"Ascending fit exported to {output_fit_asc_file}")

# Export total Cauchy fit for descending branch
output_total_desc_file = os.path.join(output_dir,
"HysFit_SArcTan_TotalCauchyFit_Descending_XK.csv")
total_desc_data = {
    'Field_T': H_desc,
    'Total_Cauchy_Fit': cauchy_model(H_desc, *popt_cauchy_desc)
}
total_desc_df = pd.DataFrame(total_desc_data)
total_desc_df.to_csv(output_total_desc_file, index=False)
print(f"Total Cauchy fit for descending branch exported to {output_total_desc_file}")

# Export total Cauchy fit for ascending branch
output_total_asc_file = os.path.join(output_dir,
"HysFit_SArcTan_TotalCauchyFit_Ascending_XK.csv")
total_asc_data = {
    'Field_T': H_asc,
    'Total_Cauchy_Fit': cauchy_model(H_asc, *popt_cauchy_asc)
}
total_asc_df = pd.DataFrame(total_asc_data)
total_asc_df.to_csv(output_total_asc_file, index=False)
print(f"Total Cauchy fit for ascending branch exported to {output_total_asc_file}")

# Export descending parameters to text file with percent contribution, Ms, and goodness-of-fit
output_params_desc_file = os.path.join(output_dir,
"HysFit_SArcTan_CauchyFit_Parameters_Descending_XK.txt")
```

```
with open(output_params_desc_file, 'w') as f:
    f.write("Cauchy Fit Parameters for Descending Branch (X K):\n")
    f.write("==================================================\n\n")
    for i in range(n_components_desc):
        idx = sorted_indices_desc[i]
        P = popt_cauchy_desc[3 * idx]
        gamma = popt_cauchy_desc[3 * idx + 1]
        HP = popt_cauchy_desc[3 * idx + 2]
        perc = percent_contrib_desc[i]
        Ms = Ms_desc[i]
        f.write(f"Component P_{i+1}:\n")
        f.write(f"  Amplitude (P)        = {P:.4f} emu/g/T\n")
        f.write(f"  Half-Width (gamma)    = {gamma:.4f} T\n")
        f.write(f"  Peak Field (HP)       = {HP:.4f} T\n")
        f.write(f"  Percent Contribution  = {perc:.2f}%\n")
        f.write(f"  Saturation Mag (Ms)   = {Ms:.4f} emu/g\n")
        f.write("------------------------------\n\n")
    f.write("Goodness-of-Fit Metrics (vs. Derivative Data):\n")
    f.write("==================================================\n")
    f.write(f"  R-squared            = {r2_desc:.4f}\n")
    f.write(f"  RMSE                 = {rmse_desc:.4f} emu/g/T\n")
    f.write(f"  Chi-squared          = {chi2_desc:.4f}\n")
    f.write(f"  Reduced Chi-squared  = {reduced_chi2_desc:.4f}\n")
print(f"Descending parameters exported to {output_params_desc_file}")

# Export ascending parameters to text file with percent contribution, Ms, and goodness-of-fit
output_params_asc_file = os.path.join(output_dir,
"HysFit_SArcTan_CauchyFit_Parameters_Ascending_XK.txt")
with open(output_params_asc_file, 'w') as f:
    f.write("Cauchy Fit Parameters for Ascending Branch (X K):\n")
    f.write("==================================================\n\n")
    for i in range(n_components_asc):
        idx = sorted_indices_asc[i]
        P = popt_cauchy_asc[3 * idx]
        gamma = popt_cauchy_asc[3 * idx + 1]
        HP = popt_cauchy_asc[3 * idx + 2]
        perc = percent_contrib_asc[i]
        Ms = Ms_asc[i]
        f.write(f"Component P_{i+1}:\n")
        f.write(f"  Amplitude (P)        = {P:.4f} emu/g/T\n")
        f.write(f"  Half-Width (gamma)    = {gamma:.4f} T\n")
        f.write(f"  Peak Field (HP)       = {HP:.4f} T\n")
        f.write(f"  Percent Contribution  = {perc:.2f}%\n")
        f.write(f"  Saturation Mag (Ms)   = {Ms:.4f} emu/g\n")
        f.write("------------------------------\n\n")
    f.write("Goodness-of-Fit Metrics (vs. Derivative Data):\n")
    f.write("==================================================\n")
    f.write(f"  R-squared            = {r2_asc:.4f}\n")
    f.write(f"  RMSE                 = {rmse_asc:.4f} emu/g/T\n")
    f.write(f"  Chi-squared          = {chi2_asc:.4f}\n")
    f.write(f"  Reduced Chi-squared  = {reduced_chi2_asc:.4f}\n")
```

```
print(f"Ascending parameters exported to {output_params_asc_file}")

# Step 11: Plot descending branch with total fit
plt.figure(figsize=(10, 6))
plt.plot(H_desc, dM_dH_desc, 'darkgreen', label='Descending Fit Derivative')
plt.plot(H_desc, cauchy_model(H_desc, *popt_cauchy_desc), 'k-', label='Total Cauchy Fit',
alpha=0.7)
for i, comp in enumerate(components_desc):
    plt.plot(H_desc, comp, ['g--', 'y--', 'b--'][i % 3], label=f'Descending P_{i+1}')
plt.xlabel('Magnetic Field (T)')
plt.ylabel('dM/dH (emu/g/T)')
plt.title('Descending Branch Derivative with Cauchy Components (X K)')
plt.legend()
plt.grid(True)
plt.show()

# Step 12: Plot ascending branch with total fit
plt.figure(figsize=(10, 6))
plt.plot(H_asc, dM_dH_asc, 'purple', label='Ascending Fit Derivative')
plt.plot(H_asc, cauchy_model(H_asc, *popt_cauchy_asc), 'k-', label='Total Cauchy Fit',
alpha=0.7)
for i, comp in enumerate(components_asc):
    plt.plot(H_asc, comp, ['g-.', 'y-.', 'b-.'][i % 3], label=f'Ascending P_{i+1}')
plt.xlabel('Magnetic Field (T)')
plt.ylabel('dM/dH (emu/g/T)')
plt.title('Ascending Branch Derivative with Cauchy Components (X K)')
plt.legend()
plt.grid(True)
plt.show()

# Step 13: Plot percent contributions as bar charts
fig, (ax1, ax2) = plt.subplots(1, 2, figsize=(12, 6))
n_comp_desc = len(percent_contrib_desc)
ax1.bar(range(n_comp_desc), percent_contrib_desc, color=['green', 'yellow',
'blue'][:n_comp_desc])
ax1.set_xticks(range(n_comp_desc))
ax1.set_xticklabels([f'P_{i+1}' for i in range(n_comp_desc)])
ax1.set_title('Descending Branch Percent Contributions (X K)')
ax1.set_ylabel('Percent Contribution (%)')
n_comp_asc = len(percent_contrib_asc)
ax2.bar(range(n_comp_asc), percent_contrib_asc, color=['green', 'yellow', 'blue'][:n_comp_asc])
ax2.set_xticks(range(n_comp_asc))
ax2.set_xticklabels([f'P_{i+1}' for i in range(n_comp_asc)])
ax2.set_title('Ascending Branch Percent Contributions (X K)')
ax2.set_ylabel('Percent Contribution (%)')
plt.tight_layout()
plt.show()
```

10 References

1. Benner, F.; Deshapriya, S.; Demir, S. A Tetraazanaphthalene Radical-Bridged Dysprosium Single-Molecule Magnet with a Large Coercive Field. *Chem. Sci.* **2025**, *16* (44), 20806–20822.
